# Recent and Rapid Assembly of an Island Species–Area Relationship Threatened by Human Disturbance

**DOI:** 10.1111/ele.70222

**Published:** 2025-10-05

**Authors:** Luiz Jardim de Queiroz, Timothy J. Alexander, Daniela Achleitner, Martin Luger, Hubert Gassner, Carmela J. Doenz, Soraya Villalba, Lukas Rüber, Rampal S. Etienne, Luis Valente, Ole Seehausen

**Affiliations:** ^1^ Naturalis Biodiversity Center Leiden the Netherlands; ^2^ Groningen Institute for Evolutionary Life Sciences (GELIFES) University of Groningen (RUG) Groningen the Netherlands; ^3^ Swiss Federal Institute of Aquatic Science and Technology (Eawag) Kastanienbaum Switzerland; ^4^ Institute of Ecology and Evolution (IEE) University of Bern (UniBe) Bern Switzerland; ^5^ Aquatic Consulting Bern Switzerland; ^6^ Federal Agency for Water Management Institute for Aquatic Ecology and Fisheries Management Mondsee Austria; ^7^ Federal Office for the Environment (FOEN), Swiss Confederation Ittigen Switzerland; ^8^ Naturhistorisches Museum Bern (NBME) Bern Switzerland

**Keywords:** adaptive radiation, Alps, endemism, extinction, fish, freshwater, glacial lakes, island biogeography, species introduction, species richness

## Abstract

The island species–area relationship (ISAR) describes how larger islands support more species. ISARs of isolated oceanic archipelagos, assembled over millions of years, typically show positive relationships, steep slopes, and species richness equilibrium. However, it remains unclear how quickly such characteristics emerge. We compiled a dataset for fish communities of 79 postglacial peri‐Alpine lakes and report an ISAR, formed *de novo* in less than 15,000 years, that partially mirrors older systems, but has an asymptotic shape. Immigration and speciation, the main ISAR drivers, are primarily associated with area and depth, respectively. Immigration increases with area, while speciation is promoted by greater depth, likely due to species depletion in the source pool and ecological constraints on speciation. This young ISAR has been reshaped by anthropogenic activities, with species introductions erasing its asymptotic shape. We demonstrate that ISARs can develop rapidly after insular habitat formation, offering insights into patterns of biodiversity assembly.

## Graphical abstract

The island species–area relationship (ISAR), traditionally studied in millions of years old oceanic archipelagos, remains poorly understood in much younger insular systems. Using fish communities from 79 postglacial peri‐Alpine lakes, we show that classical ISAR patterns can emerge rapidly, contrary to expectations given their young age (< 15,000 years). However, anthropogenic activities have significantly reshaped the natural ISAR. Our results demonstrate how quickly biodiversity patterns can develop and highlight the vulnerability of the peri‐Alpine lacustrine system to human disturbance.

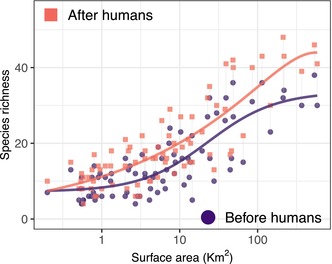

## 1 Introduction

The island species–area relationship (ISAR), which describes the species richness increase with island size, is a key macroecological pattern and the foundation of island biogeography theory (IBT; MacArthur and Wilson 1967). IBT hypothesises that island biodiversity is primarily influenced by island area and isolation. While immigration rates decrease with isolation, extinction rates decrease with area, and speciation accelerates with both increasing area and isolation (MacArthur and Wilson 1963, 1967; Valente et al. 2020). As a result of the distinct effects of immigration, extinction, and speciation, the shape of ISARs may differ among taxonomic groups, spatial scales and system ages (Whittaker et al. 2023).

While many types of insular systems exist, IBT has typically focused on oceanic islands systems that are often several million years old and rich in endemic species derived from in situ speciation (Whittaker et al. 2023). ISARs from such islands are often better represented by the power function, $S=c\times A^{z}$, or its log–log form, and tend to follow a predictable shape: positive relationship, steeper slopes than mainland SARs driven by an elevated level of endemism on larger islands, usually low intercepts, as few lineages from the source pool manage to reach and persist on smaller islands (Rosenzweig 2001; Tjørve et al. 2021; Whittaker et al. 2017), and no species saturation despite theoretical expectations (Lomolino 2000). These patterns are not expected on much younger archipelagos where there may not have been sufficient time for differential extinction and speciation to operate. Further, whether similar trends also occur in young island‐like systems such as lakes remains unknown.

Area has proven to be the best indicator of how much space and resources are available, and therefore of how many species an insular system can support. However, area alone often cannot explain all the variation observed in richness. A range of environmental insular features beyond area, such as time, energy, and topographic relief (Helmus et al. 2014; Roeble et al. 2024; Wagner et al. 2014), can influence colonisation, extinction, and in situ speciation, and consequently the resulting richness. Therefore, considering such variables in an island biogeography framework can be critical for improving our ability to predict richness (Triantis 2021).

Lakes can be regarded as “inverted islands”, being bodies of water surrounded by land. Yet, most lakes differ from oceanic islands in the degree of insularity, as rivers may function as “bridges” that reduce isolation. Furthermore, lakes often lack a clear *mainland* equivalent, unlike continents or large nearby islands are for oceanic islands. Instead, various connected aquatic environments (e.g., rivers, larger lakes, or the ocean) may serve as species sources. Lakes show, at least for fish, the highest speciation rates in aquatic systems (Miller 2021), host high levels of endemism and extraordinary adaptive radiations (Seehausen 2015; Wagner et al. 2014), resembling oceanic islands. This likely results from environmental stability and predictability at ecological time scales (McCune 1984), compared to rivers that feed or drain them (Seehausen 2015). Additionally, lakes uniquely offer a vertical dimension (depth), unavailable in rivers. Depth significantly expands volumetrically the available space for non‐bottom‐bound aquatic organisms more than elevation does for terrestrial species. In lakes, light, temperature, and oxygen concentration also change more rapidly along the vertical axis due to higher water density, but seasonal variation is dampened by the water column's height, creating stable depth gradients for new species to emerge and persist (Seehausen 2015; Wagner et al. 2014). Although the few existing ISAR assessments of lakes demonstrate an effect of area on species richness (Eadie et al. 1986; Illyes et al. 2023; Wagner et al. 2014), it remains unclear whether area or depth plays the primary role in facilitating in situ speciation (but see Hanly et al. 2017), or whether other lake features may also play a role.

The peri‐Alpine lacustrine system, spanning the Rhine, Rhône, Po, and Danube catchments, provides an excellent model for investigating ISAR dynamics in more recent insular, lacustrine systems. Formed after the Last Glacial Maximum (LGM), ~15,000 years ago (Seguinot et al. 2018), these lake archipelagos contrast with most oceanic islands, whose origins date back roughly three orders of magnitude earlier (~10 million years) (Valente et al. 2020), and differ from typical temperate postglacial lakes, which are usually species‐poor. Despite their relatively recent formation, the peri‐Alpine lakes host remarkable fish diversity, with over 100 native species (Alexander and Seehausen 2021; Zaugg and Huguenin 2018), which represent at least 20% of Europe's freshwater fish. Approximately one third of the species are endemic to the system, resulting from parallel adaptive radiations primarily among cold‐adapted lineages (*Coregonus*, *Salvelinus*, and *Cottus*) (Doenz et al. 2018; Freyhof and Kottelat 2007; Jardim de Queiroz et al. 2023; Lucek et al. 2018; Vonlanthen et al. 2009).

The modest isolation and young age of the peri‐Alpine lakes raise the question of whether a classical ISAR shape has developed despite recent formation. Early‐stage archipelagos display flat ISARs due to limited colonisation time (Whittaker et al. 2023). The rapidity of ISAR development depends, however, on colonisation, extinction (MacArthur and Wilson 1967), and speciation rates (Algar and Losos 2011). High colonisation rates should quickly yield equilibrium ISAR patterns (Simberloff and Wilson 1969, 1970), whereas low colonisation and speciation rates should produce distorted relationships (e.g., flat or weak, hump‐shaped or saturating ISARs). In the peri‐Alpine lakes, initial colonisation following glacial retreat was likely limited, as inhospitable conditions, such as high glacier‐derived turbidity early on, and low water temperatures persisting for a protracted time, should have hampered in situ speciation of early cold‐adapted colonists as well as the establishment of warm‐adapted fish species. As soon as turbidity ceased, the speciation rate among cold‐adapted fish likely increased, but restricted habitat availability and lake size should also have resulted in high extinction rates.

Human impact through urbanisation, pollution, habitat degradation and species introductions has affected the peri‐Alpine system, forming a potential additional influence on the lake‐SAR patterns observed today. Anthropogenic influences have significantly reshaped insular biodiversity globally by increasing colonisation via species introductions and elevating extinction rates (Fernández‐Palacios et al. 2021; Graham et al. 2017; Helmus et al. 2014). Incorporating this Anthropocene perspective into island biogeography can help interpret species richness patterns in the peri‐Alpine lakes (Matthews et al. 2023; Matthews and Triantis 2021; Russell and Kueffer 2019).

Here, we investigated the ISAR for fish species in the peri‐Alpine lakes across five countries (France, Switzerland, Italy, Germany, and Austria) to understand how the ISAR of a young insular system assembles. We compiled an extensive dataset of fish diversity and endemism to test: (i) which function best describes lake–SAR shape, (ii) which lake features determine richness and endemism, and (iii) to what extent anthropogenic activities have altered the natural lake–SAR. We expect our findings to shed light on how ISARs in young systems naturally develop, whether lakes function as islands, and how anthropogenic factors affect biodiversity in fragile insular ecosystems.

## 2 Material and Methods

### 2.1 Geographic Scope, Lake Features and Species Richness

We focus on 79 natural, peri‐Alpine lakes formed after the Last Glacial Maximum (LGM), spanning the basins of the Rhine, Rhône, Danube, and Po rivers (Data S2 and S3). We selected these lakes because comprehensive fish species inventories are available. We compiled a checklist of fish species for each lake, including native and endemic species (single‐lake and sister‐lake endemics). Species occurrence data (Data S3) are from previous inventories (Alexander and Seehausen 2021; Gassner et al. 2015; Luger et al. 2025). Most of these data were collected using standardised protocols (electrofishing along lake shores, benthic and pelagic gillnetting, and hydroacoustic surveys), with comparable sampling efforts across lakes. This consistency reduces potential sampling bias. We also compiled available data on non‐native species and those extinct or extirpated from individual lakes due to human activity. Non‐natives included translocated species native to one of the four basins but absent from a given lake, as well as introduced species that are native to basins outside the study area and have been brought by humans into the lakes.

### 2.2 Multimodel ISAR Analysis

The form of an ISAR is usually represented by the power model (Arrhenius 1921): $S=c\times A^{z}$, where *S* is species richness, and *A* is the area. After log‐transformation, the relationship becomes linear: $\log\;\left(S\right)=\log\;\left(c\right)+z\times \log\left(A\right)$, where log (*c*) and *z* are the intercept and the slope, respectively. The slope *z* indicates how richness changes with area, and steeper slopes are usually associated with a higher degree of island isolation (Rosenzweig 1995; Tjørve et al. 2021) and/or lower dispersal ability of the organisms (Tjørve and Turner 2009). The intercept (*c*) represents expected species richness as area approaches zero, reflecting the number of species that may occur in an area so small that no viable populations can persist and that, hence, must maintain themselves through dispersal into the area (Fattorini et al. 2017; Gould 1979; Tjørve et al. 2021).

We estimated the values of *z* and *c* of the power model for our entire dataset and separately for each catchment. This basin‐level analysis enabled us to investigate whether groups of lakes within basins show distinct ecological or biogeographical characteristics reflected in species richness patterns.

The power function assumes that richness increases indefinitely with area. A sigmoidal shape may be more appropriate at large spatial scales, as it accounts for initial slow growth on very small islands and species saturation as area increases (Lomolino 2000, 2001), although not necessarily all sigmoidal models impose a strict upper bound on richness. To address these factors and test whether the power function is the best model for our dataset, we fitted 24 models proposed as alternatives to describe ISARs (Dengler 2009; Gao et al. 2019; Matthews, Triantis, et al. 2019; Tjørve 2003; Triantis et al. 2012). These models cover a full range of shapes (linear, convex, and sigmoidal) and allow detection of saturation (asymptote). Four are piecewise linear models that account for one or two breakpoints in the curve (Tables S1–S4) (Gao et al. 2019; Matthews and Rigal 2021), where breakpoints indicate changes in slope and/or intercept within a single curve (Data S5).

We fitted these models in an untransformed space (i.e., no log‐transformations) using maximum likelihood, where the likelihood is the product of the Poisson probability of the observed richness values with the mean value given by the ISAR for each area. To avoid local likelihood maxima, we started optimisation from 10 random initial parameter sets. For piecewise models, threshold values were treated as free parameters and estimated during optimisation. We compared candidate ISAR models using the corrected Akaike Information Criterion (AICc). AICc weights were calculated to assess relative model support.

To assess effects of catchment bias on the best‐fitting model, we repeated the multimodel analysis four times, each time excluding one catchment.

### 2.3 Predictors of Richness and Endemism

Using generalised linear mixed models (GLMM), we tested whether factors other than lake area predict total native species richness (TNSR, i.e., including extinct species), proportion of endemic species (PEnS), and total native salmonid species richness (NSSR). We included NSSR because Salmonidae account for most adaptive radiations and endemism in these lakes.

We initially selected the following explanatory variables: surface area (km^2^), shoreline length (km), watershed area (km^2^), maximum depth (m), average depth (m), volume (m^3^), average lake surface temperature (°C), surface temperature range (°C), average air temperature (°C), air temperature range (°C), elevation (m), average steepness downstream river (°), steepness range (°), distance to the ocean (km), and distance to the nearest freshwater glacial refugium (km) (Data S1). We assessed multicollinearity among these variables by calculating variance inflation factors (VIF) using the ‘vif’ function from the car package v. 3.1.2 (Fox and Weisberg 2018). By applying a VIF threshold of 3.5, we retained surface area, maximum depth, average surface temperature, range of surface temperature, average steepness, steepness range, and distance to glacial refugium. Then, we fitted GLMMs using all possible combinations of these variables for each response variable (TNSR, PEnS, and NSSR). For each combination of variables, we ran models with and without catchment as a random variable. For PEnS and NSSR, both with many zeros, we also tested models excluding and including zero inflation.

We used a negative binomial distribution for TNSR and NSSR, and an ordered beta distribution for PEnS (Kubinec 2022). All variables were log‐transformed due to left skewedness and rescaled between 0 and 1. We fitted the models using the ‘glmmTMB’ function from the glmmTMB package v. 1.1.9 (Brooks et al. 2017), and selected the best model based on AICc. We calculated pseudo‐*R* ^2^ using the ‘squaredGLMM’ function from MuMIn v. 1.47.5 (Bartón 2023). For each best model, we reassessed collinearity among predictors using VIF and checked residuals with DHARMa v. 0.4.6 (Hartig 2022) (Data S4).

### 2.4 Effects of Anthropogenic Factors

To investigate to what extent anthropogenic factors have modified the ISAR, we used the “pre‐1900” fish community (TNSR; excluding introduced species and including extinct/extirpated ones) as a baseline. We then generated three “human‐modified” datasets: (i) original + introduced and translocated species, (ii) original—extinct species, (iii) original + introduced—extinct species. We applied the same methods to find the best‐fitting model for each modified dataset and fitted the power model to evaluate changes in slope (*z*) and intercept (*c*). Analyses were run for all lakes combined and by catchment using the ‘lin_power’ function from sars v 1.3.6 (Matthews, Triantis, et al. 2019).

## 3 Results

Total native fish species richness (TNSR) across the 79 peri‐Alpine lakes ranged from four (Offensee, Hintersteiner See, and Lake Silvaplana) to 38 species (Lake Constance). The number of endemic species and the proportion of endemic species (PEnS) were zero in 58 lakes, whereas the maximum number of endemic species and the highest PEnS were 11 and 0.31, respectively (both in Lake Thun).

Among the 24 models tested, the best fit to the relationship between lake area and TNSR, for all catchments combined, was the rational function (Figure 2A and Table S1), whereas the traditional power function ranked ninth. The rational function is represented by $S=\frac{c+z\times A}{1+d\times A}$, where *S* is the species richness and *A* is lake area. The intercept (*c*), which represents the baseline species richness as area approaches zero, was estimated at 7.23. The nonzero value of *c* indicates that species richness is not strictly area‐dependent. The parameter *z* (1.44) governs how quickly the numerator grows with increasing area. Finally, *d* (0.04) controls the shape of the curve and how quickly it saturates. The rational function is bounded because the denominator increases with area, preventing an unlimited rise in species richness. Specifically, as A→∞, species richness approaches an upper bound of S=z/d, which in this case is 1.443/0.043≃34species. When we tested for the best fit by sequentially excluding each catchment, the rational function consistently ranked highest. The only exception occurred when the Rhine lakes were excluded, in which case the power function provided the best fit (Table S2).

**FIGURE 1:**
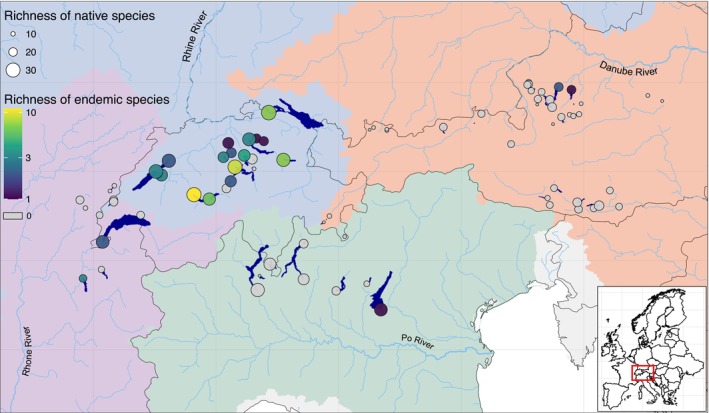
Distribution of species richness and endemism among fish communities in 79 peri‐Alpine lakes. Background colours represent different river basins. Circle size indicates the total number of native fish species; circle colour indicates the number of single‐lake endemic species, with grey indicating absence of single‐lake endemic species.

Although the power function was not the best fit for our combined data, we used its linearised form to enable comparison between catchments and with other systems. We obtained *c* and *z* values of 2.06 and 0.25, respectively (Figure 1A). When comparing catchments (Figure 1A), the steepest slope was observed for the Po lakes (*z* = 0.31), while the flattest was for the Rhône lakes (*z* = 0.14). The values of *z* for the Danube and Rhine lakes were 0.23 and 0.22, respectively. The highest intercept was found for the lakes of the Rhine catchment (*c* = 2.49), and the lowest was for the Po lakes (*c* = 1.58). The intercepts for the Rhône and Danube lakes were 2.17 and 1.96, respectively.

### 3.1 Predictors of Richness and Endemism

**FIGURE 2:**
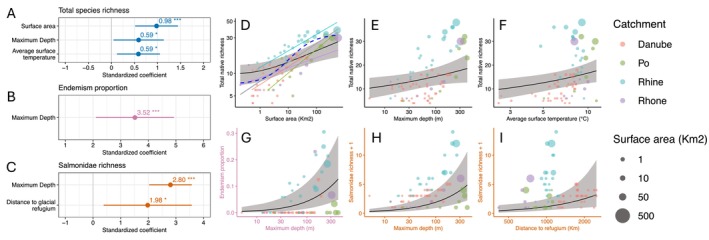
Relative importance of lake features for total native species richness of fishes, proportion of endemic species, and total salmonid species richness. Coefficient estimates for the best models of (A) total native species richness, (B) proportion of endemism, and (C) salmonid richness. The best models accounted for catchment as a random variable. Bars around coefficient estimates represent standard errors. Grey vertical line at 0 indicates no effect, and a variable with a positive coefficient indicates a positive effect on species richness or proportion of endemism. (D–I): Predictors of fish diversity. (D) The island species–area relationship (ISAR). Blue hatched line: Rational function, $S=\left(c+z\times A\right)/\left(1+d\times A\right)$, which was the best fit in a multimodel test using the entire dataset; Grey solid line: Fit of the log–log version of the power model for the entire dataset. Red, green, blue, and purple solid lines are the log–log power model fits for individual catchments. Solid black lines and shaded grey areas show the marginal effects of statistically significant environmental variables, accounting for the influence of other variables retained in the best‐fitting models to predict total native species richness (D–F), proportion of endemic species (G), and salmonid richness (H, I).

The preferred GLMMs for the richness metrics (TNSR, PEnS, and NSSR) treated catchment as a random variable. The fixed variables kept in the best model for TNSR included surface area, maximum depth, and average surface temperature (Figure 2A,D–F). TNSR correlated positively with all these variables. The overall variance (conditional *R* ^2^) explained in this model was 78%, and the variance explained by the fixed effects alone (marginal *R* ^2^) was 58%.

The best model describing PEnS did not account for zero inflation and contained only maximum depth as an explanatory variable, with a positive effect on PEnS (Figure 2B,G). The model explained 40.5% of the variance (conditional *R* ^2^), while the fixed effect accounted for 13.5% (marginal *R* ^2^). As for NSSR, zero inflation was also not considered in the best model, and both maximum depth and distance to glacial refugium were kept. Both had significant positive effects on NSSR (Figure 2C,H,I). The conditional *R* ^2^ for this model was 69.6%, while the marginal *R* ^2^ was 32.1%.

### 3.2 The Role of Anthropogenic Factors

The number of introduced (or translocated) species in a lake varied from zero (Almsee and Lauerzersee) to 17 (Lake Maggiore), whereas the number of extinct (or extirpated) species varied from zero (Almsee and Lauerzersee) to four (Attersee, Hintersteiner See, Offensee, Ossiachersee, Hallwilersee, and Lake Saint‐Point).

**FIGURE 3:**
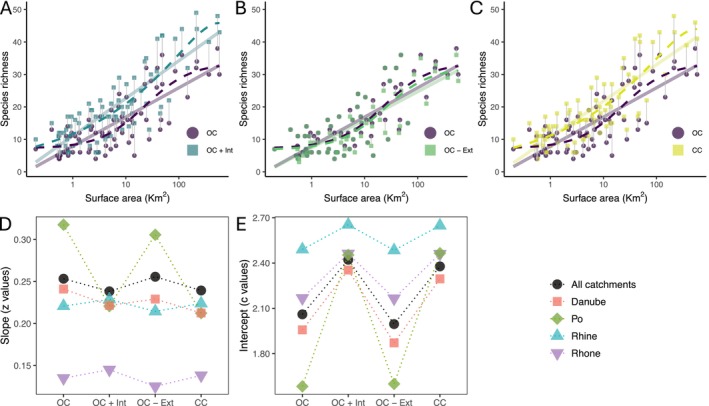
Effect of anthropogenic factors on the fish species–area relationship of the peri‐Alpine lakes. Comparison of the ISAR of the original community with the ISAR following anthropogenically‐caused species introductions (A), extinctions (B), and introductions + extinctions (C). Continuous lines represent the log–log power model fit for each dataset. Hatched lines represent the best‐fit function: (A, C) rational function for OC and persistence function 1 for OC + Int and CC, (B) rational function for both datasets. OC, original community; Ext, extinctions driven by anthropogenic factors; Int, non‐native species introductions or translocations; CC, current community, i.e., removing the extinct species and adding the introduced or translocated non‐native species. (D) Slope (*z* values) and (E) intercept (*c* values) from the log–log power model function applied to the species–area relationship under the different scenarios for the complete dataset and for each archipelago (catchment) independently.

The rational function (with asymptote) was still the best function to describe the ISAR when extinct species were excluded from the original community (Figure 3B and Table S5). However, the persistence function 1, with no asymptote, became the best function when introduced and translocated species were added to the original community (Figure 3A and Table S3) or when both introduced/translocated and extinct/extirpated species were considered (Figure 3C and Table S7).

We compared the features of a linearised power function of the ISAR for the original fish community with the features after human modifications. When considering introductions alone, the intercept increased (from 2.1 to 2.4), whereas the slope showed a minor decrease (from 0.25 to 0.24). Extinctions had no effect on the intercept or slope of the curves (Figure 3). When considering both processes (extinctions and introductions), we observe a similar pattern to that seen for species introductions only; that is, there was an increase in the intercept (2.1 to 2.4), but a minor decrease in the slope (0.25 to 0.24). The latter was driven mostly by a decrease in the slope in the Po archipelago, whereas the slope slightly increased in the Rhine and Rhône.

## 4 Discussion

Our study shows that, despite their geologically recent formation, the peri‐Alpine lake fish assemblages have developed an insular species–area relationship (ISAR) that partially mirrors those observed in much older oceanic island archipelagos, where there has been sufficient time for evolutionary processes to take place and for ecological saturation to be reached, resulting in a “mature” ISAR. The rapid build‐up of a qualitatively similar ISAR in the peri‐Alpine lakes in less than 15,000 years highlights how quickly communities can establish and still conform to classic biogeographic patterns following *de novo* habitat formation.

However, simulations show that such steep ISARs are not expected to develop rapidly on newly formed islands. In fact, in our simulations they typically only emerge after at least 1 million years (see Data S1 and Figure S6). Thus, the steep ISAR we observe is unexpected for a system of this age, suggesting unusually rapid ecological and/or evolutionary processes have operated in the peri‐Alpine lakes. We also tested whether passive sampling (random colonisation from a regional pool) could explain the observed ISAR by analysing rarefied richness data from a subset of lakes (Data S1). The results demonstrate that larger lakes support disproportionately more species than expected by passive sampling alone (Figure S7), allowing us to reject passive sampling as the main mechanism shaping the ISAR in this system.

### 4.1 On the Form of the Lake‐SAR and Inter‐Basin Variations

Fish species richness in the young peri‐Alpine lakes increases with lake area, consistent with IBT. The steepness of this increase mirrors patterns observed in much older oceanic island systems. However, unlike many of these older systems where a power function, implying no species saturation, often provides the best fit (Matthews and Rigal 2021; Triantis et al. 2012), our data were better described by a rational function with an upper asymptote, indicating that species richness approaches saturation as lake area increases. This asymptotic shape differs from typical older oceanic ISARs and likely reflects key differences in system characteristics. While one plausible explanation is that the peri‐Alpine system has reached equilibrium despite its young age, we argue that an important but not exclusive factor is a limited source species pool. As lake area increases, fish species richness approaches the total number of species available in the regional ‘mainland’ pool, suggesting that immigration has likely saturated. Speciation, on the other hand, may not have had sufficient time to fully overcome the limitations of the mainland pool size. Thus, although the shape of the ISAR of the young peri‐Alpine lakes diverges from classic old oceanic island models, especially due to saturation, the steepness still resembles that described in the latter.

Contrary to our expectations, piecewise models were not the best fit to our dataset, indicating no clear breakpoints for small lake effects or intensified speciation in larger lakes. One explanation for the latter may be that none of the peri‐Alpine lakes is sufficiently geographically isolated to reduce immigration rate to a degree that results in low competition among colonisers, allowing them to diversify. On the other hand, ecological isolation seems to be strong in the system, driven by depth and temperature, as many of the large lakes are very deep. Warm‐adapted lineages, especially cypriniforms, are limited to the surface, whereas only a few cold‐adapted lineages thrive in the deep waters where temperature varies around 5°C (Gaudard et al. 2019). Adaptive radiation in fish in the peri‐Alpine lakes has occurred exclusively across the depth gradient (Hudson et al. 2017; Vonlanthen et al. 2009). However, massive adaptive radiation, as observed in large tropical lakes (Seehausen 2006; Wagner et al. 2014), may not occur because the environment in the peri‐Alpine lakes is more homogeneous (Lewis 1996) and energy availability is lower in temperate systems (Davies et al. 2008). Consequently, the small fish radiations in the peri‐Alpine lakes are not sufficiently diverse to lead to a shift in the slope of the ISAR of the system nor even mask the saturating signal of total species richness indicated by the rational function.

Steep ISAR slopes (*z*) are common for systems where evolutionary dynamics (speciation) are relatively more important than immigration, a scenario that occurs with increasing isolation among island systems (Matthews et al. 2016; Matthews, Rigal, et al. 2019; "Rosenzweig 1995; Triantis et al. 2012). Compilations of *z*‐values for diverse insular systems corroborate this pattern, showing that oceanic archipelagos, typically the most isolated ones, yield higher *z*‐values (Matthews et al. 2016; Triantis et al. 2012). Our peri‐Alpine lacustrine system showed a *z*‐value of 0.25 for the native community, suggesting a much lower degree of isolation, akin to freshwater inland islands (islands located withing freshwater bodies) and fragmented habitats on continents.

If the slope of the ISAR reflects the degree of isolation of the system, the Po lakes appear to be the most isolated, as they exhibited the highest *z*‐value (0.3). Yet, greater isolation typically leads to a higher proportion of endemism due to more frequent in situ speciation. This pattern, however, was not observed, as fish in the Po lakes have not undergone adaptive radiation comparable to those of the Rhine, Rhône, and Danube lakes. This paradox may stem from historical biogeographic constraints. Notably, *Coregonus* and *Salvelinus*, two of the most species‐rich genera in the peri‐Alpine lakes and known for their propensity to diversify along depth gradients, have never naturally colonised the lakes in the Po catchment. In their absence, and despite isolation, no other lineage naturally present in the Po lakes appears to have had the same evolutionary potential to boost endemism in these lakes although endemic deepwater sculpins occur in two and an endemic deepwater trout in one lake.

### 4.2 Predictors of Fish Richness and Endemism

While assessing predictors of fish diversity, the most striking difference was the absence of area as an important predictor for the proportion of endemic species (PEnS) and native salmonid richness (NSSR). Salmonids comprise most of the endemics in the system as a product of parallel adaptive radiations (Doenz et al. 2018; Vonlanthen et al. 2009, 2012). Therefore, these results suggest that the speciation assembly component responds primarily to depth and is less dependent on area. This is because adaptive radiations of fish in these lakes have occurred mostly across depth gradients. Immigrant species that have not radiated, on the other hand, tend to be confined to shallow water, explaining why area shows a better fit than depth as a predictor of total richness. These findings show how different ecological and evolutionary processes respond to distinct environmental factors, supporting recent calls to move from purely geographic to environmentally explicit biodiversity analyses (Graham et al. 2025).

Lacustrine fish adaptive radiation is indeed highly dependent on depth (Seehausen and Wagner 2014; Smith and Todd 1984). Therefore, one should expect that the general rule would be depth as the best predictor of fish endemism in lakes, as we suggested here for the peri‐Alpine lakes. A continental analysis of cichlid fish in African lakes pointed in this direction (Wagner et al. 2014). Furthermore, lakes with similar area (321–600 km^2^) and age (~15 Ky) near the peri‐Alpine system, but much shallower (maximum depth < 12 m) (e.g., Lake Neusiedl, Austria; Lake Balaton, Hungary) (Bíró 1997; Draganits et al. 2022), show total native richness (30–40 species) equivalent to peri‐Alpine lakes with similar area, but do not contain endemics, likely due to limited depth. However, a previous global study showed that the probability of endemism does not increase with depth (Hanly et al. 2017). That study used binary endemism presence/absence, while our use of endemism proportion may reveal different patterns at the global scale. Future efforts should concentrate on compiling both total and endemic fish richness in lakes on a global scale, helping us understand if and how lake area and depth can constrain or facilitate immigration, speciation and coexistence.

### 4.3 Anthropogenic Impacts

Islands have been altered by human activities, accelerating extinctions (Sayer et al. 2025) and colonisation of non‐native species (Chown et al. 1998; Jeschke et al. 2014). The facilitation of colonisation through species introductions weakens isolation, and therefore decreases ISAR's slope if species introductions occur independently of area, as is the case for *Anolis* communities in the Caribbean (Helmus et al. 2014). However, where human‐driven extinction has decreased native biodiversity and, consequently, also flattened the ISAR slope, introduced species may simply restore the ISAR to its original form (Matthews et al. 2023).

The most striking effect of the introduction of non‐native species observed in the peri‐Alpine lakes was the elimination of the upper asymptotic limit of the ISAR. This supports our hypothesis that the asymptote of the native community arose as a consequence of a restricted set of species in the source pool, a restriction that was removed by human introductions. When species originating outside this constrained pool are introduced into the system, the previously upper boundary of the observed species richness is erased.

When we focus on the classic power function, we observe a slight decrease in the slope of the ISAR, and a substantial increase in the intercept. This aligns with the disappearance of the saturation point observed in the best‐fitting function, indicating that isolation has been somewhat weakened, and suggesting that species introductions have been overall homogeneous across lakes of all sizes. In fact, the absolute number of non‐native species is positively correlated with area or maximum depth (Figure S4A,B). However, in terms of proportion, the increase of species due to introductions does not depend on area or maximum depth (Figure S4C,D), which explains the substantial increase in the intercept.

Extinction alone did not affect the features of the lake‐SAR, as the slope and intercept were not substantially changed. This might be the result of a homogeneous extinction process occurring across lakes, instead of being prevalent in smaller or larger ones. In fact, we observe that the absolute number of extinct species scales weakly with lake area (Figure S4E), indicating that factors independent of lake area are likely at play. For birds on terrestrial islands, however, a decrease in the slope of the ISAR was found, which was attributed to more frequent human‐induced extinctions on larger islands (Matthews et al. 2023). However, note that they documented a substantially higher number of extinct species than we did, which may help explain the contrasting patterns observed between their study and ours.

Although we were careful in our assessment of species introductions and extinctions, we cannot completely rule out a bias in the dataset. The lack of an effect of extinctions could also be explained by an underestimate of the number of extinctions, as historical records are likely incomplete, and there is variation in knowledge among catchments (Data S1).

## 5 Final Considerations

Here, we show that the recent formation of the peri‐Alpine lakes has not hindered the development of a classical, “mature” ISAR for a key component of lacustrine biodiversity: the fish fauna. In the context of island biogeography, this pattern is surprising given the young geological age of the system, as our simulations indicate that such steep ISARs are generally only expected after much longer timescales. Another key result is that colonisation‐driven richness, based on the total richness analysis, correlates most strongly with lake area, while speciation‐driven richness (leading to endemism) is more closely linked to lake depth, a key measure of environmental heterogeneity. This suggests that larger lakes mainly accumulate species through colonisation, whereas deeper lakes facilitate in situ speciation by offering more diverse and stratified habitats.

The most notable anthropogenic impact was the alteration of key features of the natural ISAR for the fish communities in the peri‐Alpine lacustrine system, specifically, the removal of the upper limit (asymptote) of the ISAR following species introductions, along with an increase in the intercept of the curves. These changes highlight an underlying vulnerability of the system to human pressures and raise concerns about how further disturbances might reshape biodiversity patterns. A deeper understanding of the processes driving ISAR formation (colonisation, extinction, and in situ speciation) across these lakes will be crucial for predicting future responses of lacustrine biodiversity to environmental change and for informing effective conservation strategies.

While our study focuses on a relatively species‐rich postglacial lake system, many postglacial lakes, especially at higher latitudes, tend to be species‐poor due to limited colonisation opportunities due to long distances from larger southerly species pools and particularly small regional species pools. As such, the applicability of our findings may vary with differences in regional species richness, with dynamics like colonisation and extinction potentially having stronger effects on the ISAR shape in more depauperate systems. Such regional variation underscores the value of comparing lakes in a diversity of geographical settings to understand how species–area relationships manifest under a broad range of ecological and evolutionary conditions and constraints.

## Author Contributions

L.J.Q., R.S.E., L.V., and O.S. conceived the study. L.J.Q. curated the data with contributions from T.J.A., D.A., M.L., H.G., C.J.D., S.V., L.R., and O.S., L.J.Q., T.J.A. and R.S.E. conducted the formal analyses. Funding was secured by L.J.Q., L.V., R.S.E., and O.S. The investigation was carried out by all authors. L.J.Q., R.S.E., and O.S. developed the methodology. D.A., M.L., H.G., L.R., and O.S. coordinated project administration. R.S.E., L.V., and O.S. provided resources. L.J.Q. and R.S.E. developed the software and led data validation and visualisation. L.J.Q., L.V., and O.S. prepared the original draft, and all authors contributed to the review and editing of the manuscript. All authors approved the final version for submission.

## Peer Review

The peer review history for this article is available at https://www.webofscience.com/api/gateway/wos/peer‐review/10.1111/ele.70222.

## Supporting information


**Data S1:** ele70222‐sup‐0001‐Supinfo1.pdf.

**Data S2:** ele70222‐sup‐0002‐Supinfo2.xlsx.

**Data S3:** ele70222‐sup‐0003‐Supinfo3.xlsx.

**Data S4:** ele70222‐sup‐0004‐Supinfo4.html.

**Data S5:** ele70222‐sup‐0005‐Supinfo5.html.

## Data Availability

The data supporting the findings of this study, as well as the code used for the analyses and figure generation, have been uploaded to Dryad at https://doi.org/10.5061/dryad.q2bvq83xm. They will be made publicly available upon publication. During the review process, the data can be accessed via the following temporary link: http://datadryad.org/share/9tf9BTW‐KejIqS_1DhifwP0v7OSA_TsPufQQqjAZDS4.
