# Supplementary material for: Recent and Rapid Assembly of an Island Species–Area Relationship Threatened by Human Disturbance
Source: Ecol Lett. 2025 Oct 5;28(10):e70222. doi: 10.1111/ele.70222 (PMC12498083; doi:10.1111/ele.70222)
Supplement: Supplementary file 1 — Data S1: ele70222‐sup‐0001‐Supinfo1.pdf. [file ELE-28-0-s001.pdf]

SUPPORTING INFORMATION

Jardim-de-Queiroz L et al. 2025. Recent and rapid assembly of an island species–area relationship in postglacial lakes threatened by human disturbance. *Ecology Letters.*

Supporting information S1

Geographic scope and lake features

Although thousands of lakes are part of the hydrographic network in the Alps, we focus on 79 natural, peri-Alpine postglacial lakes for which robust fish species lists have been previously compiled. We define peri-Alpine lakes as those that are near or below the tree line or in the foothills of the Alps, often in regions that transition from mountainous to flatter terrain, and particularly at the lower end of alpine valleys carved by glaciers in a fjord-like manner. In contrast to alpine lakes (lakes located above the tree line globally), fish can usually colonize subalpine (perialpine) lakes naturally because the outlets of these lakes to rivers are open. In the Alps, some lakes could also be considered peri-Alpine due to their geographical position (e.g. Neusiedler See), but we included only lakes that originated through glacier activity, and were, therefore, formed after the Last Glacial Maximum (LGM).

The lakes used in the present work span four main drainage basins, three of which lie along the axis west–north–east of the Alps (Rhine, Rhone, and Danube), and one to the south (Po). In total, our database contains 22 lakes from the Rhine, eight from the Rhone, nine from the Po, and 40 from the Danube. These catchments are currently isolated from one another, though lakes within each basin are directly fed by a dense river network, creating a system with high intra-basin connectivity (Preusser 2008; Sommerwerk *et al.* 2009). Moreover, palaeo-hydrographic reconstructions of the area indicate a history of connections and disconnections among basins, whereby one river or stream is diverted from its original course and captured by another, i.e. river captures (Gibbard *et al.* 1988; Winterberg & Willett 2019).

In the present study, we consider the four main drainage basins either as a single pan-Alpine system or as separate “archipelagos” of lakes.

For each lake, we compiled a set of environmental variables: surface area (km^2), shoreline length (km), watershed area (km^2), maximum depth (m), average depth (m), and volume (m^3) from HydroLakes (Messager et al. 2016); annual average surface temperature (°C) and annual range surface temperature (°C) from EarthEnv (Domisch et al. 2015); average (Bioclimatic variables 1: Bio01) and range air temperature (Bio07) (°C) from CHELSA (Karger et al. 2020, Brun et al. 2022). Temperature variables are based on historical data from 1970–2000. To quantify isolation, we measured elevation (m) and both the average steepness and the steepness range of the terrain (the angle between the terrain and a horizontal plane, calculated along the shortest path from the lake’s main outlet to ocean); as well as distance to the nearest open freshwater glacial refugium (km), and distance to the ocean (km). The latter two metrics were computed by tracing the main contemporary channel of the rivers, for which we used the function ‘riverdistance’ from the R package ‘riverdist’ v. 0.16.3 (Tyers 2024). In geological literature, the steepness of the terrain is typically referred as slope; we chose, however, the term "steepness" to avoid confusion with the ISAR’s slopes. The database is provided in Supporting Information S2.

Regardina the isolation metrics:

- We considered *distance to the ocean* (km), which should indicate how lakes are connected to downstream systems, and *distance to putative glacial refugia* (km), which reflects historical accessibility to colonizing lineages at broader spatial scales. Both distances were computed by tracing the main contemporary channel of the rivers, for which we used the function ‘riverdistance’ from the R package ‘riverdist’ v. 0.16.3 (Tyers 2024).

- In geological literature, the steepness of the terrain is typically referred to as slope; we chose, however, the term "steepness" to avoid confusion with the ISAR's slopes. The database is provided in Supporting Information S2. Steepness was measured along the course of contemporary river networks.
- To capture more recent limitations to connectivity, we included *elevation* and *topographic steepness* as proxies for colonization resistance from nearby water bodies. These variables are especially relevant because elevation gradients and rugged terrain can strongly constrain fish dispersal along river systems, particularly in the upstream direction (Jaramillo-Villa *et al.* 2010; Suvarnaraksha *et al.* 2012).
- Additionally, we considered *watershed area*. While often interpreted as a proxy for size, watershed area can also serve as an effective surrogate for riverine connectivity: it reflects the number and size of inflowing streams, indicates a lake's position within the drainage network (with larger watersheds typically linked to more connected, downstream locations), and captures cumulative upstream connectivity by representing the spatial extent from which colonizing lineages could potentially arrive.

Glacial refugia: The nearest freshwater refugium was assumed to represent a potential source of colonization after the end of the LGM. The following hypothetical refugia were used: for the lakes in the Rhone catchment, we used the Upper Rhone refugium proposed for sticklebacks (*Gasterosteus aculeatus*) (Mäkinen *et al.* 2006) for the Rhine, we used the Elbe–Main refugium proposed for sculpins (*Cottus gobio*) (Hänfling *et al.* 2002); for the Po lakes, we used the Balkan Peninsula refugium also proposed based on the three-spined stickleback (*Gasterosteus aculeatus*) (DeFaveri *et al.* 2012); and for the Danube lakes, we used the Danube refugium, which was proposed based on the European chub (*Squalius cephalus*) (Cortés-Guzmán *et al.* 2024). We also selected distance to the ocean (North Sea for the Rhine; Mediterranean Sea for the Rhone; Adriatic Sea for the Po; Black Sea for the Danube) as a variable because modern shorelines are believed to have served as freshwater habitats during the LGM, when sea level was lower and exposed continental shelves (Faure *et al.* 2002; Post *et al.* 2013; Thomaz *et al.* 2015; Toucanne *et al.* 2010).

Fish dataset

We compiled a regional checklist of fish species native to each of the 79 peri-Alpine, postglacial lakes. Our checklist covers all fish lineages, which belong to two classes, Petromyzontida (jawless fishes) and Actinopterygii (ray-finned fishes; Chondrostei and Teleostei). We compiled most of the species occurrence data from previous inventories that were based on an exhaustive literature search (Alexander & Seehausen 2021; Gassner *et al.* 2015; Luger *et al.* 2025) (Alexander & Seehausen 2021; Gassner *et al.* 2015; Luger *et al.* 2025).

These inventories also compiled a list of non-native species in each lake based on historical information. The non-native species included both translocated and introduced species. We consider as translocated species those that are native to one of the four basins (Rhine, Rhone, Danube, or Po) but were not naturally present in a specific lake. For instance, the catfish *Silurus glanis* is native to lakes and rivers in the Upper Rhine and Danube but was translocated into the Rhone, including Lake Geneva; the whitefish *Coregonus lavaretus* is endemic to Lake Bourget (Rhone Basin), but stocks have been introduced into Lake Saint-Point (also in the Rhone Basin). Introduced species are those native to basins outside the four addressed in this study and brought by humans to lakes where they did not previously occur. Examples include the lake trout (*Salvelinus namaycush*), native to North American drainages, and the stone moroko (*Pseudorasbora parva*), originally from East Asian drainages. Note that

although *Coregonus acrinasus* and *C. suspensus* are technically endemic to Lakes Thun and Lucerne, respectively, these species probably emerged through hybridization between a native species and a translocated one. Therefore, their origins were likely human-mediated (Selz *et al.* 2020), and we classified them as non-native species.

The number of documented extinctions or extirpations per lake was also obtained from three main inventories (Alexander & Seehausen 2021; Gassner *et al.* 2015). These compilations provided a list of extinctions or extirpations mostly driven by eutrophication (nutrient enrichment of the lakes, leading to oxygen depletion and excess turbidity) or overfishing.

For lakes Sempach, Aegeri, Lauerzersee, Greifen, Baldegg, Silvaplana, and Pfäffiker, we compiled a list based on additional bibliography (Freyhof & Kottelat 2007; Vonlanthen *et al.* 2019; Zaugg & Huguenin 2018).

When ISARs start to deviate from being flat

In our assessment of the fish communities in peri-Alpine lakes, we argue that the observed island species–area relationship (ISAR) is unexpectedly steep for such a young system (less than 15,000 years old). To provide a theoretical expectation for how ISARs develop over time, we simulated ISARs for oceanic islands at various time slices (ranging from 0.001 to 1,000 million years old) using the DAISIE model (Valente *et al.* 2015). The parameters to build such ISARs were estimated from empirical avian data spanning 41 archipelagos worldwide (Valente *et al.* 2020). Specifically, we fitted Valente et al.'s M14 model, but excluded distance-dependence in colonization and anagenesis. The resulting simulated ISARs therefore reflect expectations for oceanic islands at different stages of geological and ecological development.

Our simulations reveal that the ISAR remains essentially flat during the early stages of island development, only becoming noticeably positive from around 1 million years onwards (Figure S6). Therefore, the pronounced ISAR observed for fish in the peri-Alpine lakes is inconsistent with what would be expected for a system that is geologically very young.

Testing for passive sampling

Passive sampling occurs when species colonize islands randomly from a regional species pool, with larger islands receiving more colonizing individuals purely due to their greater size. Under passive sampling, larger islands are expected to accumulate more individuals and therefore more species simply by chance, without invoking additional ecological or evolutionary processes. Therefore, random sampling alone can generate a steep ISAR relatively quickly, on ecological timescales (Gooriah & Chase 2020).

To investigate whether passive sampling is potentially the main driver of the ISAR of the peri-Alpine lakes, we compiled a lake-by-species matrix indicating species presence and abundance from a subset a total of 33 lakes for which species abundance was available from standardized sampling effort (Alexander & Seehausen 2021). Then, we calculate rarefied species richness (Sn) using the ‘rarefy’ function from vegan v. 5.8.1 (Paradis & Schliep 2019) by standardizing richness to the minimum total abundance observed in any lake. We then tested the ISAR of these 33 lakes by regressing the rarefied richness against the log-transformed lake surface area using a linear model. If there is a significant positive correlation between Sn and lake area, then we can reject the passive sampling hypothesis.

For our subset of lakes, the rarefied species richness, standardized to a common number of individuals across lakes, increased significantly with lake area (linear model: Sn ~ log(area), slope = 3.86 ± 0.62 SE, $t = 6.22$, $p < 0.001$, $R^2 = 0.56$, $n = 33$ lakes; Figure S7). This

result indicates that, even after accounting for differences in sampling effort, larger lakes support disproportionately more species than expected from passive (random) sampling alone.

Furthermore, we reject the hypothesis that within-lake heterogeneity influenced our overall patterns, as the sampling strategy incorporated a wide range of methods and covered multiple locations within each lake, from shallow to deep waters and from the shoreline to the lake center.

On other potential biases

By catchment

Our multimodel analyses showed that the rational function consistently emerged as the best-fitting model, even when one catchment was excluded at a time (Table S2). The only exception occurred when lakes from the Rhine catchment were removed, in which case the power function provided the best fit to the ISAR. We interpret this shift not as a sampling bias but as a reflection of the underlying biogeographic structure of the peri-Alpine lake system. Because most of the largest and deepest lakes are located in the Rhine catchment, their exclusion likely removes lakes at the upper end of the species–area distribution. Consequently, it is natural that the characteristic saturation captured by the rational function becomes undetectable, and a non-asymptotic model better represents the remaining pattern.

In extinction records

The lack of an extinction effect on ISAR shape could be explained by an underestimation of extinction events, as historical records are likely incomplete. One possible source of this underestimation may relate to lake size. Most documented extinctions date to the past few centuries, yet occupation by farmers, modern humans, and landscape modification in the peri-Alpine region began in the Early Neolithic (ca. 5500–4500 BCE) and intensified during the expansion of the Roman Empire (Gilck & Poschlod 2019) Human-driven fish extinctions from these earlier periods are likely undocumented. Moreover, most known extinctions involve species of commercial importance to regional fisheries. These species are typically more abundant, and better monitored, especially in larger lakes. As a result, extinctions or local extirpations of less economically valuable species, especially those occurring in smaller or less intensively studied lakes, may have gone unnoticed.

To address potential bias from lake size, we reanalyzed the ISAR by progressively removing small lakes. Excluding lakes smaller than 1 km² produced no change, as the rational function remained the best fit for both datasets (original richness and richness after removing known extinct species) (Fig. S5). However, when lakes smaller than 5 km² were excluded, the logarithmic function best described the original community, but with very similar AIC weights to the Kobayashi function (0.138 vs. 0.137, respectively). The Kobayashi function provided a better fit for the dataset excluding extinct species (Fig. S5), but once more, it showed AIC weights very close to the logarithmic function (0.1277 vs. 0.1276, respectively). These results indicate that any bias in extinction records caused by lakes smaller than 5 km² is limited.

Another source of bias could be related to differential knowledge of the lakes across catchments (i.e., some catchments may be better known than others). To address this potential bias, we reanalyzed the ISAR by sequentially removing one catchment at a time (Tables S2 and S6). In most cases, the rational function remained the best-fitting model for both the original community (Table S2) and the dataset excluding extinct species (Table S6). The only notable exception occurred when lakes from the Rhine catchment were excluded. In this case, the

power function provided the best fit to the original community, while the Power Rosenzweig function provided the best fit for the dataset without extinctions. This difference, as before, may be explained by the removal of the Rhine lakes, which are the largest and deepest ones, thereby removing the lakes at the upper end of the ISAR. Overall, these results suggest that extinction does not substantially alter the shape of the ISAR, as the same function generally best describes both datasets regardless of which catchment is excluded.

In endemism records

Figure 1 of the main manuscript shows the proportion of endemic species per lake. One pattern that emerges is that a high proportion of endemic species comes from Switzerland, raising the question of whether this is related to sampling effort. This is likely because many of the largest and deepest lakes in the peri-Alpine region, where adaptive radiation led to the emergence of endemic species, are located within Swiss territory. Importantly, lakes in the French and Italian regions (corresponding broadly to the Rhone and Po basins, respectively) have received comparable sampling effort over the past decades, as documented in the *Projet Lac* synthesis report (Alexander and Seehausen, 2021).

However, we cannot completely rule out a potential source of bias stemming from a comparatively lower intensity of molecular studies in Austrian lakes (Danube basin), which could have limited the detection of cryptic endemic species, particularly among *Coregonus* radiations, which may be less well studied genomically than their counterparts in the Rhine and Rhone. It is also worth noting that the Danube lakes are, on average, the shallowest in our dataset, which could explain the lower absolute number of endemic species. This does not necessarily translate into a lower proportion of endemic species, but it may still contribute to the observed patterns.

To explicitly account for regional differences, we included catchment identity as a random effect in our mixed-effects models when testing for the best set of variables to explain the proportion of endemic species. This approach helped address catchment-specific sampling bias. Nonetheless, we re-ran the models excluding the Danube lakes to assess the robustness of our results. The model rankings and parameter estimates remained consistent, and the best-supported model and conclusions were unchanged. Given that the catchment random effect already accommodates such variation, we did not report the results of this reduced analysis in the main text.

References

Alexander, T. & Seehausen, O. (2021). *Diversity, distribution and community composition of fish in perialpine lakes. “Projet Lac” synthesis report*. Eawag: Swiss Federal Institute of Aquatic Science and Technology.

Cortés-Guzmán, D., Sinclair, J., Hof, C., Kalusche, J.B. & Haase, P. (2024). Dispersal, glacial refugia and temperature shape biogeographical patterns in European freshwater biodiversity. *Glob. Ecol. Biogeogr.*, e13886.

DeFaveri, J., Zanella, L.N., Zanella, D., Mrakovčić, M. & Merilä, J. (2012). Phylogeography of isolated freshwater three-spined stickleback Gasterosteus aculeatus populations in the Adriatic Sea basin. *J. Fish Biol.*, 80, 61–85.

Faure, H., Walter, R.C. & Grant, D.R. (2002). The coastal oasis: ice age springs on emerged continental shelves. *Glob. Planet. Change*, The global carbon cycle and its changes over glacial-interglacial cycles, 33, 47–56.

Freyhof, J. & Kottelat, M. (2007). *Handbook of European freshwater fishes*.

Gassner, H., Achleitner, D. & Luger, M. (2015). *Guidance on srveying the biological quality elements Part B1 – Fish*. Austrian Federal Ministry of Agriculture and Forestry, Environment and Water Management; BAW – Institute for Water Ecology, Fishery Biology and Limnology, Vienna.

Gibbard, P.L., Rose, J., Bridgland, D.R., Shackleton, N.J., West, R.G. & Bowen, D.Q. (1988). The history of the great northwest European rivers during the past three million years. *Philos. Trans. R. Soc. Lond. B Biol. Sci.*, 318, 559–602.

Gilck, F. & Poschlod, P. (2019). The origin of alpine farming: A review of archaeological, linguistic and archaeobotanical studies in the Alps. *The Holocene*, 29, 1503–1511.

Gooriah, L.D. & Chase, J.M. (2020). Sampling effects drive the species–area relationship in lake zooplankton. *Oikos*, 129, 124–132.

Hänfling, B., Hellemans, B., Volckaert, F. a. M. & Carvalho, G.R. (2002). Late glacial history of the cold-adapted freshwater fish *Cottus gobio*, revealed by microsatellites. *Mol. Ecol.*, 11, 1717–1729.

Jaramillo-Villa, U., Maldonado-Ocampo, J.A. & Escobar, F. (2010). Altitudinal variation in fish assemblage diversity in streams of the central Andes of Colombia. *J. Fish Biol.*, 76, 2401–2417.

Luger, M., Kammerlander, B., Pamminger-Lahnsteiner, B., Achleitner, D. & Gassner, H. (2025). Die Fischgemeinschaften in österreichischen Seen >50 ha: Erhebung und Bewertung nach EU-WRRL und ALFI (Austrian Lake Fish Index). *Österr. Wasser- Abfallwirtsch.*

Mäkinen, H.S., Cano, J.M. & Merilä, J. (2006). Genetic relationships among marine and freshwater populations of the European three-spined stickleback (Gasterosteus aculeatus) revealed by microsatellites. *Mol. Ecol.*, 15, 1519–1534.

Paradis, E. & Schliep, K. (2019). ape 5.0: an environment for modern phylogenetics and evolutionary analyses in R. *Bioinformatics*, 35, 526–528.

Post, V.E.A., Groen, J., Kooi, H., Person, M., Ge, S. & Edmunds, W.M. (2013). Offshore fresh groundwater reserves as a global phenomenon. *Nature*, 504, 71–78.

Preusser, F. (2008). Characterisation and evolution of the River Rhine system. *Neth. J. Geosci.*, 87, 7–19.

Selz, O.M., Dönz, C.J., Vonlanthen, P. & Seehausen, O. (2020). A taxonomic revision of the whitefish of lakes Brienz and Thun, Switzerland, with descriptions of four new species (Teleostei, Coregonidae). *ZooKeys*, 989, 79–162.

Sommerwerk, N., Hein, T., Schneider-Jakoby, M., Baumgartner, C., Ostojić, A., Paunović, M., *et al.* (2009). *The Danube River Basin*. London : Delhi: Elsevier.

Suvarnaraksha, A., Lek, S., Lek-Ang, S. & Jutagate, T. (2012). Fish diversity and assemblage patterns along the longitudinal gradient of a tropical river in the Indo-Burma hotspot region (Ping-Wang River Basin, Thailand). *Hydrobiologia*, 694, 153–169.

Thomaz, A.T., Malabarba, L.R., Bonatto, S.L. & Knowles, L.L. (2015). Testing the effect of palaeodrainages versus habitat stability on genetic divergence in riverine systems: study of a Neotropical fish of the Brazilian coastal Atlantic Forest. *J. Biogeogr.*, 42, 2389–2401.

Toucanne, S., Zaragosi, S., Bourillet, J.-F., Marieu, V., Cremer, M., Kageyama, M., *et al.* (2010). The first estimation of Fleuve Manche palaeoriver discharge during the last deglaciation: Evidence for Fennoscandian ice sheet meltwater flow in the English Channel ca 20–18 ka ago. *Earth Planet. Sci. Lett.*, 290, 459–473.

Tyers, M. (2024). riverdist: River Network Distance Computation and Applications.

Valente, L., Etienne, R.S. & Dávalos, L.M. (2017). Recent extinctions disturb path to equilibrium diversity in Caribbean bats. *Nat. Ecol. Evol.*, 1, 0026.

Valente, L., Phillimore, A.B., Melo, M., Warren, B.H., Clegg, S.M., Havenstein, K., *et al.* (2020). A simple dynamic model explains the diversity of island birds worldwide. *Nature*, 579, 92–96.

Valente, L.M., Phillimore, A.B. & Etienne, R.S. (2015). Equilibrium and non-equilibrium dynamics simultaneously operate in the Galápagos islands. *Ecol. Lett.*, 18, 844–852.

Vonlanthen, P., Kreienbühl, T. & Périat, G. (2019). *Standardisiert Befischung Sempachersee: Resultate der Erhebungen vom September 2018*. Aquabios GmbH, Sursee.

Winterberg, S. & Willett, S.D. (2019). Greater Alpine river network evolution, interpretations based on novel drainage analysis. *Swiss J. Geosci.*, 112, 3–22.

Zaugg, B. & Huguenin, K. (2018). *Fauna Helvetica: Pisces — Guide d'identification/Bestimmungshilfe.* CSCF&SEG. Fauna Helvetica 30, info fauna CSCF & SEG, Neuchâtel.

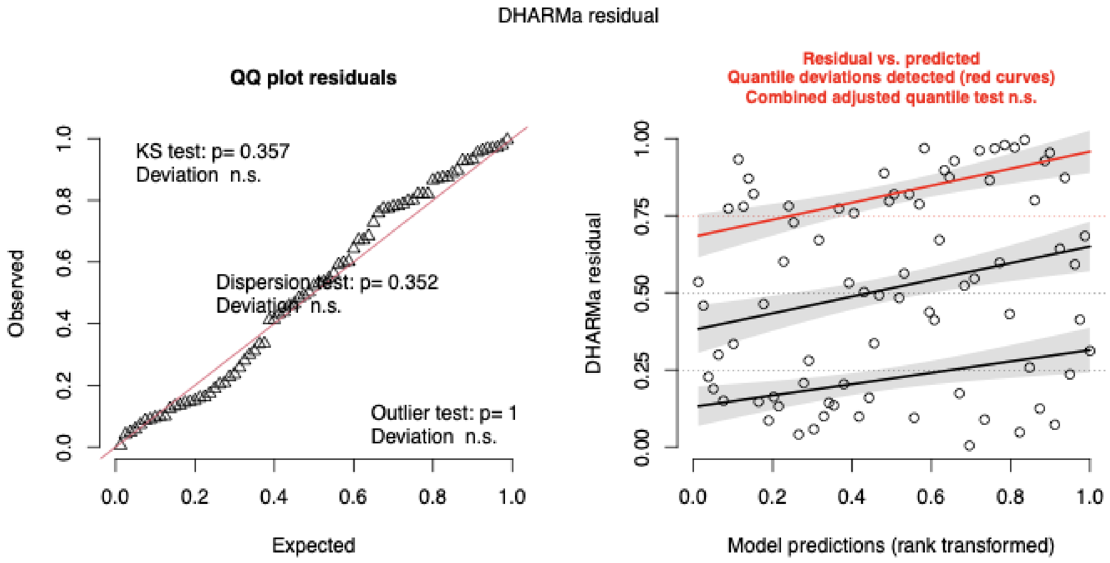

Figure S1. DHARMa residual check for the total native species richness (TNSR) best model. Variance Inflation Factors varied from 1.57 to 4.33.

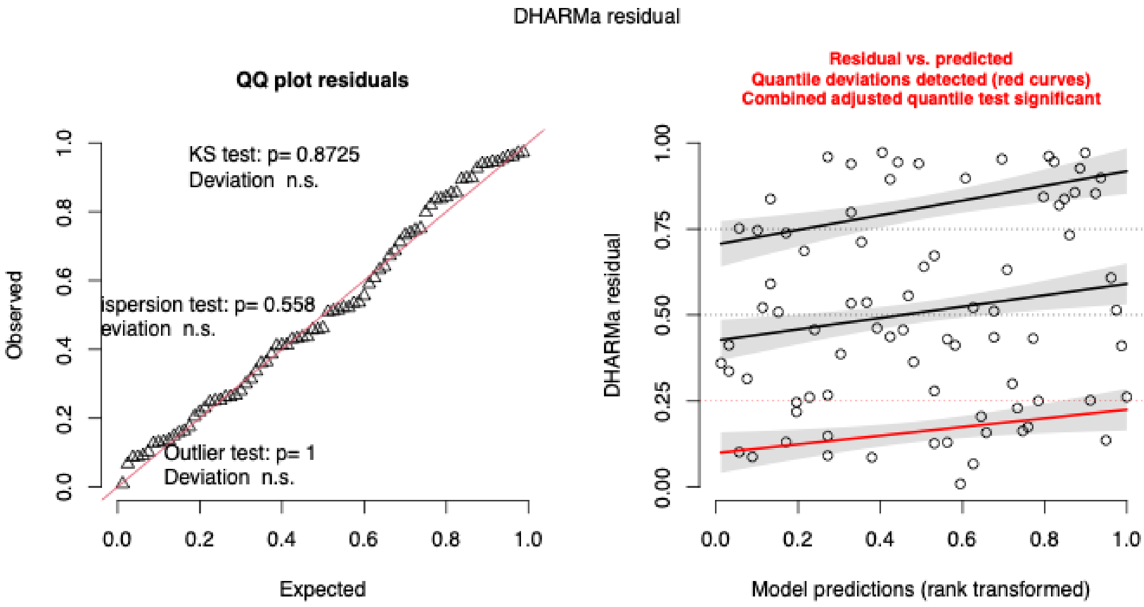

Figure S2. DHARMa residual check for the proportion of endemic species (PEnS) best model.

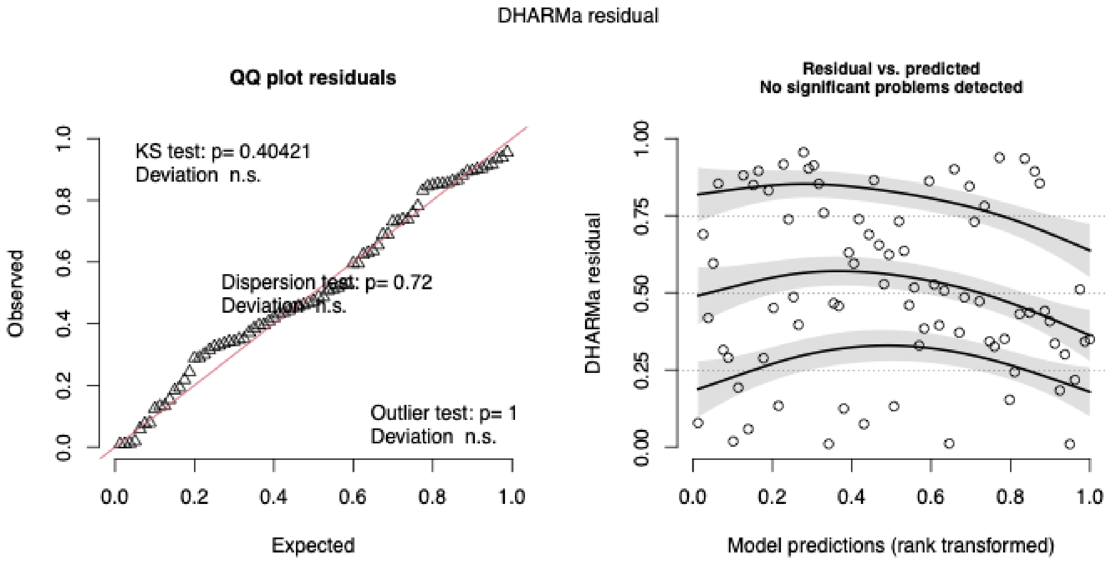

Figure S3. DHARMa residual check for the total salmonid species richness (NSSR) best model. Variance Inflation Factor was 1.04.

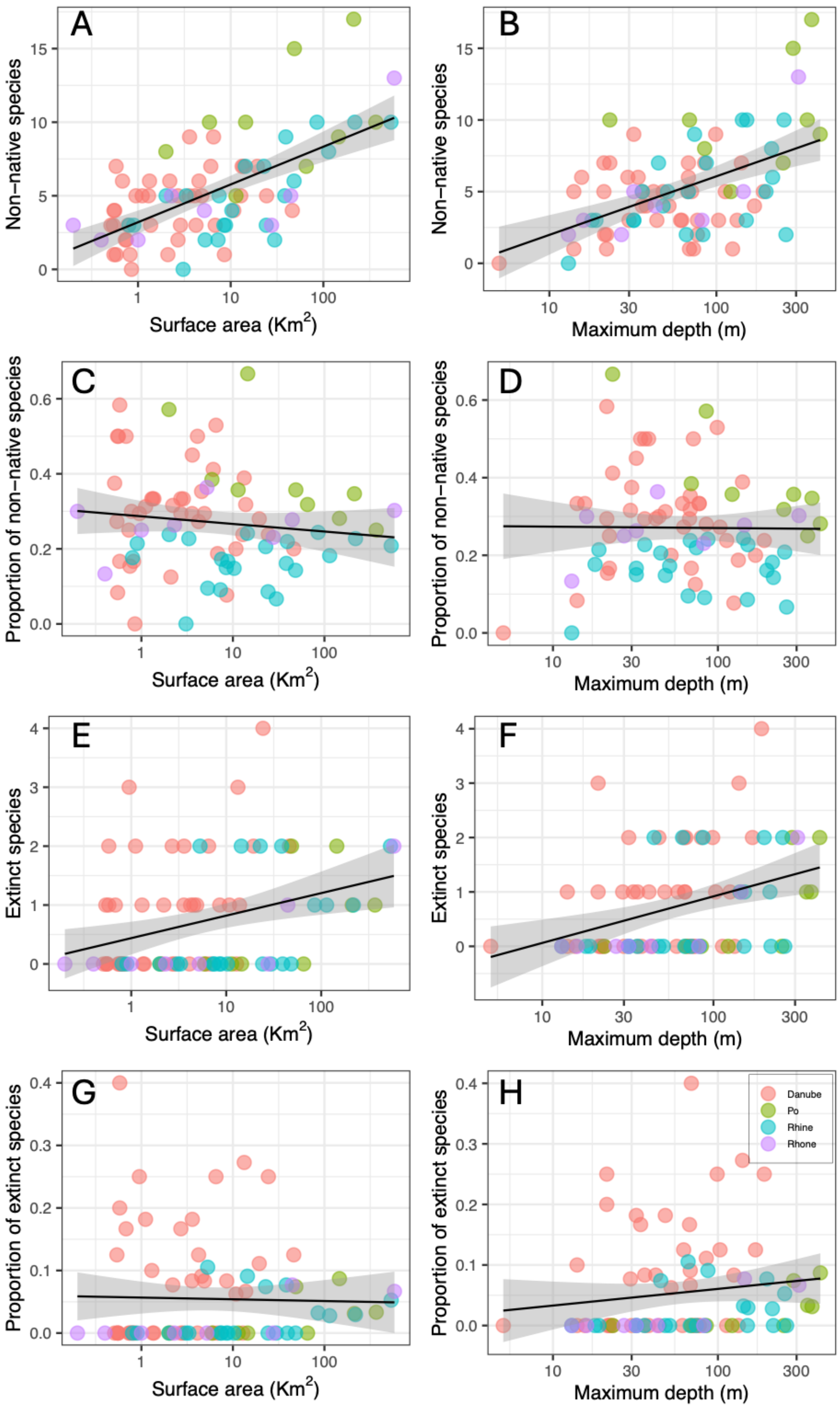

Figure S4. Relationships between lake features (surface area, km²; and maximum depth, m) and the numbers or proportions of non-native and extinct species in peri-Alpine lakes. Panels show: Richness of non-native species: (A) vs. lake area; (B) vs. maximum depth; Proportion of endemic species (C) vs. lake area; (D) vs. maximum depth. Richness of extinct species: (E) vs. lake area; (F) vs. maximum depth; (G) vs. lake area; (H) vs. maximum depth.

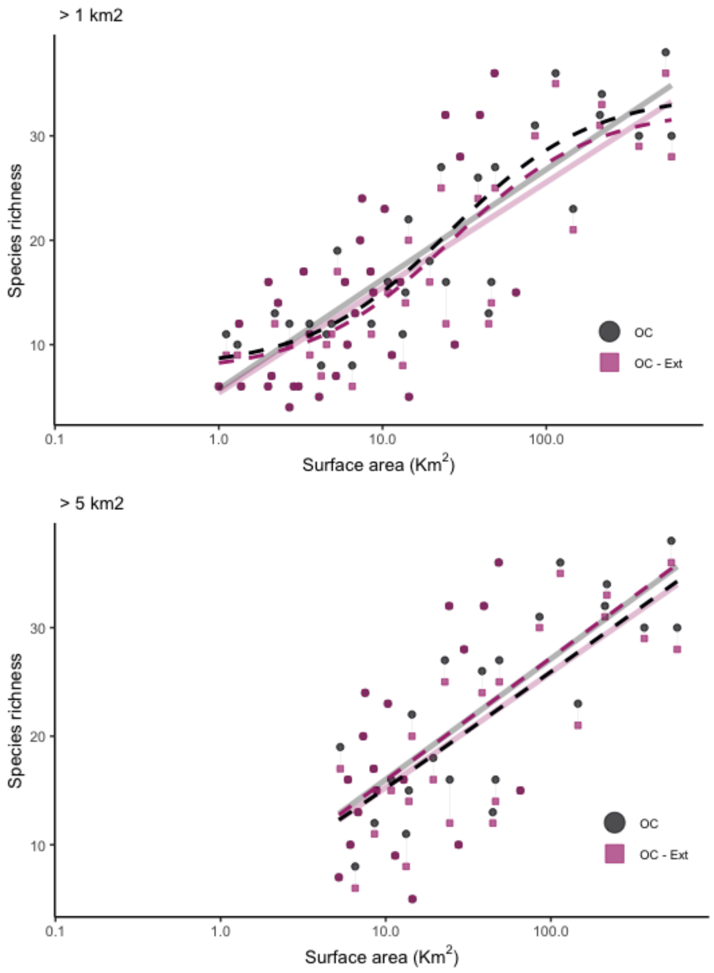

Figure S5. Relationship between lake surface area and the number of native fish species in the original community (OC) and after removing known human-driven extinctions (OC – Ext). Top panel: Lakes smaller than 1 km² (18 lakes) were excluded. In both datasets, the best-fitting model is the rational function: S=c+z·A1+d·AS=1+d·Ac+z·A. Bottom panel: Lakes smaller than 5 km² (39 lakes) were excluded. For the original community, the logarithmic function S=c+z·log(A)S=c+z·log(A) provides the best fit. In contrast, the dataset excluding extinct species is better represented by the Kobayashi function: S=c·log(1+A/z)S=c·log(1+A/z).

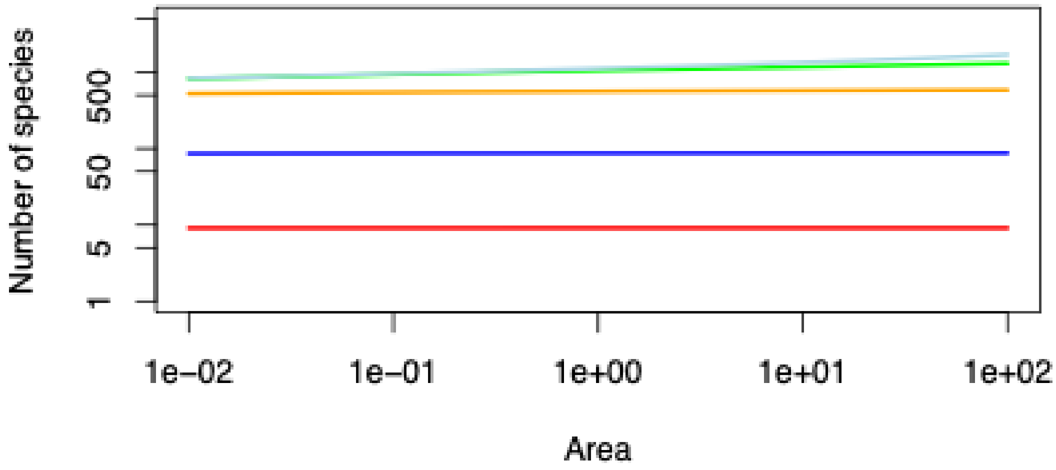

Figure S6. Island species-area relationship at various time slices (red: 0.001 My; blue: 0.01 My; orange: 0.1 My; green: 1 My; gray; 1000 My) according to the deterministic analog of the DAISIE model (Valente et al. 2017) with area-dependent cladogenesis and extinction estimated from avian data on 41 archipelagos worldwide (Valente et al. 2020). The parameters were obtained by fitting Valente et al.'s M14 model but without distance-dependence in colonization and anagenesis. The ISAR thus obtained represent what would be expected for birds on oceanic islands. The ISAR starts to deviate from being flat at a time scale of ~1 My. Hence, the clear ISAR pattern observed for the fish in the alpine lakes in this study is unexpected for such a young system (~0.01 My).

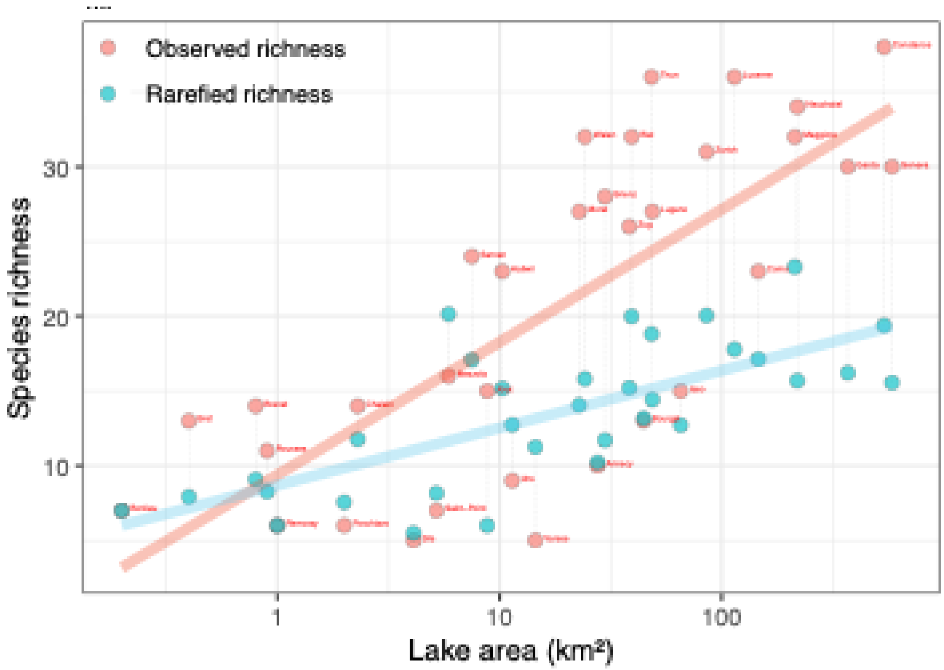

Figure S7. Island species–area relationship (ISAR) for a selected set of peri-Alpine lakes for which species abundance data from standardized sampling effort were available. Fitted curves are based on the power function. Red points represent the observed species richness, while blue points represent rarefied richness for the same lakes. If passive sampling were the main factor shaping the ISAR currently observed in the peri-Alpine lakes, rarefied richness should not exhibit a positive correlation with area. Therefore, we can rule out passive sampling as the primary driver of our empirical ISAR.

.

Table S1. Functions fitted to the Island Species–Area Relationship (ISAR), all four catchments combined, for the native fish richness in peri-Alpine lakes.

model	equation	c	d or T1	f or T2	z or z1	z2	z3	ML	AICc	AICcweight
**f_ratio**	S = (c + z * A)/(1 + d * A)	7.231	0.043	NA	1.443	NA	NA	-243.359	493.039	0.347
**f_ContOne**	$S = c_1 + (\log(A) \le T) * z_1 * \log(A) + (\log(A) > T) * (z_1 * T + z_2 * (\log(A) - T))$	8.604	1.411	NA	1.545	5.230	NA	-243.279	495.099	0.124
**f_p1**	S = c * A^z * exp(-d * A)	8.274	0.001	NA	0.278	NA	NA	-244.595	495.509	0.101
**f_asymp**	S = d - c * z^A	22.631	30.327	NA	0.964	NA	NA	-244.731	495.781	0.088
**f_ZslopeOne**	$S = c_1 + (\log(A) \le T) * z_1 * \log(A) + (\log(A) > T) * (z_1 * T + z_2 * (\log(A) - T))$	8.607	0.992	NA	0.000	5.234	NA	-245.000	496.320	0.067
**f_weibull3**	S = d * (1 - exp(-c * A^z))	0.204	44.848	NA	0.325	NA	NA	-245.489	497.299	0.041
**f_weibull4**	S = d * (1 - exp(-c * A^z))^f	0.000	32.651	0.116	2.340	NA	NA	-244.440	497.422	0.039
**f_heleg**	S = c/(f + A^(-z))	9.438	NA	0.135	0.330	NA	NA	-245.686	497.691	0.034
**f_mmf**	S = d/(1 + c * A^(-z))	7.417	70.009	NA	0.330	NA	NA	-245.686	497.691	0.034
**f_power**	S = c * A^z	8.679	NA	NA	0.241	NA	NA	-246.930	498.017	0.029
**f_epm1**	S = c * A^(z * A^(-d))	8.396	0.033	NA	0.290	NA	NA	-246.102	498.525	0.022
**f_gompertz**	S = d * exp(-exp(-z * (A - c)))	4.276	29.262	NA	0.059	NA	NA	-246.265	498.849	0.019
**f_betap**	S = d * (1 - (1 + (A/c)^z)^(-f))	2.16×10^{11}	44.863	995.667	0.325	NA	NA	-245.490	499.520	0.014
**f_epm2**	S = c * A^(z - d/A)	8.629	0.027	NA	0.243	NA	NA	-246.735	499.790	0.012
**f_p2**	S = c * A^z * exp(-d/A)	8.679	0.000	NA	0.241	NA	NA	-246.930	500.180	0.010
**f_powerR**	S = f + c * A^z	8.679	NA	0.000	0.241	NA	NA	-246.930	500.180	0.010
**f_ZslopeTwo**	$S = c_1 + (\log(A) \le T_1) * z_1 * \log(A) + (\log(A) > T_1) * (\log(A) \le T_2) * (z_1 * T_1 + z_2 * (\log(A) - T_1)) + (\log(A) > T_2) * (z_2 * (T_2 - T_1) + z_3 * (\log(A) - T_2))$	8.607	0.992	24.363	0.000	5.234	24.263	-245.000	500.822	0.007

**f_ContTwo**	$S = c_1 + (\log(A) \le T_1) * z_1 * \log(A) + (\log(A) > T_1) * (\log(A) \le T_2) * (z_1 * T_1 + z_2 * (\log(A) - T_1)) + (\log(A) > T_2) * (z_2 * (T_2 - T_1) + z_3 * (\log(A) - T_2))$	8.788	2.434	34.953	2.457	5.827	5.901	-245.807	504.782	0.001
**f_koba**	S = c * log(1 + A/z)	3.788	NA	NA	0.126	NA	NA	-250.854	505.866	0.001
**f_loga**	S = c + z * log(A)	8.721	NA	NA	3.436	NA	NA	-253.127	510.413	0.000
**f_RightZslopeOne**	$S = c_1 + (\log(A) \le T) * z_1 * \log(A) + (\log(A) > T) * z_1 * T$	8.721	13.562	NA	3.436	NA	NA	-253.127	512.575	0.000
**f_monod**	S = d/(1 + c * A^(-1))	2.019	22.878	NA	NA	NA	NA	-281.723	567.604	0.000
**f_linear**	S = c + z * A	12.097	NA	NA	0.073	NA	NA	-305.967	616.092	0.000
**f_negexpo**	S = d * (1 - exp(-z * A))	NA	20.164	NA	0.463	NA	NA	-307.614	619.387	0.000

Table S2. The best three functions fitted to the Island Species–Area Relationship (ISAR), after leaving out of the analysis one of the catchments (!catchment name), for the native fish richness in peri-Alpine lakes.

**Catchment**	**model**	**c**	**d or T1**	**f or T2**	**z or z1**	**z2**	**z3**	**ML**	**AICc**	**AICcweight**
**!Danube**	f_ratio	9.6097	0.0470	NA	1.5661	NA	NA	-136.2973	279.2803	0.1565
	f_ZslopeOne	10.2000	0.5218	NA	0.0000	4.3312	NA	-136.4957	279.6771	0.1284
	f_p1	10.5945	0.0005	NA	0.2268	NA	NA	-136.8350	280.3556	0.0914
**!Po**	f_ratio	7.1355	0.0430	NA	1.5452	NA	NA	-208.7632	423.8900	0.3930
	f_asymp	24.8241	32.4416	NA	0.9641	NA	NA	-209.7696	425.9028	0.1436
	f_ContOne	8.6614	1.4110	NA	1.5675	5.8095	NA	-208.7790	426.1735	0.1255
**!Rhine**	f_power	7.6505	NA	NA	0.2180	NA	NA	-150.9938	306.2098	0.1648
	f_powerR	3.4959	NA	4.3830	0.3364	NA	NA	-149.9669	306.3867	0.1509
	f_epm2	7.4966	0.0536	NA	0.2257	NA	NA	-150.2501	306.9530	0.1137
**!Rhone**	f_ratio	6.7630	0.0479	NA	1.6551	NA	NA	-214.3446	435.0474	0.2458
	f_asymp	23.5386	30.7359	NA	0.9581	NA	NA	-215.0180	436.3943	0.1253
	f_ContOne	8.2231	1.4110	NA	2.0169	5.5707	NA	-214.0066	436.6192	0.1120

Table S3. Functions fitted to the Island Species–Area Relationship (ISAR), all four catchments combined, for the fish richness in peri-Alpine lakes, including introduced or translocated species into the original native fish community.

model	equation	c	d or T1	f or T2	z or z1	z2	z3	ML	AICc	AICcweight
**f_p1**	**S = c * A^z * exp(-d * A)**	**11.6907645**	**0.00044342**	**NA**	**0.25455652**	**NA**	**NA**	**-252.37961**	**511.079228**	**0.16946789**
**f_power**	S = c * A^z	12.059935	NA	NA	0.23011556	NA	NA	-253.80867	511.775226	0.11966121
**f_ratio**	S = (c + z * A)/(1 + d * A)	10.6545703	0.03752273	NA	1.69297228	NA	NA	-252.73499	511.789989	0.11878115
**f_weibull4**	S = d * (1 - exp(-c * A^z))^f	8.13E-11	44.3449865	0.05722447	4.33467553	NA	NA	-251.9951	512.530747	0.08201513
**f_weibull3**	S = d * (1 - exp(-c * A^z))	0.16400757	77.6639268	NA	0.28268215	NA	NA	-253.17093	512.661863	0.07681085
**f_heleg**	S = c/(f + A^(-z))	12.9235625	NA	0.09690614	0.28307149	NA	NA	-253.25158	512.823166	0.07085919
**f_mmf**	S = d/(1 + c * A^(-z))	10.319277	133.361786	NA	0.28307148	NA	NA	-253.25158	512.823166	0.07085919
**f_epm1**	S = c * A^(z * A^(-d))	11.8532927	0.01922535	NA	0.25615918	NA	NA	-253.48313	513.286258	0.05621303
**f_epm2**	S = c * A^(z - d/A)	11.9960904	0.02336082	NA	0.23227768	NA	NA	-253.60053	513.52106	0.04998622
**f_ContOne**	$S = c_1 + (log(A) \le T) * z_1 * log(A) + (log(A) > T) * (z_1 * T + z_2 * (log(A) - T))$	12.2623582	2.14593128	NA	2.93017625	7.16464444	NA	-252.6121	513.764733	0.04425245
**f_p2**	S = c * A^z * exp(-d/A)	12.0599328	8.59E-15	NA	0.23011563	NA	NA	-253.80867	513.937331	0.04059366
**f_powerR**	S = f + c * A^z	12.0599327	NA	2.21E-13	0.23011563	NA	NA	-253.80867	513.937331	0.04059366
**f_betap**	S = d * (1 - (1 + (A/c)^z)^(-f))	2.41E+11	77.8415375	270.130106	0.28271212	NA	NA	-253.17156	514.883656	0.02529102
**f_ZslopeOne**	$S = c_1 + (log(A) \le T) * z_1 * log(A) + (log(A) > T) * (z_1 * T + z_2 * (log(A) - T))$	12.1428597	0.98683451	NA	0	6.60183653	NA	-254.71439	515.748784	0.01640991
**f_asymp**	S = d - c * z^A	29.8920057	41.2629403	NA	0.97015702	NA	NA	-255.12482	516.569635	0.01088581
**f_ContTwo**	$S = c_1 + (log(A) \le T_1) * z_1 * log(A) + (log(A) > T_1) * (log(A) \le T_2) * (z_1 * T_1 + z_2 * (log(A) - T_1)) + (log(A) > T_2) * (z_2 * (T_2 - T_1) + z_3 * (log(A) - T_2))$	12.2623497	2.14593128	19.0852381	2.93017933	7.16464945	1.69766738	-252.6121	518.39086	0.00437912

**f_ZslopeTwo**	$S = c_1 + (log(A) \le T_1) * z_1 * log(A) + (log(A) > T_1) * (log(A) \le T_2) * (z_1 * T_1 + z_2 * (log(A) - T_1)) + (log(A) > T_2) * (z_2 * (T_2 - T_1) + z_3 * (log(A) - T_2))$	12.1428572	0.98683413	27.0889192	0	6.60183523	10.8679362	-254.71439	520.250702	0.00172793
**f_gompertz**	S = d * exp(-exp(-z * (A - c)))	4.15153954	40.0559539	NA	0.04667456	NA	NA	-257.56446	521.448918	0.00094916
**f_koba**	S = c * log(1 + A/z)	4.79305099	NA	NA	0.09554699	NA	NA	-260.08611	524.330119	0.00022475
**f_loga**	S = c + z * log(A)	12.1087494	NA	NA	4.44677654	NA	NA	-262.13839	528.43468	2.89E-05
**f_RightZslopeOne**	$S = c_1 + (log(A) \le T) * z_1 * log(A) + (log(A) > T) * z_1 * T$	12.1087347	11.0146728	NA	4.44677905	NA	NA	-262.13839	530.596786	9.79E-06
**f_monod**	S = d/(1 + c * A^(-1))	1.67458938	29.6093559	NA	NA	NA	NA	-301.93299	608.023874	1.51E-22
**f_linear**	S = c + z * A	16.4502919	NA	NA	0.09490113	NA	NA	-320.875	645.907894	8.94E-31
**f_negexpo**	S = d * (1 - exp(-z * A))	NA	26.0250164	NA	0.57526444	NA	NA	-333.83532	671.828543	2.10E-36

Table S4. The best three functions fitted to the Island Species–Area Relationship (ISAR), after leaving out of the analysis one of the catchments (!catchment name), including introduced or translocated species into the original native fish community.

Catchment	model	c	d or T1	f or T2	z or z1	z2	z3	ML	AICc	AICcweight
**!Danube**	f_power	14.434	NA	NA	0.197	NA	NA	-137.297	278.928	0.134
	f_ratio	13.189	0.035	NA	1.609	NA	NA	-136.168	279.023	0.128
	f_p1	13.693	0.000	NA	0.226	NA	NA	-136.298	279.281	0.112
**!Po**	f_ratio	10.482	0.038	NA	1.746	NA	NA	-219.165	444.693	0.185
	f_p1	11.633	0.000	NA	0.261	NA	NA	-219.452	445.268	0.139
	f_ContOne	12.043	1.440	NA	2.117	6.853	NA	-218.385	445.384	0.131
**!Rhine**	f_power	11.255	NA	NA	0.214	NA	NA	-168.846	341.914	0.209
	f_powerR	6.553	NA	4.919	0.293	NA	NA	-168.219	342.891	0.128
	f_epm2	11.108	0.036	NA	0.219	NA	NA	-168.386	343.226	0.108
**!Rhone**	f_p1	11.427	0.001	NA	0.275	NA	NA	-221.459	449.276	0.163
	f_ratio	10.187	0.043	NA	1.968	NA	NA	-221.564	449.486	0.147
	f_weibull3	0.198	63.338	NA	0.323	NA	NA	-222.050	450.458	0.090

Table s5. Functions fitted to the Island Species–Area Relationship (ISAR), all four catchments combined, for the fish richness in peri-Alpine lakes, removing the extinct or extirpated species from the original native fish community.

model	equation	c	d or T1	f or T2	z or z1	z2	z3	ML	AICc	AICcweight
**f_ratio**	**S = (c + z * A)/(1 + d * A)**	**6.83922209**	**0.04235973**	**NA**	**1.36282894**	**NA**	**NA**	**-246.21626**	**498.752516**	**0.38597509**
**f_p1**	S = c * A^z * exp(-d * A)	7.81162286	0.00068651	NA	0.28100016	NA	NA	-247.4281	501.176202	0.11488474
**f_asymp**	S = d - c * z^A	21.7866872	29.0813196	NA	0.96485503	NA	NA	-247.47442	501.268842	0.10968468
**f_ZslopeOne**	$S = c_1 + (\log(A) \le T) * z_1 * \log(A) + (\log(A) > T) * (z_1 * T + z_2 * (\log(A) - T))$	8.13743886	0.99325178	NA	0	5.01486143	NA	-247.83511	501.990214	0.07647192
**f_weibull4**	S = d * (1 - exp(-c * A^z))^f	2.30E-06	31.1171125	0.10622217	2.57749418	NA	NA	-247.25328	503.047108	0.04508171
**f_weibull3**	S = d * (1 - exp(-c * A^z))	0.20262492	42.6254273	NA	0.32816466	NA	NA	-248.41566	503.151325	0.04279273
**f_heleg**	S = c/(f + A^(-z))	8.91307451	NA	0.13347426	0.33217846	NA	NA	-248.62056	503.56113	0.03486438
**f_mmf**	S = d/(1 + c * A^(-z))	7.49209114	66.7775725	NA	0.33217829	NA	NA	-248.62056	503.56113	0.03486438
**f_power**	S = c * A^z	8.21284443	NA	NA	0.2427169	NA	NA	-249.84235	503.842587	0.03028756
**f_gompertz**	S = d * exp(-exp(-z * (A - c)))	4.52772974	28.1008522	NA	0.05754752	NA	NA	-249.02499	504.369972	0.0232672
**f_epm1**	S = c * A^(z * A^(-d))	7.94230007	0.03284756	NA	0.29212116	NA	NA	-249.04841	504.416828	0.02272843
**f_betap**	S = d * (1 - (1 + (A/c)^z)^(-f))	2.35E+11	42.636599	1093.49222	0.3282099	NA	NA	-248.41604	505.372623	0.01409357
**f_epm2**	S = c * A^(z - d/A)	8.15332746	0.03286958	NA	0.24565804	NA	NA	-249.55446	505.428912	0.01370245
**f_ContTwo**	$S = c_1 + (\log(A) \le T_1) * z_1 * \log(A) + (\log(A) > T_1) * (\log(A) \le T_2) * (z_1 * T_1 + z_2 * (\log(A) - T_1)) + (\log(A) > T_2) * (z_2 * (T_2 - T_1) + z_3 * (\log(A) - T_2))$	8.14531366	1.41098697	99.8955189	1.43849938	5.03277175	11.6283069	-246.2147	505.596063	0.01260381
**f_p2**	S = c * A^z * exp(-d/A)	8.21284195	1.83E-14	NA	0.242717	NA	NA	-249.84235	506.004692	0.0102747
**f_powerR**	S = f + c * A^z	8.21284201	NA	5.88E-14	0.24271699	NA	NA	-249.84235	506.004692	0.0102747
**f_ContOne**	$S = c_1 + (\log(A) \le T) * z_1 * \log(A) + (\log(A) > T) * (z_1 * T + z_2 * (\log(A) - T))$	8.32935711	2.43361336	NA	2.32816138	5.60506361	NA	-248.81768	506.175909	0.0094317
**f_ZslopeTwo**	$S = c_1 + (\log(A) \le T_1) * z_1 * \log(A) + (\log(A) > T_1) * (\log(A) \le T_2) * (z_1 * T_1 + z_2 * (\log(A) - T_1)) + (\log(A) > T_2) * (z_2 * (T_2 - T_1) + z_3 * (\log(A) - T_2))$	8.1374349	0.99325177	25.6023174	0	5.01486658	11.6022967	-247.83511	506.492132	0.00805236
**f_koba**	S = c * log(1 + A/z)	3.62739667	NA	NA	0.12973397	NA	NA	-253.78148	511.720851	0.00058955
**f_loga**	S = c + z * log(A)	8.27668591	NA	NA	3.27356314	NA	NA	-256.14428	516.446454	5.55E-05

**f_RightZslopeOne**	$S = c_1 + (log(A) \le T) * z_1 * log(A) + (log(A) > T) * z_1 * T$	8.27669231	10.164506	NA	3.27356467	NA	NA	-256.14428	518.608559	1.88E-05
**f_monod**	S = d/(1 + c * A^(-1))	2.06464705	21.8613964	NA	NA	NA	NA	-283.42576	571.009405	7.87E-17
**f_linear**	S = c + z * A	11.4704726	NA	NA	0.06992443	NA	NA	-306.85715	617.872204	5.25E-27
**f_negexpo**	S = d * (1 - exp(-z * A))	NA	19.2733466	NA	0.45089321	NA	NA	-308.20996	620.577823	1.36E-27

Table S6. The best three functions fitted to the Island Species–Area Relationship (ISAR), after leaving out of the analysis one of the catchments (!catchment name), removing the extinct or extirpated species from the original native fish community.

Catchment	model	c	d or T1	f or T2	z or z1	z2	z3	ML	AICc	AICcweight
**!Danube**	f_ratio	9.612	0.049	NA	1.549	NA	NA	-135.564	277.814	0.151
	f_ZslopeOne	10.200	0.497	NA	0.000	4.049	NA	-135.870	278.425	0.112
	f_p1	10.557	0.001	NA	0.220	NA	NA	-136.072	278.829	0.091
**!Po**	f_ratio	6.748	0.042	NA	1.444	NA	NA	-213.069	432.502	0.394
	f_asymp	24.026	31.241	NA	0.965	NA	NA	-213.874	434.112	0.176
	f_ContOne	8.185	1.411	NA	1.432	5.604	NA	-213.214	435.043	0.111
**!Rhine**	f_powerR	2.812	NA	4.554	0.361	NA	NA	-147.227	300.907	0.169
	f_power	7.107	NA	NA	0.219	NA	NA	-148.607	301.436	0.130
	f_epm2	6.932	0.064	NA	0.228	NA	NA	-147.572	301.596	0.120
**!Rhone**	f_ratio	6.307	0.047	NA	1.571	NA	NA	-217.413	441.185	0.245
	f_asymp	22.789	29.529	NA	0.959	NA	NA	-218.137	442.632	0.119
	f_p1	7.482	0.001	NA	0.308	NA	NA	-218.214	442.787	0.110

Table S7. Functions fitted to the Island Species–Area Relationship (ISAR), all catchment combined, for the fish richness in *peri-Alpine* lakes, including introduced or translocated species into and removing the extinct or extirpated species from the original native fish community.

model	equation	c	d or T1	f or T2	z or z1	z2	z3	ML	AICc	AICcweight
**f_p1**	**S = c * A^z * exp(-d * A)**	**11.1979769**	**0.00045664**	**NA**	**0.25659364**	**NA**	**NA**	**-252.17693**	**510.673865**	**0.17302702**
**f_ratio**	S = (c + z * A)/(1 + d * A)	10.2400437	0.03676791	NA	1.60817674	NA	NA	-252.51226	511.34451	0.1237329
**f_power**	S = c * A^z	11.5638592	NA	NA	0.23138357	NA	NA	-253.63673	511.431363	0.11847463
**f_weibull4**	S = d * (1 - exp(-c * A^z))^f	7.27E-11	42.8201061	0.05728971	4.36006619	NA	NA	-251.72696	511.994457	0.08940277
**f_weibull3**	S = d * (1 - exp(-c * A^z))	0.16171284	75.4354712	NA	0.28367793	NA	NA	-253.02451	512.369019	0.07413375
**f_heleg**	S = c/(f + A^(-z))	12.3685101	NA	0.09473652	0.28362739	NA	NA	-253.10599	512.531983	0.06833272
**f_mmf**	S = d/(1 + c * A^(-z))	10.5555768	130.556793	NA	0.28362743	NA	NA	-253.10599	512.531983	0.06833272
**f_epm1**	S = c * A^(z * A^(-d))	11.3703153	0.01861243	NA	0.25672203	NA	NA	-253.33904	512.998083	0.05412731
**f_epm2**	S = c * A^(z - d/A)	11.4889786	0.02826978	NA	0.23401485	NA	NA	-253.33926	512.998519	0.05411551
**f_p2**	S = c * A^z * exp(-d/A)	11.5638601	7.72E-15	NA	0.23138352	NA	NA	-253.63673	513.593468	0.04019112
**f_powerR**	S = f + c * A^z	11.5638599	NA	1.22E-13	0.23138352	NA	NA	-253.63673	513.593468	0.04019112
**f_ContOne**	$S = c_1 + (\log(A) \le T) * z_1 * \log(A) + (\log(A) > T) * (z_1 * T + z_2 * (\log(A) - T))$	11.7728381	2.14593129	NA	2.81799607	6.93636816	NA	-252.6519	513.844341	0.03545306
**f_betap**	S = d * (1 - (1 + (A/c)^z)^(-f))	2.6542E+11	75.6211424	280.891673	0.28368717	NA	NA	-253.02512	514.590789	0.02440983
**f_ZslopeOne**	$S = c_1 + (\log(A) \le T) * z_1 * \log(A) + (\log(A) > T) * (z_1 * T + z_2 * (\log(A) - T))$	11.6560588	0.99325178	NA	0	6.39592608	NA	-254.55387	515.427741	0.01606286
**f_asymp**	S = d - c * z^A	29.1162972	40.0599271	NA	0.97095007	NA	NA	-254.72853	515.777068	0.01348862
**f_ContTwo**	$S = c_1 + (\log(A) \le T_1) * z_1 * \log(A) + (\log(A) > T_1) * (\log(A) \le T_2) * (z_1 * T_1 + z_2 * (\log(A) - T_1)) + (\log(A) > T_2) * (z_2 * (T_2 - T_1) + z_3 * (\log(A) - T_2))$	11.8021844	2.43361336	8.73005842	2.98179389	7.31455431	1.97322912	-252.69637	518.559406	0.00335576
**f_ZslopeTwo**	$S = c_1 + (\log(A) \le T_1) * z_1 * \log(A) + (\log(A) > T_1) * (\log(A) \le T_2) * (z_1 * T_1 + z_2 * (\log(A) - T_1)) + (\log(A) > T_2) * (z_2 * (T_2 - T_1) + z_3 * (\log(A) - T_2))$	11.6560524	0.99325177	25.2406051	0	6.39597354	10.8330015	-254.55387	519.929659	0.00169139
**f_gompertz**	S = d * exp(-exp(-z * (A - c)))	4.45643877	38.9711497	NA	0.04504151	NA	NA	-257.12095	520.561891	0.00123298
**f_koba**	S = c * log(1 + A/z)	4.63513811	NA	NA	0.09771869	NA	NA	-259.97106	524.100005	0.00021022

**f_loga**	S = c + z * log(A)	11.6349769	NA	NA	4.28653305	NA	NA	-262.09372	528.345345	2.52E-05
**f_RightZslopeOne**	S = c_1 + (log(A) ≤ T) * z_1 * log(A) + (log(A) > T) * z_1 * T	11.6349894	7.20830043	NA	4.28652913	NA	NA	-262.09372	530.50745	8.54E-06
**f_monod**	S = d/(1 + c * A^(-1))	1.69929943	28.5779846	NA	NA	NA	NA	-300.69276	605.543409	4.34E-22
**f_linear**	S = c + z * A	15.7931963	NA	NA	0.0921925	NA	NA	-318.751	641.659902	6.24E-30
**f_negexpo**	S = d * (1 - exp(-z * A))	NA	25.1158183	NA	0.566303	NA	NA	-331.5578	667.273496	1.71E-35

Table S8. The best three functions fitted to the Island Species–Area Relationship (ISAR), after leaving out of the analysis one of the catchments (!catchment name), including introduced or translocated species into and removing the extinct or extirpated species

Catchment	model	c	d or T1	f or T2	z or z1	z2	z3	ML	AICc	AICcweight
**!Danube**	f_power	14.391	NA	NA	0.191	NA	NA	-136.484	277.301	0.133
	f_ratio	13.196	0.036	NA	1.577	NA	NA	-135.348	277.381	0.127
	f_p1	13.656	0.000	NA	0.220	NA	NA	-135.489	277.663	0.111
**!Po**	f_ratio	10.071	0.036	NA	1.639	NA	NA	-219.523	445.409	0.209
	f_p1	11.118	0.000	NA	0.263	NA	NA	-219.964	446.292	0.135
	f_ContOne	11.537	1.440	NA	1.986	6.643	NA	-218.981	446.577	0.117
**!Rhine**	f_power	10.676	NA	NA	0.215	NA	NA	-165.662	335.546	0.185
	f_powerR	5.628	NA	5.299	0.310	NA	NA	-164.803	336.058	0.143
	f_epm2	10.504	0.044	NA	0.221	NA	NA	-164.999	336.451	0.118
**!Rhone**	f_p1	10.875	0.001	NA	0.279	NA	NA	-221.471	449.300	0.170
	f_ratio	9.705	0.042	NA	1.879	NA	NA	-221.840	450.037	0.118
	f_weibull3	0.194	61.294	NA	0.327	NA	NA	-222.087	450.533	0.092
