# Supplementary material for: Recent and Rapid Assembly of an Island Species–Area Relationship Threatened by Human Disturbance
Source: Ecol Lett. 2025 Oct 5;28(10):e70222. doi: 10.1111/ele.70222 (PMC12498083; doi:10.1111/ele.70222)
Supplement: Supplementary file 4 — Data S4: ele70222‐sup‐0004‐Supinfo4.html. [file ELE-28-0-s002.html]

Generalized linear mixed models for island species–area relationship of the peri-Alpine lakes


# Generalized linear mixed models for island species–area relationship of the peri-Alpine lakes

#### Luiz Jardim de Queiroz

#### 2025-04-15

```
packages <- c(
  "glmmTMB",
  "AICcmodavg",
  "MuMIn",
  "car",
  "dplyr",
  "scales",
  "DHARMa",
  "corrplot",
  "ggplot2",
  "ggeffects",
  "sjPlot",
  "cowplot",
  "knitr"
)

# Install missing packages
installed <- rownames(installed.packages())
for (pkg in packages) {
  if (!(pkg %in% installed)) {
    install.packages(pkg, dependencies = TRUE)
  }
}

# Load packages
lapply(packages, library, character.only = TRUE)
```

```
## Warning in checkDepPackageVersion(dep_pkg = "TMB"): Package version inconsistency detected.
## glmmTMB was built with TMB version 1.9.10
## Current TMB version is 1.9.11
## Please re-install glmmTMB from source or restore original 'TMB' package (see '?reinstalling' for more information)
```

```
## 
## Attaching package: 'MuMIn'
```

```
## The following objects are masked from 'package:AICcmodavg':
## 
##     AICc, DIC, importance
```

```
## Loading required package: carData
```

```
## 
## Attaching package: 'dplyr'
```

```
## The following object is masked from 'package:car':
## 
##     recode
```

```
## The following objects are masked from 'package:stats':
## 
##     filter, lag
```

```
## The following objects are masked from 'package:base':
## 
##     intersect, setdiff, setequal, union
```

```
## This is DHARMa 0.4.6. For overview type '?DHARMa'. For recent changes, type news(package = 'DHARMa')
```

```
## corrplot 0.92 loaded
```

```
## Learn more about sjPlot with 'browseVignettes("sjPlot")'.
```

```
## 
## Attaching package: 'cowplot'
```

```
## The following objects are masked from 'package:sjPlot':
## 
##     plot_grid, save_plot
```

```
## The following object is masked from 'package:ggeffects':
## 
##     get_title
```

```
## [[1]]
## [1] "glmmTMB"   "stats"     "graphics"  "grDevices" "utils"     "datasets" 
## [7] "methods"   "base"     
## 
## [[2]]
## [1] "AICcmodavg" "glmmTMB"    "stats"      "graphics"   "grDevices" 
## [6] "utils"      "datasets"   "methods"    "base"      
## 
## [[3]]
##  [1] "MuMIn"      "AICcmodavg" "glmmTMB"    "stats"      "graphics"  
##  [6] "grDevices"  "utils"      "datasets"   "methods"    "base"      
## 
## [[4]]
##  [1] "car"        "carData"    "MuMIn"      "AICcmodavg" "glmmTMB"   
##  [6] "stats"      "graphics"   "grDevices"  "utils"      "datasets"  
## [11] "methods"    "base"      
## 
## [[5]]
##  [1] "dplyr"      "car"        "carData"    "MuMIn"      "AICcmodavg"
##  [6] "glmmTMB"    "stats"      "graphics"   "grDevices"  "utils"     
## [11] "datasets"   "methods"    "base"      
## 
## [[6]]
##  [1] "scales"     "dplyr"      "car"        "carData"    "MuMIn"     
##  [6] "AICcmodavg" "glmmTMB"    "stats"      "graphics"   "grDevices" 
## [11] "utils"      "datasets"   "methods"    "base"      
## 
## [[7]]
##  [1] "DHARMa"     "scales"     "dplyr"      "car"        "carData"   
##  [6] "MuMIn"      "AICcmodavg" "glmmTMB"    "stats"      "graphics"  
## [11] "grDevices"  "utils"      "datasets"   "methods"    "base"      
## 
## [[8]]
##  [1] "corrplot"   "DHARMa"     "scales"     "dplyr"      "car"       
##  [6] "carData"    "MuMIn"      "AICcmodavg" "glmmTMB"    "stats"     
## [11] "graphics"   "grDevices"  "utils"      "datasets"   "methods"   
## [16] "base"      
## 
## [[9]]
##  [1] "ggplot2"    "corrplot"   "DHARMa"     "scales"     "dplyr"     
##  [6] "car"        "carData"    "MuMIn"      "AICcmodavg" "glmmTMB"   
## [11] "stats"      "graphics"   "grDevices"  "utils"      "datasets"  
## [16] "methods"    "base"      
## 
## [[10]]
##  [1] "ggeffects"  "ggplot2"    "corrplot"   "DHARMa"     "scales"    
##  [6] "dplyr"      "car"        "carData"    "MuMIn"      "AICcmodavg"
## [11] "glmmTMB"    "stats"      "graphics"   "grDevices"  "utils"     
## [16] "datasets"   "methods"    "base"      
## 
## [[11]]
##  [1] "sjPlot"     "ggeffects"  "ggplot2"    "corrplot"   "DHARMa"    
##  [6] "scales"     "dplyr"      "car"        "carData"    "MuMIn"     
## [11] "AICcmodavg" "glmmTMB"    "stats"      "graphics"   "grDevices" 
## [16] "utils"      "datasets"   "methods"    "base"      
## 
## [[12]]
##  [1] "cowplot"    "sjPlot"     "ggeffects"  "ggplot2"    "corrplot"  
##  [6] "DHARMa"     "scales"     "dplyr"      "car"        "carData"   
## [11] "MuMIn"      "AICcmodavg" "glmmTMB"    "stats"      "graphics"  
## [16] "grDevices"  "utils"      "datasets"   "methods"    "base"      
## 
## [[13]]
##  [1] "knitr"      "cowplot"    "sjPlot"     "ggeffects"  "ggplot2"   
##  [6] "corrplot"   "DHARMa"     "scales"     "dplyr"      "car"       
## [11] "carData"    "MuMIn"      "AICcmodavg" "glmmTMB"    "stats"     
## [16] "graphics"   "grDevices"  "utils"      "datasets"   "methods"   
## [21] "base"
```

## GLMM analyses

This is the code to run the GLMM analyses on the fish species richnes
of the peri-Alpine lakes. Jardim de Queiroz et al. *Recent and rapid
assembly of an island species–area relationship in postglacial lakes
threatened by human disturbance*.

The GLMM analyses are intended to test the effect of lake features on
three dimensions of richness:

1. **Total native species richness**
2. **Total native salmonid species richness**
3. **Endemism proportion**

The lake features and richness data are here.

```
data <- read.csv("Supporting_information_S2.csv")
data$Catchment <- as.factor(data$Catchment)
```

## Multicollinearity assessement

We used Variance Inflation Factor (VIF) to check the degree of
multicollinearity. We first run a linear model containing of explanatory
variables.

```
full.lm.model <- lm(D1.Total_native_Richness ~ . 
                        - Lake
                        - Catchment
                        - D1.Native.Salmonidae.richness
                        - D2.Total.richness
                        - D3.Total_Native_extant.richness
                        - D6.Total.Extant.richness
                        - D5.Endemic
                        - Endemism_proportion
                        - Lat
                        - Long
                    ,
                        data = data)

car::vif(full.lm.model)
```

```
##                   Elevation_m_pl                 Surface_area_km2 
##                        40.069988                       119.847497 
##                  Maximum_depth_m                  Average_depth_m 
##                        70.770342                        42.431246 
##                Volume_10.6_m3_GL           Shore_length_hydrolake 
##                        71.067639                        61.337903 
##                Dis_avg_hydrolake              Wshd_area_hydrolake 
##                       167.058005                       117.121937 
## avg_surface_temp_domischetal2015       avg_air_temperature_chelsa 
##                         8.131349                        66.016354 
##            air_temp_range_chelsa  surf_temp_range_domischetal2015 
##                         9.796031                        13.128573 
##          distance_to_river_mouth             distance_to_refugium 
##                        38.497580                        17.609344 
##                   mean_steepness                  range_steepness 
##                         3.628155                        12.077294
```

Then we removed variables showing high values of VIF and re-check for
multicollinearity:

```
reduced.lm.model <- lm(D1.Total_native_Richness ~ . 
                        - Lake
                        - Catchment
                        - D1.Native.Salmonidae.richness
                        - D2.Total.richness
                        - D3.Total_Native_extant.richness
                        - D6.Total.Extant.richness
                        - D5.Endemic
                        - Endemism_proportion
                        - Lat
                        - Long
                       
                       # Removing explanatory variables to improve VIF:
                       - Elevation_m_pl
                       - Volume_10.6_m3_GL
                       - Average_depth_m
                       - Shore_length_hydrolake
                       - Dis_avg_hydrolake
                       - Wshd_area_hydrolake
                       - avg_air_temperature_chelsa
                       - air_temp_range_chelsa
                       - distance_to_river_mouth
                       ,
                        data = data)

car::vif(reduced.lm.model)
```

```
##                 Surface_area_km2                  Maximum_depth_m 
##                         1.939047                         2.161027 
## avg_surface_temp_domischetal2015  surf_temp_range_domischetal2015 
##                         2.255684                         1.858988 
##             distance_to_refugium                   mean_steepness 
##                         2.396083                         2.067035 
##                  range_steepness 
##                         3.365685
```

Making a list of the variables to include in the model and
creating/plotting a correlation matrix to inspect pairwise correlation
coeficients.

```
var.to.model <- rownames(summary(reduced.lm.model)$coefficients)[-1]

correlation_matrix <- cor(data[,var.to.model], use = "pairwise.complete.obs")


corrplot::corrplot(correlation_matrix, 
                   method = "circle",
                   addCoef.col = "black",
                   number.digits = 1,
                   tl.cex = 0.5,
                   number.cex = 0.5,
                   col = colorRampPalette(c("red", "white", "red"))(n = 100)
)
```

```
## Warning in ind1:ind2: numerical expression has 2 elements: only the first used
```

With the final list of variables, let’s create a list of all possible
combinations of variables. That will be useful for the model selection
step.

```
generate_combinations <- function(vars) {
  result <- list()
  for (i in 1:length(vars)) {
    combinations <- combn(vars, i)
    for (j in 1:ncol(combinations)) {
      result[[length(result) + 1]] <- combinations[, j]
    }
  }
  return(result)
}

# Generate all combinations of variables for the model
combinations_list <- generate_combinations(var.to.model)
```

## Running GLMM on Endemism Proportion (PEnS)

We used the R package *glmmTMB*

```
citation("glmmTMB")
```

```
## To cite glmmTMB in publications use:
## 
##   Mollie E. Brooks, Kasper Kristensen, Koen J. van Benthem, Arni
##   Magnusson, Casper W. Berg, Anders Nielsen, Hans J. Skaug, Martin
##   Maechler and Benjamin M. Bolker (2017). glmmTMB Balances Speed and
##   Flexibility Among Packages for Zero-inflated Generalized Linear Mixed
##   Modeling. The R Journal, 9(2), 378-400. doi: 10.32614/RJ-2017-066.
## 
## A BibTeX entry for LaTeX users is
## 
##   @Article{,
##     author = {Mollie E. Brooks and Kasper Kristensen and Koen J. {van Benthem} and Arni Magnusson and Casper W. Berg and Anders Nielsen and Hans J. Skaug and Martin Maechler and Benjamin M. Bolker},
##     title = {{glmmTMB} Balances Speed and Flexibility Among Packages for Zero-inflated Generalized Linear Mixed Modeling},
##     year = {2017},
##     journal = {The R Journal},
##     doi = {10.32614/RJ-2017-066},
##     pages = {378--400},
##     volume = {9},
##     number = {2},
##   }
```

For variable combination generated in the previous step, we fitted a
GLMM with or without random variable (*Catchment*), and with or
without accounting for *zero inflation*.

Continuous explanatory variables were first log-transformed and
rescaled to vary in range between 0 and 1.

```
data$Catchment <- as.factor(data$Catchment)
random_var <- 'Catchment'
response_var <- "Endemism_proportion"

PEnS_model_list_no_random <- list()
PEnS_model_list_no_random_notation <- list()

library(glmmTMB)


   #####################################################################
  ### PEnS MODELS WITHOUT THE RANDOM VARIABLE AND NO ZERO INFLATION ### 
######################################################################

for (i in seq_along(combinations_list)) {
  
  vars <- combinations_list[[i]]
  
  transformed_vars <- sapply(vars, function(var) {
      paste0("scales::rescale(log1p(", var, "))")
    }
  )
  
  formula_str <- paste0(response_var, "~", paste(transformed_vars, collapse = " + "))
  formula_obj <- as.formula(formula_str)
  
  PEnS_model_list_no_random[[i]] <- glmmTMB::glmmTMB(formula = formula_obj, 
                                                ziformula = ~0, 
                                                family = ordbeta(link = "logit"), 
                                                REML = F, 
                                                data = data)
  
  PEnS_model_list_no_random_notation[[i]] <- formula_str
}

   ###################################################################
  ### PEnS MODELS WITHOUT THE RANDOM VARIABLE AND  ZERO INFLATION ### 
####################################################################

PEnS_model_list_no_random_zi <- list()
PEnS_model_list_no_random_notation_zi <- list()

for (i in seq_along(combinations_list)) {
  
  vars <- combinations_list[[i]]
  
  transformed_vars <- sapply(vars, function(var) {
      paste0("scales::rescale(log1p(", var, "))")
    }
  )
  
  formula_str <- paste0(response_var, "~", paste(transformed_vars, collapse = " + "))
  formula_obj <- as.formula(formula_str)
 
  PEnS_model_list_no_random_zi[[i]] <- glmmTMB(formula = formula_obj, 
                                          ziformula = ~1, 
                                          family = ordbeta(link = "logit"), 
                                          REML = F, 
                                          data = data)
  
  PEnS_model_list_no_random_notation_zi[[i]] <- formula_str
}

   ###################################################################
  ### PEnS MODELS WITH THE RANDOM VARIABLE AND NO ZERO INFLATION ### 
####################################################################

PEnS_model_list_with_random <- list()
PEnS_model_list_with_random_notation <- list()

for (i in seq_along(combinations_list)) {
  
  vars <- combinations_list[[i]]
  
  transformed_vars <- sapply(vars, function(var) {
      paste0("scales::rescale(log1p(", var, "))")
    }
  )
  
  formula_str <- paste0(response_var, "~", paste(transformed_vars, collapse = " + "), " + (1|Catchment)")
  formula_obj <- as.formula(formula_str)
  
  PEnS_model_list_with_random[[i]] <- glmmTMB(formula = formula_obj, 
                                              ziformula = ~0, 
                                              family = ordbeta(link = "logit"), 
                                              REML=F, 
                                              data = data)
  
  PEnS_model_list_with_random_notation[[i]] <- formula_str
}

   ###############################################################
  ### PEnS MODELS WITH THE RANDOM VARIABLE AND ZERO INFLATION ### 
################################################################

PEnS_model_list_with_random_zi <- list()
PEnS_model_list_with_random_notation_zi <- list()

for (i in seq_along(combinations_list)) {
  
  vars <- combinations_list[[i]]
  
  transformed_vars <- sapply(vars, function(var) {
      paste0("scales::rescale(log1p(", var, "))")
    }
  )
  
  formula_str <- paste0(response_var, "~", paste(transformed_vars, collapse = " + "), " + (1|Catchment)")
  formula_obj <- as.formula(formula_str)
  
  PEnS_model_list_with_random_zi[[i]] <- glmmTMB(formula = formula_obj, 
                                                 ziformula = ~ 1,
                                                 family = ordbeta(link = "logit"),
                                                 REML=F, 
                                                 data = data)
  
  PEnS_model_list_with_random_notation_zi[[i]] <- formula_str
}
```

# Summarizing the models for model selection

First, let’s summarize the results from the models for *without
random variable* and *without zero inflation*

```
PEnS_summary.models <- as.data.frame(matrix(data=NA, nrow=1, ncol=8))

names(PEnS_summary.models) <- c("formula", 
                                "AIC", 
                                "AICc", 
                                "BIC", 
                                "R2.mar.mumin", 
                                "R2.cond.mumin", 
                                "random_var",
                                "zi")

PEnS_summary.models.no.random <- list()
PEnS_summary.models.no.random_zi <- list()
PEnS_summary.models.with.random <- list()
PEnS_summary.models.with.random_zi <- list()


for (i in seq_along(PEnS_model_list_no_random)){
  
  res <- summary(PEnS_model_list_no_random[[i]])
  
  PEnS_summary.models[,1:8] <- c(
    PEnS_model_list_no_random_notation[[i]], 
    res$AICtab[1],
    AICcmodavg::AICc(PEnS_model_list_no_random[[i]]),
    res$AICtab[2],
    NA, # as.data.frame(MuMIn::r.squaredGLMM(PEnS_model_list_no_random[[i]]))$R2m[1],
    NA, # as.data.frame(MuMIn::r.squaredGLMM(PEnS_model_list_no_random[[i]]))$R2m[1]
    "no",
    "no"
  )
  
  PEnS_summary.models.no.random[[i]] <- PEnS_summary.models
}
```

Second, the results from the models for *without random
variable* and *with zero inflation*

```
for (i in seq_along(PEnS_model_list_no_random_zi)){
  
  res <- summary(PEnS_model_list_no_random_zi[[i]])
  
  PEnS_summary.models[,1:8] <- c(
    PEnS_model_list_no_random_notation_zi[[i]], 
    res$AICtab[1],
    AICcmodavg::AICc(PEnS_model_list_no_random_zi[[i]]),
    res$AICtab[2],
    NA, #as.data.frame(MuMIn::r.squaredGLMM(PEnS_model_list_no_random_zi[[i]]))$R2m[1],
    NA, #as.data.frame(MuMIn::r.squaredGLMM(PEnS_model_list_no_random_zi[[i]]))$R2m[1]
    "no",
    "yes"
  )
  
  PEnS_summary.models.no.random_zi[[i]] <- PEnS_summary.models
}
```

Third, the results from the models for *with random variable*
and *without zero inflation*

```
for (i in seq_along(PEnS_model_list_with_random)){
  
  res <- summary(PEnS_model_list_with_random[[i]])
  
  PEnS_summary.models[,1:8] <- c(PEnS_model_list_with_random_notation[[i]], 
                                 res$AICtab[1],
                                 AICcmodavg::AICc(PEnS_model_list_with_random[[i]]),
                                 res$AICtab[2],
                                 as.data.frame(MuMIn::r.squaredGLMM(PEnS_model_list_with_random[[i]]))$R2m[1],
                                 as.data.frame(MuMIn::r.squaredGLMM(PEnS_model_list_with_random[[i]]))$R2c[1],
                                 "yes",
                                 "no"
  )
  
  PEnS_summary.models.with.random[[i]] <- PEnS_summary.models
}
```

```
## Warning: 'r.squaredGLMM' now calculates a revised statistic. See the help page.
```

```
## Warning in r.squaredGLMM.glmmTMB(PEnS_model_list_with_random[[i]]): the effects
## of zero-inflation and dispersion model are ignored
## Warning in r.squaredGLMM.glmmTMB(PEnS_model_list_with_random[[i]]): the effects
## of zero-inflation and dispersion model are ignored
## Warning in r.squaredGLMM.glmmTMB(PEnS_model_list_with_random[[i]]): the effects
## of zero-inflation and dispersion model are ignored
## Warning in r.squaredGLMM.glmmTMB(PEnS_model_list_with_random[[i]]): the effects
## of zero-inflation and dispersion model are ignored
## Warning in r.squaredGLMM.glmmTMB(PEnS_model_list_with_random[[i]]): the effects
## of zero-inflation and dispersion model are ignored
## Warning in r.squaredGLMM.glmmTMB(PEnS_model_list_with_random[[i]]): the effects
## of zero-inflation and dispersion model are ignored
## Warning in r.squaredGLMM.glmmTMB(PEnS_model_list_with_random[[i]]): the effects
## of zero-inflation and dispersion model are ignored
## Warning in r.squaredGLMM.glmmTMB(PEnS_model_list_with_random[[i]]): the effects
## of zero-inflation and dispersion model are ignored
## Warning in r.squaredGLMM.glmmTMB(PEnS_model_list_with_random[[i]]): the effects
## of zero-inflation and dispersion model are ignored
## Warning in r.squaredGLMM.glmmTMB(PEnS_model_list_with_random[[i]]): the effects
## of zero-inflation and dispersion model are ignored
## Warning in r.squaredGLMM.glmmTMB(PEnS_model_list_with_random[[i]]): the effects
## of zero-inflation and dispersion model are ignored
## Warning in r.squaredGLMM.glmmTMB(PEnS_model_list_with_random[[i]]): the effects
## of zero-inflation and dispersion model are ignored
## Warning in r.squaredGLMM.glmmTMB(PEnS_model_list_with_random[[i]]): the effects
## of zero-inflation and dispersion model are ignored
## Warning in r.squaredGLMM.glmmTMB(PEnS_model_list_with_random[[i]]): the effects
## of zero-inflation and dispersion model are ignored
## Warning in r.squaredGLMM.glmmTMB(PEnS_model_list_with_random[[i]]): the effects
## of zero-inflation and dispersion model are ignored
## Warning in r.squaredGLMM.glmmTMB(PEnS_model_list_with_random[[i]]): the effects
## of zero-inflation and dispersion model are ignored
## Warning in r.squaredGLMM.glmmTMB(PEnS_model_list_with_random[[i]]): the effects
## of zero-inflation and dispersion model are ignored
## Warning in r.squaredGLMM.glmmTMB(PEnS_model_list_with_random[[i]]): the effects
## of zero-inflation and dispersion model are ignored
## Warning in r.squaredGLMM.glmmTMB(PEnS_model_list_with_random[[i]]): the effects
## of zero-inflation and dispersion model are ignored
## Warning in r.squaredGLMM.glmmTMB(PEnS_model_list_with_random[[i]]): the effects
## of zero-inflation and dispersion model are ignored
## Warning in r.squaredGLMM.glmmTMB(PEnS_model_list_with_random[[i]]): the effects
## of zero-inflation and dispersion model are ignored
## Warning in r.squaredGLMM.glmmTMB(PEnS_model_list_with_random[[i]]): the effects
## of zero-inflation and dispersion model are ignored
## Warning in r.squaredGLMM.glmmTMB(PEnS_model_list_with_random[[i]]): the effects
## of zero-inflation and dispersion model are ignored
## Warning in r.squaredGLMM.glmmTMB(PEnS_model_list_with_random[[i]]): the effects
## of zero-inflation and dispersion model are ignored
## Warning in r.squaredGLMM.glmmTMB(PEnS_model_list_with_random[[i]]): the effects
## of zero-inflation and dispersion model are ignored
## Warning in r.squaredGLMM.glmmTMB(PEnS_model_list_with_random[[i]]): the effects
## of zero-inflation and dispersion model are ignored
## Warning in r.squaredGLMM.glmmTMB(PEnS_model_list_with_random[[i]]): the effects
## of zero-inflation and dispersion model are ignored
## Warning in r.squaredGLMM.glmmTMB(PEnS_model_list_with_random[[i]]): the effects
## of zero-inflation and dispersion model are ignored
## Warning in r.squaredGLMM.glmmTMB(PEnS_model_list_with_random[[i]]): the effects
## of zero-inflation and dispersion model are ignored
## Warning in r.squaredGLMM.glmmTMB(PEnS_model_list_with_random[[i]]): the effects
## of zero-inflation and dispersion model are ignored
## Warning in r.squaredGLMM.glmmTMB(PEnS_model_list_with_random[[i]]): the effects
## of zero-inflation and dispersion model are ignored
## Warning in r.squaredGLMM.glmmTMB(PEnS_model_list_with_random[[i]]): the effects
## of zero-inflation and dispersion model are ignored
## Warning in r.squaredGLMM.glmmTMB(PEnS_model_list_with_random[[i]]): the effects
## of zero-inflation and dispersion model are ignored
## Warning in r.squaredGLMM.glmmTMB(PEnS_model_list_with_random[[i]]): the effects
## of zero-inflation and dispersion model are ignored
## Warning in r.squaredGLMM.glmmTMB(PEnS_model_list_with_random[[i]]): the effects
## of zero-inflation and dispersion model are ignored
## Warning in r.squaredGLMM.glmmTMB(PEnS_model_list_with_random[[i]]): the effects
## of zero-inflation and dispersion model are ignored
## Warning in r.squaredGLMM.glmmTMB(PEnS_model_list_with_random[[i]]): the effects
## of zero-inflation and dispersion model are ignored
```

```
## Warning: Can't compute random effect variances. Some variance components equal
##   zero. Your model may suffer from singularity (see `?lme4::isSingular`
##   and `?performance::check_singularity`).
##   Solution: Respecify random structure! You may also decrease the
##   `tolerance` level to enforce the calculation of random effect variances.
```

```
## Warning in r.squaredGLMM.glmmTMB(PEnS_model_list_with_random[[i]]): the effects
## of zero-inflation and dispersion model are ignored
```

```
## Warning: Can't compute random effect variances. Some variance components equal
##   zero. Your model may suffer from singularity (see `?lme4::isSingular`
##   and `?performance::check_singularity`).
##   Solution: Respecify random structure! You may also decrease the
##   `tolerance` level to enforce the calculation of random effect variances.
```

```
## Warning in r.squaredGLMM.glmmTMB(PEnS_model_list_with_random[[i]]): the effects
## of zero-inflation and dispersion model are ignored
## Warning in r.squaredGLMM.glmmTMB(PEnS_model_list_with_random[[i]]): the effects
## of zero-inflation and dispersion model are ignored
## Warning in r.squaredGLMM.glmmTMB(PEnS_model_list_with_random[[i]]): the effects
## of zero-inflation and dispersion model are ignored
## Warning in r.squaredGLMM.glmmTMB(PEnS_model_list_with_random[[i]]): the effects
## of zero-inflation and dispersion model are ignored
## Warning in r.squaredGLMM.glmmTMB(PEnS_model_list_with_random[[i]]): the effects
## of zero-inflation and dispersion model are ignored
## Warning in r.squaredGLMM.glmmTMB(PEnS_model_list_with_random[[i]]): the effects
## of zero-inflation and dispersion model are ignored
## Warning in r.squaredGLMM.glmmTMB(PEnS_model_list_with_random[[i]]): the effects
## of zero-inflation and dispersion model are ignored
```

```
## Warning: Can't compute random effect variances. Some variance components equal
##   zero. Your model may suffer from singularity (see `?lme4::isSingular`
##   and `?performance::check_singularity`).
##   Solution: Respecify random structure! You may also decrease the
##   `tolerance` level to enforce the calculation of random effect variances.
```

```
## Warning in r.squaredGLMM.glmmTMB(PEnS_model_list_with_random[[i]]): the effects
## of zero-inflation and dispersion model are ignored
```

```
## Warning: Can't compute random effect variances. Some variance components equal
##   zero. Your model may suffer from singularity (see `?lme4::isSingular`
##   and `?performance::check_singularity`).
##   Solution: Respecify random structure! You may also decrease the
##   `tolerance` level to enforce the calculation of random effect variances.
```

```
## Warning in r.squaredGLMM.glmmTMB(PEnS_model_list_with_random[[i]]): the effects
## of zero-inflation and dispersion model are ignored
## Warning in r.squaredGLMM.glmmTMB(PEnS_model_list_with_random[[i]]): the effects
## of zero-inflation and dispersion model are ignored
## Warning in r.squaredGLMM.glmmTMB(PEnS_model_list_with_random[[i]]): the effects
## of zero-inflation and dispersion model are ignored
## Warning in r.squaredGLMM.glmmTMB(PEnS_model_list_with_random[[i]]): the effects
## of zero-inflation and dispersion model are ignored
## Warning in r.squaredGLMM.glmmTMB(PEnS_model_list_with_random[[i]]): the effects
## of zero-inflation and dispersion model are ignored
## Warning in r.squaredGLMM.glmmTMB(PEnS_model_list_with_random[[i]]): the effects
## of zero-inflation and dispersion model are ignored
## Warning in r.squaredGLMM.glmmTMB(PEnS_model_list_with_random[[i]]): the effects
## of zero-inflation and dispersion model are ignored
## Warning in r.squaredGLMM.glmmTMB(PEnS_model_list_with_random[[i]]): the effects
## of zero-inflation and dispersion model are ignored
## Warning in r.squaredGLMM.glmmTMB(PEnS_model_list_with_random[[i]]): the effects
## of zero-inflation and dispersion model are ignored
## Warning in r.squaredGLMM.glmmTMB(PEnS_model_list_with_random[[i]]): the effects
## of zero-inflation and dispersion model are ignored
## Warning in r.squaredGLMM.glmmTMB(PEnS_model_list_with_random[[i]]): the effects
## of zero-inflation and dispersion model are ignored
## Warning in r.squaredGLMM.glmmTMB(PEnS_model_list_with_random[[i]]): the effects
## of zero-inflation and dispersion model are ignored
## Warning in r.squaredGLMM.glmmTMB(PEnS_model_list_with_random[[i]]): the effects
## of zero-inflation and dispersion model are ignored
## Warning in r.squaredGLMM.glmmTMB(PEnS_model_list_with_random[[i]]): the effects
## of zero-inflation and dispersion model are ignored
## Warning in r.squaredGLMM.glmmTMB(PEnS_model_list_with_random[[i]]): the effects
## of zero-inflation and dispersion model are ignored
## Warning in r.squaredGLMM.glmmTMB(PEnS_model_list_with_random[[i]]): the effects
## of zero-inflation and dispersion model are ignored
## Warning in r.squaredGLMM.glmmTMB(PEnS_model_list_with_random[[i]]): the effects
## of zero-inflation and dispersion model are ignored
## Warning in r.squaredGLMM.glmmTMB(PEnS_model_list_with_random[[i]]): the effects
## of zero-inflation and dispersion model are ignored
## Warning in r.squaredGLMM.glmmTMB(PEnS_model_list_with_random[[i]]): the effects
## of zero-inflation and dispersion model are ignored
## Warning in r.squaredGLMM.glmmTMB(PEnS_model_list_with_random[[i]]): the effects
## of zero-inflation and dispersion model are ignored
## Warning in r.squaredGLMM.glmmTMB(PEnS_model_list_with_random[[i]]): the effects
## of zero-inflation and dispersion model are ignored
```

```
## Warning: Can't compute random effect variances. Some variance components equal
##   zero. Your model may suffer from singularity (see `?lme4::isSingular`
##   and `?performance::check_singularity`).
##   Solution: Respecify random structure! You may also decrease the
##   `tolerance` level to enforce the calculation of random effect variances.
```

```
## Warning in r.squaredGLMM.glmmTMB(PEnS_model_list_with_random[[i]]): the effects
## of zero-inflation and dispersion model are ignored
```

```
## Warning: Can't compute random effect variances. Some variance components equal
##   zero. Your model may suffer from singularity (see `?lme4::isSingular`
##   and `?performance::check_singularity`).
##   Solution: Respecify random structure! You may also decrease the
##   `tolerance` level to enforce the calculation of random effect variances.
```

```
## Warning in r.squaredGLMM.glmmTMB(PEnS_model_list_with_random[[i]]): the effects
## of zero-inflation and dispersion model are ignored
## Warning in r.squaredGLMM.glmmTMB(PEnS_model_list_with_random[[i]]): the effects
## of zero-inflation and dispersion model are ignored
## Warning in r.squaredGLMM.glmmTMB(PEnS_model_list_with_random[[i]]): the effects
## of zero-inflation and dispersion model are ignored
## Warning in r.squaredGLMM.glmmTMB(PEnS_model_list_with_random[[i]]): the effects
## of zero-inflation and dispersion model are ignored
## Warning in r.squaredGLMM.glmmTMB(PEnS_model_list_with_random[[i]]): the effects
## of zero-inflation and dispersion model are ignored
## Warning in r.squaredGLMM.glmmTMB(PEnS_model_list_with_random[[i]]): the effects
## of zero-inflation and dispersion model are ignored
## Warning in r.squaredGLMM.glmmTMB(PEnS_model_list_with_random[[i]]): the effects
## of zero-inflation and dispersion model are ignored
## Warning in r.squaredGLMM.glmmTMB(PEnS_model_list_with_random[[i]]): the effects
## of zero-inflation and dispersion model are ignored
## Warning in r.squaredGLMM.glmmTMB(PEnS_model_list_with_random[[i]]): the effects
## of zero-inflation and dispersion model are ignored
## Warning in r.squaredGLMM.glmmTMB(PEnS_model_list_with_random[[i]]): the effects
## of zero-inflation and dispersion model are ignored
## Warning in r.squaredGLMM.glmmTMB(PEnS_model_list_with_random[[i]]): the effects
## of zero-inflation and dispersion model are ignored
## Warning in r.squaredGLMM.glmmTMB(PEnS_model_list_with_random[[i]]): the effects
## of zero-inflation and dispersion model are ignored
## Warning in r.squaredGLMM.glmmTMB(PEnS_model_list_with_random[[i]]): the effects
## of zero-inflation and dispersion model are ignored
## Warning in r.squaredGLMM.glmmTMB(PEnS_model_list_with_random[[i]]): the effects
## of zero-inflation and dispersion model are ignored
## Warning in r.squaredGLMM.glmmTMB(PEnS_model_list_with_random[[i]]): the effects
## of zero-inflation and dispersion model are ignored
## Warning in r.squaredGLMM.glmmTMB(PEnS_model_list_with_random[[i]]): the effects
## of zero-inflation and dispersion model are ignored
## Warning in r.squaredGLMM.glmmTMB(PEnS_model_list_with_random[[i]]): the effects
## of zero-inflation and dispersion model are ignored
## Warning in r.squaredGLMM.glmmTMB(PEnS_model_list_with_random[[i]]): the effects
## of zero-inflation and dispersion model are ignored
## Warning in r.squaredGLMM.glmmTMB(PEnS_model_list_with_random[[i]]): the effects
## of zero-inflation and dispersion model are ignored
## Warning in r.squaredGLMM.glmmTMB(PEnS_model_list_with_random[[i]]): the effects
## of zero-inflation and dispersion model are ignored
## Warning in r.squaredGLMM.glmmTMB(PEnS_model_list_with_random[[i]]): the effects
## of zero-inflation and dispersion model are ignored
## Warning in r.squaredGLMM.glmmTMB(PEnS_model_list_with_random[[i]]): the effects
## of zero-inflation and dispersion model are ignored
## Warning in r.squaredGLMM.glmmTMB(PEnS_model_list_with_random[[i]]): the effects
## of zero-inflation and dispersion model are ignored
## Warning in r.squaredGLMM.glmmTMB(PEnS_model_list_with_random[[i]]): the effects
## of zero-inflation and dispersion model are ignored
## Warning in r.squaredGLMM.glmmTMB(PEnS_model_list_with_random[[i]]): the effects
## of zero-inflation and dispersion model are ignored
## Warning in r.squaredGLMM.glmmTMB(PEnS_model_list_with_random[[i]]): the effects
## of zero-inflation and dispersion model are ignored
## Warning in r.squaredGLMM.glmmTMB(PEnS_model_list_with_random[[i]]): the effects
## of zero-inflation and dispersion model are ignored
## Warning in r.squaredGLMM.glmmTMB(PEnS_model_list_with_random[[i]]): the effects
## of zero-inflation and dispersion model are ignored
## Warning in r.squaredGLMM.glmmTMB(PEnS_model_list_with_random[[i]]): the effects
## of zero-inflation and dispersion model are ignored
## Warning in r.squaredGLMM.glmmTMB(PEnS_model_list_with_random[[i]]): the effects
## of zero-inflation and dispersion model are ignored
## Warning in r.squaredGLMM.glmmTMB(PEnS_model_list_with_random[[i]]): the effects
## of zero-inflation and dispersion model are ignored
## Warning in r.squaredGLMM.glmmTMB(PEnS_model_list_with_random[[i]]): the effects
## of zero-inflation and dispersion model are ignored
## Warning in r.squaredGLMM.glmmTMB(PEnS_model_list_with_random[[i]]): the effects
## of zero-inflation and dispersion model are ignored
## Warning in r.squaredGLMM.glmmTMB(PEnS_model_list_with_random[[i]]): the effects
## of zero-inflation and dispersion model are ignored
## Warning in r.squaredGLMM.glmmTMB(PEnS_model_list_with_random[[i]]): the effects
## of zero-inflation and dispersion model are ignored
## Warning in r.squaredGLMM.glmmTMB(PEnS_model_list_with_random[[i]]): the effects
## of zero-inflation and dispersion model are ignored
## Warning in r.squaredGLMM.glmmTMB(PEnS_model_list_with_random[[i]]): the effects
## of zero-inflation and dispersion model are ignored
## Warning in r.squaredGLMM.glmmTMB(PEnS_model_list_with_random[[i]]): the effects
## of zero-inflation and dispersion model are ignored
## Warning in r.squaredGLMM.glmmTMB(PEnS_model_list_with_random[[i]]): the effects
## of zero-inflation and dispersion model are ignored
```

```
## Warning: Can't compute random effect variances. Some variance components equal
##   zero. Your model may suffer from singularity (see `?lme4::isSingular`
##   and `?performance::check_singularity`).
##   Solution: Respecify random structure! You may also decrease the
##   `tolerance` level to enforce the calculation of random effect variances.
```

```
## Warning in r.squaredGLMM.glmmTMB(PEnS_model_list_with_random[[i]]): the effects
## of zero-inflation and dispersion model are ignored
```

```
## Warning: Can't compute random effect variances. Some variance components equal
##   zero. Your model may suffer from singularity (see `?lme4::isSingular`
##   and `?performance::check_singularity`).
##   Solution: Respecify random structure! You may also decrease the
##   `tolerance` level to enforce the calculation of random effect variances.
```

```
## Warning in r.squaredGLMM.glmmTMB(PEnS_model_list_with_random[[i]]): the effects
## of zero-inflation and dispersion model are ignored
```

```
## Warning: Can't compute random effect variances. Some variance components equal
##   zero. Your model may suffer from singularity (see `?lme4::isSingular`
##   and `?performance::check_singularity`).
##   Solution: Respecify random structure! You may also decrease the
##   `tolerance` level to enforce the calculation of random effect variances.
```

```
## Warning in r.squaredGLMM.glmmTMB(PEnS_model_list_with_random[[i]]): the effects
## of zero-inflation and dispersion model are ignored
```

```
## Warning: Can't compute random effect variances. Some variance components equal
##   zero. Your model may suffer from singularity (see `?lme4::isSingular`
##   and `?performance::check_singularity`).
##   Solution: Respecify random structure! You may also decrease the
##   `tolerance` level to enforce the calculation of random effect variances.
```

```
## Warning in r.squaredGLMM.glmmTMB(PEnS_model_list_with_random[[i]]): the effects
## of zero-inflation and dispersion model are ignored
```

```
## Warning: Can't compute random effect variances. Some variance components equal
##   zero. Your model may suffer from singularity (see `?lme4::isSingular`
##   and `?performance::check_singularity`).
##   Solution: Respecify random structure! You may also decrease the
##   `tolerance` level to enforce the calculation of random effect variances.
```

```
## Warning in r.squaredGLMM.glmmTMB(PEnS_model_list_with_random[[i]]): the effects
## of zero-inflation and dispersion model are ignored
```

```
## Warning: Can't compute random effect variances. Some variance components equal
##   zero. Your model may suffer from singularity (see `?lme4::isSingular`
##   and `?performance::check_singularity`).
##   Solution: Respecify random structure! You may also decrease the
##   `tolerance` level to enforce the calculation of random effect variances.
```

```
## Warning in r.squaredGLMM.glmmTMB(PEnS_model_list_with_random[[i]]): the effects
## of zero-inflation and dispersion model are ignored
## Warning in r.squaredGLMM.glmmTMB(PEnS_model_list_with_random[[i]]): the effects
## of zero-inflation and dispersion model are ignored
## Warning in r.squaredGLMM.glmmTMB(PEnS_model_list_with_random[[i]]): the effects
## of zero-inflation and dispersion model are ignored
## Warning in r.squaredGLMM.glmmTMB(PEnS_model_list_with_random[[i]]): the effects
## of zero-inflation and dispersion model are ignored
## Warning in r.squaredGLMM.glmmTMB(PEnS_model_list_with_random[[i]]): the effects
## of zero-inflation and dispersion model are ignored
## Warning in r.squaredGLMM.glmmTMB(PEnS_model_list_with_random[[i]]): the effects
## of zero-inflation and dispersion model are ignored
## Warning in r.squaredGLMM.glmmTMB(PEnS_model_list_with_random[[i]]): the effects
## of zero-inflation and dispersion model are ignored
```

```
## Warning: Can't compute random effect variances. Some variance components equal
##   zero. Your model may suffer from singularity (see `?lme4::isSingular`
##   and `?performance::check_singularity`).
##   Solution: Respecify random structure! You may also decrease the
##   `tolerance` level to enforce the calculation of random effect variances.
```

```
## Warning in r.squaredGLMM.glmmTMB(PEnS_model_list_with_random[[i]]): the effects
## of zero-inflation and dispersion model are ignored
```

```
## Warning: Can't compute random effect variances. Some variance components equal
##   zero. Your model may suffer from singularity (see `?lme4::isSingular`
##   and `?performance::check_singularity`).
##   Solution: Respecify random structure! You may also decrease the
##   `tolerance` level to enforce the calculation of random effect variances.
```

```
## Warning in r.squaredGLMM.glmmTMB(PEnS_model_list_with_random[[i]]): the effects
## of zero-inflation and dispersion model are ignored
```

```
## Warning: Can't compute random effect variances. Some variance components equal
##   zero. Your model may suffer from singularity (see `?lme4::isSingular`
##   and `?performance::check_singularity`).
##   Solution: Respecify random structure! You may also decrease the
##   `tolerance` level to enforce the calculation of random effect variances.
```

```
## Warning in r.squaredGLMM.glmmTMB(PEnS_model_list_with_random[[i]]): the effects
## of zero-inflation and dispersion model are ignored
```

```
## Warning: Can't compute random effect variances. Some variance components equal
##   zero. Your model may suffer from singularity (see `?lme4::isSingular`
##   and `?performance::check_singularity`).
##   Solution: Respecify random structure! You may also decrease the
##   `tolerance` level to enforce the calculation of random effect variances.
```

```
## Warning in r.squaredGLMM.glmmTMB(PEnS_model_list_with_random[[i]]): the effects
## of zero-inflation and dispersion model are ignored
## Warning in r.squaredGLMM.glmmTMB(PEnS_model_list_with_random[[i]]): the effects
## of zero-inflation and dispersion model are ignored
## Warning in r.squaredGLMM.glmmTMB(PEnS_model_list_with_random[[i]]): the effects
## of zero-inflation and dispersion model are ignored
## Warning in r.squaredGLMM.glmmTMB(PEnS_model_list_with_random[[i]]): the effects
## of zero-inflation and dispersion model are ignored
## Warning in r.squaredGLMM.glmmTMB(PEnS_model_list_with_random[[i]]): the effects
## of zero-inflation and dispersion model are ignored
## Warning in r.squaredGLMM.glmmTMB(PEnS_model_list_with_random[[i]]): the effects
## of zero-inflation and dispersion model are ignored
## Warning in r.squaredGLMM.glmmTMB(PEnS_model_list_with_random[[i]]): the effects
## of zero-inflation and dispersion model are ignored
## Warning in r.squaredGLMM.glmmTMB(PEnS_model_list_with_random[[i]]): the effects
## of zero-inflation and dispersion model are ignored
## Warning in r.squaredGLMM.glmmTMB(PEnS_model_list_with_random[[i]]): the effects
## of zero-inflation and dispersion model are ignored
## Warning in r.squaredGLMM.glmmTMB(PEnS_model_list_with_random[[i]]): the effects
## of zero-inflation and dispersion model are ignored
## Warning in r.squaredGLMM.glmmTMB(PEnS_model_list_with_random[[i]]): the effects
## of zero-inflation and dispersion model are ignored
## Warning in r.squaredGLMM.glmmTMB(PEnS_model_list_with_random[[i]]): the effects
## of zero-inflation and dispersion model are ignored
## Warning in r.squaredGLMM.glmmTMB(PEnS_model_list_with_random[[i]]): the effects
## of zero-inflation and dispersion model are ignored
## Warning in r.squaredGLMM.glmmTMB(PEnS_model_list_with_random[[i]]): the effects
## of zero-inflation and dispersion model are ignored
## Warning in r.squaredGLMM.glmmTMB(PEnS_model_list_with_random[[i]]): the effects
## of zero-inflation and dispersion model are ignored
## Warning in r.squaredGLMM.glmmTMB(PEnS_model_list_with_random[[i]]): the effects
## of zero-inflation and dispersion model are ignored
## Warning in r.squaredGLMM.glmmTMB(PEnS_model_list_with_random[[i]]): the effects
## of zero-inflation and dispersion model are ignored
## Warning in r.squaredGLMM.glmmTMB(PEnS_model_list_with_random[[i]]): the effects
## of zero-inflation and dispersion model are ignored
## Warning in r.squaredGLMM.glmmTMB(PEnS_model_list_with_random[[i]]): the effects
## of zero-inflation and dispersion model are ignored
## Warning in r.squaredGLMM.glmmTMB(PEnS_model_list_with_random[[i]]): the effects
## of zero-inflation and dispersion model are ignored
## Warning in r.squaredGLMM.glmmTMB(PEnS_model_list_with_random[[i]]): the effects
## of zero-inflation and dispersion model are ignored
## Warning in r.squaredGLMM.glmmTMB(PEnS_model_list_with_random[[i]]): the effects
## of zero-inflation and dispersion model are ignored
## Warning in r.squaredGLMM.glmmTMB(PEnS_model_list_with_random[[i]]): the effects
## of zero-inflation and dispersion model are ignored
## Warning in r.squaredGLMM.glmmTMB(PEnS_model_list_with_random[[i]]): the effects
## of zero-inflation and dispersion model are ignored
## Warning in r.squaredGLMM.glmmTMB(PEnS_model_list_with_random[[i]]): the effects
## of zero-inflation and dispersion model are ignored
```

```
## Warning: Can't compute random effect variances. Some variance components equal
##   zero. Your model may suffer from singularity (see `?lme4::isSingular`
##   and `?performance::check_singularity`).
##   Solution: Respecify random structure! You may also decrease the
##   `tolerance` level to enforce the calculation of random effect variances.
```

```
## Warning in r.squaredGLMM.glmmTMB(PEnS_model_list_with_random[[i]]): the effects
## of zero-inflation and dispersion model are ignored
```

```
## Warning: Can't compute random effect variances. Some variance components equal
##   zero. Your model may suffer from singularity (see `?lme4::isSingular`
##   and `?performance::check_singularity`).
##   Solution: Respecify random structure! You may also decrease the
##   `tolerance` level to enforce the calculation of random effect variances.
```

```
## Warning in r.squaredGLMM.glmmTMB(PEnS_model_list_with_random[[i]]): the effects
## of zero-inflation and dispersion model are ignored
```

```
## Warning: Can't compute random effect variances. Some variance components equal
##   zero. Your model may suffer from singularity (see `?lme4::isSingular`
##   and `?performance::check_singularity`).
##   Solution: Respecify random structure! You may also decrease the
##   `tolerance` level to enforce the calculation of random effect variances.
```

```
## Warning in r.squaredGLMM.glmmTMB(PEnS_model_list_with_random[[i]]): the effects
## of zero-inflation and dispersion model are ignored
```

```
## Warning: Can't compute random effect variances. Some variance components equal
##   zero. Your model may suffer from singularity (see `?lme4::isSingular`
##   and `?performance::check_singularity`).
##   Solution: Respecify random structure! You may also decrease the
##   `tolerance` level to enforce the calculation of random effect variances.
```

```
## Warning in r.squaredGLMM.glmmTMB(PEnS_model_list_with_random[[i]]): the effects
## of zero-inflation and dispersion model are ignored
## Warning in r.squaredGLMM.glmmTMB(PEnS_model_list_with_random[[i]]): the effects
## of zero-inflation and dispersion model are ignored
## Warning in r.squaredGLMM.glmmTMB(PEnS_model_list_with_random[[i]]): the effects
## of zero-inflation and dispersion model are ignored
## Warning in r.squaredGLMM.glmmTMB(PEnS_model_list_with_random[[i]]): the effects
## of zero-inflation and dispersion model are ignored
## Warning in r.squaredGLMM.glmmTMB(PEnS_model_list_with_random[[i]]): the effects
## of zero-inflation and dispersion model are ignored
## Warning in r.squaredGLMM.glmmTMB(PEnS_model_list_with_random[[i]]): the effects
## of zero-inflation and dispersion model are ignored
## Warning in r.squaredGLMM.glmmTMB(PEnS_model_list_with_random[[i]]): the effects
## of zero-inflation and dispersion model are ignored
## Warning in r.squaredGLMM.glmmTMB(PEnS_model_list_with_random[[i]]): the effects
## of zero-inflation and dispersion model are ignored
## Warning in r.squaredGLMM.glmmTMB(PEnS_model_list_with_random[[i]]): the effects
## of zero-inflation and dispersion model are ignored
## Warning in r.squaredGLMM.glmmTMB(PEnS_model_list_with_random[[i]]): the effects
## of zero-inflation and dispersion model are ignored
## Warning in r.squaredGLMM.glmmTMB(PEnS_model_list_with_random[[i]]): the effects
## of zero-inflation and dispersion model are ignored
## Warning in r.squaredGLMM.glmmTMB(PEnS_model_list_with_random[[i]]): the effects
## of zero-inflation and dispersion model are ignored
## Warning in r.squaredGLMM.glmmTMB(PEnS_model_list_with_random[[i]]): the effects
## of zero-inflation and dispersion model are ignored
## Warning in r.squaredGLMM.glmmTMB(PEnS_model_list_with_random[[i]]): the effects
## of zero-inflation and dispersion model are ignored
## Warning in r.squaredGLMM.glmmTMB(PEnS_model_list_with_random[[i]]): the effects
## of zero-inflation and dispersion model are ignored
## Warning in r.squaredGLMM.glmmTMB(PEnS_model_list_with_random[[i]]): the effects
## of zero-inflation and dispersion model are ignored
## Warning in r.squaredGLMM.glmmTMB(PEnS_model_list_with_random[[i]]): the effects
## of zero-inflation and dispersion model are ignored
## Warning in r.squaredGLMM.glmmTMB(PEnS_model_list_with_random[[i]]): the effects
## of zero-inflation and dispersion model are ignored
## Warning in r.squaredGLMM.glmmTMB(PEnS_model_list_with_random[[i]]): the effects
## of zero-inflation and dispersion model are ignored
## Warning in r.squaredGLMM.glmmTMB(PEnS_model_list_with_random[[i]]): the effects
## of zero-inflation and dispersion model are ignored
## Warning in r.squaredGLMM.glmmTMB(PEnS_model_list_with_random[[i]]): the effects
## of zero-inflation and dispersion model are ignored
## Warning in r.squaredGLMM.glmmTMB(PEnS_model_list_with_random[[i]]): the effects
## of zero-inflation and dispersion model are ignored
## Warning in r.squaredGLMM.glmmTMB(PEnS_model_list_with_random[[i]]): the effects
## of zero-inflation and dispersion model are ignored
## Warning in r.squaredGLMM.glmmTMB(PEnS_model_list_with_random[[i]]): the effects
## of zero-inflation and dispersion model are ignored
## Warning in r.squaredGLMM.glmmTMB(PEnS_model_list_with_random[[i]]): the effects
## of zero-inflation and dispersion model are ignored
## Warning in r.squaredGLMM.glmmTMB(PEnS_model_list_with_random[[i]]): the effects
## of zero-inflation and dispersion model are ignored
## Warning in r.squaredGLMM.glmmTMB(PEnS_model_list_with_random[[i]]): the effects
## of zero-inflation and dispersion model are ignored
## Warning in r.squaredGLMM.glmmTMB(PEnS_model_list_with_random[[i]]): the effects
## of zero-inflation and dispersion model are ignored
## Warning in r.squaredGLMM.glmmTMB(PEnS_model_list_with_random[[i]]): the effects
## of zero-inflation and dispersion model are ignored
## Warning in r.squaredGLMM.glmmTMB(PEnS_model_list_with_random[[i]]): the effects
## of zero-inflation and dispersion model are ignored
## Warning in r.squaredGLMM.glmmTMB(PEnS_model_list_with_random[[i]]): the effects
## of zero-inflation and dispersion model are ignored
## Warning in r.squaredGLMM.glmmTMB(PEnS_model_list_with_random[[i]]): the effects
## of zero-inflation and dispersion model are ignored
## Warning in r.squaredGLMM.glmmTMB(PEnS_model_list_with_random[[i]]): the effects
## of zero-inflation and dispersion model are ignored
## Warning in r.squaredGLMM.glmmTMB(PEnS_model_list_with_random[[i]]): the effects
## of zero-inflation and dispersion model are ignored
## Warning in r.squaredGLMM.glmmTMB(PEnS_model_list_with_random[[i]]): the effects
## of zero-inflation and dispersion model are ignored
## Warning in r.squaredGLMM.glmmTMB(PEnS_model_list_with_random[[i]]): the effects
## of zero-inflation and dispersion model are ignored
## Warning in r.squaredGLMM.glmmTMB(PEnS_model_list_with_random[[i]]): the effects
## of zero-inflation and dispersion model are ignored
```

```
## Warning: Can't compute random effect variances. Some variance components equal
##   zero. Your model may suffer from singularity (see `?lme4::isSingular`
##   and `?performance::check_singularity`).
##   Solution: Respecify random structure! You may also decrease the
##   `tolerance` level to enforce the calculation of random effect variances.
```

```
## Warning in r.squaredGLMM.glmmTMB(PEnS_model_list_with_random[[i]]): the effects
## of zero-inflation and dispersion model are ignored
```

```
## Warning: Can't compute random effect variances. Some variance components equal
##   zero. Your model may suffer from singularity (see `?lme4::isSingular`
##   and `?performance::check_singularity`).
##   Solution: Respecify random structure! You may also decrease the
##   `tolerance` level to enforce the calculation of random effect variances.
```

```
## Warning in r.squaredGLMM.glmmTMB(PEnS_model_list_with_random[[i]]): the effects
## of zero-inflation and dispersion model are ignored
```

```
## Warning: Can't compute random effect variances. Some variance components equal
##   zero. Your model may suffer from singularity (see `?lme4::isSingular`
##   and `?performance::check_singularity`).
##   Solution: Respecify random structure! You may also decrease the
##   `tolerance` level to enforce the calculation of random effect variances.
```

```
## Warning in r.squaredGLMM.glmmTMB(PEnS_model_list_with_random[[i]]): the effects
## of zero-inflation and dispersion model are ignored
```

```
## Warning: Can't compute random effect variances. Some variance components equal
##   zero. Your model may suffer from singularity (see `?lme4::isSingular`
##   and `?performance::check_singularity`).
##   Solution: Respecify random structure! You may also decrease the
##   `tolerance` level to enforce the calculation of random effect variances.
```

```
## Warning in r.squaredGLMM.glmmTMB(PEnS_model_list_with_random[[i]]): the effects
## of zero-inflation and dispersion model are ignored
## Warning in r.squaredGLMM.glmmTMB(PEnS_model_list_with_random[[i]]): the effects
## of zero-inflation and dispersion model are ignored
## Warning in r.squaredGLMM.glmmTMB(PEnS_model_list_with_random[[i]]): the effects
## of zero-inflation and dispersion model are ignored
## Warning in r.squaredGLMM.glmmTMB(PEnS_model_list_with_random[[i]]): the effects
## of zero-inflation and dispersion model are ignored
## Warning in r.squaredGLMM.glmmTMB(PEnS_model_list_with_random[[i]]): the effects
## of zero-inflation and dispersion model are ignored
## Warning in r.squaredGLMM.glmmTMB(PEnS_model_list_with_random[[i]]): the effects
## of zero-inflation and dispersion model are ignored
## Warning in r.squaredGLMM.glmmTMB(PEnS_model_list_with_random[[i]]): the effects
## of zero-inflation and dispersion model are ignored
## Warning in r.squaredGLMM.glmmTMB(PEnS_model_list_with_random[[i]]): the effects
## of zero-inflation and dispersion model are ignored
## Warning in r.squaredGLMM.glmmTMB(PEnS_model_list_with_random[[i]]): the effects
## of zero-inflation and dispersion model are ignored
## Warning in r.squaredGLMM.glmmTMB(PEnS_model_list_with_random[[i]]): the effects
## of zero-inflation and dispersion model are ignored
## Warning in r.squaredGLMM.glmmTMB(PEnS_model_list_with_random[[i]]): the effects
## of zero-inflation and dispersion model are ignored
## Warning in r.squaredGLMM.glmmTMB(PEnS_model_list_with_random[[i]]): the effects
## of zero-inflation and dispersion model are ignored
## Warning in r.squaredGLMM.glmmTMB(PEnS_model_list_with_random[[i]]): the effects
## of zero-inflation and dispersion model are ignored
## Warning in r.squaredGLMM.glmmTMB(PEnS_model_list_with_random[[i]]): the effects
## of zero-inflation and dispersion model are ignored
## Warning in r.squaredGLMM.glmmTMB(PEnS_model_list_with_random[[i]]): the effects
## of zero-inflation and dispersion model are ignored
## Warning in r.squaredGLMM.glmmTMB(PEnS_model_list_with_random[[i]]): the effects
## of zero-inflation and dispersion model are ignored
## Warning in r.squaredGLMM.glmmTMB(PEnS_model_list_with_random[[i]]): the effects
## of zero-inflation and dispersion model are ignored
## Warning in r.squaredGLMM.glmmTMB(PEnS_model_list_with_random[[i]]): the effects
## of zero-inflation and dispersion model are ignored
## Warning in r.squaredGLMM.glmmTMB(PEnS_model_list_with_random[[i]]): the effects
## of zero-inflation and dispersion model are ignored
## Warning in r.squaredGLMM.glmmTMB(PEnS_model_list_with_random[[i]]): the effects
## of zero-inflation and dispersion model are ignored
## Warning in r.squaredGLMM.glmmTMB(PEnS_model_list_with_random[[i]]): the effects
## of zero-inflation and dispersion model are ignored
## Warning in r.squaredGLMM.glmmTMB(PEnS_model_list_with_random[[i]]): the effects
## of zero-inflation and dispersion model are ignored
## Warning in r.squaredGLMM.glmmTMB(PEnS_model_list_with_random[[i]]): the effects
## of zero-inflation and dispersion model are ignored
## Warning in r.squaredGLMM.glmmTMB(PEnS_model_list_with_random[[i]]): the effects
## of zero-inflation and dispersion model are ignored
## Warning in r.squaredGLMM.glmmTMB(PEnS_model_list_with_random[[i]]): the effects
## of zero-inflation and dispersion model are ignored
## Warning in r.squaredGLMM.glmmTMB(PEnS_model_list_with_random[[i]]): the effects
## of zero-inflation and dispersion model are ignored
## Warning in r.squaredGLMM.glmmTMB(PEnS_model_list_with_random[[i]]): the effects
## of zero-inflation and dispersion model are ignored
```

```
## Warning: Can't compute random effect variances. Some variance components equal
##   zero. Your model may suffer from singularity (see `?lme4::isSingular`
##   and `?performance::check_singularity`).
##   Solution: Respecify random structure! You may also decrease the
##   `tolerance` level to enforce the calculation of random effect variances.
```

```
## Warning in r.squaredGLMM.glmmTMB(PEnS_model_list_with_random[[i]]): the effects
## of zero-inflation and dispersion model are ignored
```

```
## Warning: Can't compute random effect variances. Some variance components equal
##   zero. Your model may suffer from singularity (see `?lme4::isSingular`
##   and `?performance::check_singularity`).
##   Solution: Respecify random structure! You may also decrease the
##   `tolerance` level to enforce the calculation of random effect variances.
```

```
## Warning in r.squaredGLMM.glmmTMB(PEnS_model_list_with_random[[i]]): the effects
## of zero-inflation and dispersion model are ignored
## Warning in r.squaredGLMM.glmmTMB(PEnS_model_list_with_random[[i]]): the effects
## of zero-inflation and dispersion model are ignored
## Warning in r.squaredGLMM.glmmTMB(PEnS_model_list_with_random[[i]]): the effects
## of zero-inflation and dispersion model are ignored
## Warning in r.squaredGLMM.glmmTMB(PEnS_model_list_with_random[[i]]): the effects
## of zero-inflation and dispersion model are ignored
## Warning in r.squaredGLMM.glmmTMB(PEnS_model_list_with_random[[i]]): the effects
## of zero-inflation and dispersion model are ignored
## Warning in r.squaredGLMM.glmmTMB(PEnS_model_list_with_random[[i]]): the effects
## of zero-inflation and dispersion model are ignored
## Warning in r.squaredGLMM.glmmTMB(PEnS_model_list_with_random[[i]]): the effects
## of zero-inflation and dispersion model are ignored
## Warning in r.squaredGLMM.glmmTMB(PEnS_model_list_with_random[[i]]): the effects
## of zero-inflation and dispersion model are ignored
## Warning in r.squaredGLMM.glmmTMB(PEnS_model_list_with_random[[i]]): the effects
## of zero-inflation and dispersion model are ignored
## Warning in r.squaredGLMM.glmmTMB(PEnS_model_list_with_random[[i]]): the effects
## of zero-inflation and dispersion model are ignored
## Warning in r.squaredGLMM.glmmTMB(PEnS_model_list_with_random[[i]]): the effects
## of zero-inflation and dispersion model are ignored
## Warning in r.squaredGLMM.glmmTMB(PEnS_model_list_with_random[[i]]): the effects
## of zero-inflation and dispersion model are ignored
## Warning in r.squaredGLMM.glmmTMB(PEnS_model_list_with_random[[i]]): the effects
## of zero-inflation and dispersion model are ignored
## Warning in r.squaredGLMM.glmmTMB(PEnS_model_list_with_random[[i]]): the effects
## of zero-inflation and dispersion model are ignored
## Warning in r.squaredGLMM.glmmTMB(PEnS_model_list_with_random[[i]]): the effects
## of zero-inflation and dispersion model are ignored
## Warning in r.squaredGLMM.glmmTMB(PEnS_model_list_with_random[[i]]): the effects
## of zero-inflation and dispersion model are ignored
## Warning in r.squaredGLMM.glmmTMB(PEnS_model_list_with_random[[i]]): the effects
## of zero-inflation and dispersion model are ignored
## Warning in r.squaredGLMM.glmmTMB(PEnS_model_list_with_random[[i]]): the effects
## of zero-inflation and dispersion model are ignored
## Warning in r.squaredGLMM.glmmTMB(PEnS_model_list_with_random[[i]]): the effects
## of zero-inflation and dispersion model are ignored
```

```
## Warning: Can't compute random effect variances. Some variance components equal
##   zero. Your model may suffer from singularity (see `?lme4::isSingular`
##   and `?performance::check_singularity`).
##   Solution: Respecify random structure! You may also decrease the
##   `tolerance` level to enforce the calculation of random effect variances.
```

```
## Warning in r.squaredGLMM.glmmTMB(PEnS_model_list_with_random[[i]]): the effects
## of zero-inflation and dispersion model are ignored
```

```
## Warning: Can't compute random effect variances. Some variance components equal
##   zero. Your model may suffer from singularity (see `?lme4::isSingular`
##   and `?performance::check_singularity`).
##   Solution: Respecify random structure! You may also decrease the
##   `tolerance` level to enforce the calculation of random effect variances.
```

```
## Warning in r.squaredGLMM.glmmTMB(PEnS_model_list_with_random[[i]]): the effects
## of zero-inflation and dispersion model are ignored
## Warning in r.squaredGLMM.glmmTMB(PEnS_model_list_with_random[[i]]): the effects
## of zero-inflation and dispersion model are ignored
## Warning in r.squaredGLMM.glmmTMB(PEnS_model_list_with_random[[i]]): the effects
## of zero-inflation and dispersion model are ignored
## Warning in r.squaredGLMM.glmmTMB(PEnS_model_list_with_random[[i]]): the effects
## of zero-inflation and dispersion model are ignored
## Warning in r.squaredGLMM.glmmTMB(PEnS_model_list_with_random[[i]]): the effects
## of zero-inflation and dispersion model are ignored
## Warning in r.squaredGLMM.glmmTMB(PEnS_model_list_with_random[[i]]): the effects
## of zero-inflation and dispersion model are ignored
## Warning in r.squaredGLMM.glmmTMB(PEnS_model_list_with_random[[i]]): the effects
## of zero-inflation and dispersion model are ignored
## Warning in r.squaredGLMM.glmmTMB(PEnS_model_list_with_random[[i]]): the effects
## of zero-inflation and dispersion model are ignored
## Warning in r.squaredGLMM.glmmTMB(PEnS_model_list_with_random[[i]]): the effects
## of zero-inflation and dispersion model are ignored
## Warning in r.squaredGLMM.glmmTMB(PEnS_model_list_with_random[[i]]): the effects
## of zero-inflation and dispersion model are ignored
## Warning in r.squaredGLMM.glmmTMB(PEnS_model_list_with_random[[i]]): the effects
## of zero-inflation and dispersion model are ignored
## Warning in r.squaredGLMM.glmmTMB(PEnS_model_list_with_random[[i]]): the effects
## of zero-inflation and dispersion model are ignored
## Warning in r.squaredGLMM.glmmTMB(PEnS_model_list_with_random[[i]]): the effects
## of zero-inflation and dispersion model are ignored
## Warning in r.squaredGLMM.glmmTMB(PEnS_model_list_with_random[[i]]): the effects
## of zero-inflation and dispersion model are ignored
## Warning in r.squaredGLMM.glmmTMB(PEnS_model_list_with_random[[i]]): the effects
## of zero-inflation and dispersion model are ignored
## Warning in r.squaredGLMM.glmmTMB(PEnS_model_list_with_random[[i]]): the effects
## of zero-inflation and dispersion model are ignored
```

Finally, the results from the models for *with random
variable* and *with zero inflation*

```
for (i in seq_along(PEnS_model_list_with_random_zi)){
  
  res <- summary(PEnS_model_list_with_random_zi[[i]])
  
  PEnS_summary.models[,1:8] <- c(PEnS_model_list_with_random_notation_zi[[i]], 
                                 res$AICtab[1],
                                 AICcmodavg::AICc(PEnS_model_list_with_random_zi[[i]]),
                                 res$AICtab[2],
                                 as.data.frame(MuMIn::r.squaredGLMM(PEnS_model_list_with_random_zi[[i]]))$R2m[1],
                                 as.data.frame(MuMIn::r.squaredGLMM(PEnS_model_list_with_random_zi[[i]]))$R2c[1],
                                 "yes",
                                 "yes"
  )
  
  PEnS_summary.models.with.random_zi[[i]] <- PEnS_summary.models
}
```

## Comparing the models

Best model is selected based on AICc.

```
library(dplyr)

PEnS_data1 <- do.call(bind_rows, PEnS_summary.models.no.random)
PEnS_data2 <- do.call(bind_rows, PEnS_summary.models.no.random_zi)
PEnS_data3 <- do.call(bind_rows, PEnS_summary.models.with.random)
PEnS_data4 <- do.call(bind_rows, PEnS_summary.models.with.random_zi)

PEnS_summary.models <- bind_rows(PEnS_data1,PEnS_data2,PEnS_data3,PEnS_data4)

PEnS_summary.models$Model <- paste0("Model.", seq_len(nrow(PEnS_summary.models)))

PEnS_summary.models[, c(2:6)] <- lapply(PEnS_summary.models[, c(2:6)], as.numeric)

PEnS_summary.models$Delta_AICc <- PEnS_summary.models$AICc-(min(PEnS_summary.models$AICc, na.rm = T))

# Which is the best model based on AICc?
(PEnS_best_model <- PEnS_summary.models[order(PEnS_summary.models$AICc), ][1,])
```

```
##                                                                         formula
## 256 Endemism_proportion~scales::rescale(log1p(Maximum_depth_m)) + (1|Catchment)
##          AIC     AICc     BIC R2.mar.mumin R2.cond.mumin random_var zi
## 256 12.14612 13.31278 26.3628    0.3339928     0.8000007        yes no
##         Model Delta_AICc
## 256 Model.256          0
```

```
# Saving the results of the model comparisons
write.csv(PEnS_summary.models, "PEnS_ranked_glmm.models.csv")
```

## Assessing the results from the best model

Once we have ranked the models and selected the best, we assessed the
results.

```
PEnS_summary.models <- read.csv("PEnS_ranked_glmm.models.csv")
PEnS_summary.models$X <- NULL


PEnS_best.model <- glmmTMB(
  formula = as.formula(PEnS_summary.models[order(PEnS_summary.models$AICc), ][1,1]),
                           ziformula = ~ 0,
                           family = ordbeta(link = "logit"), 
                           REML=F, 
                           data = data
  )
```

```
## Warning in finalizeTMB(TMBStruc, obj, fit, h, data.tmb.old): Model convergence
## problem; false convergence (8). See vignette('troubleshooting'),
## help('diagnose')
```

Because of model convergence problem, we will follow the
recommendations in glmmTMB vignette.

```
PEnS_best.model <- update(PEnS_best.model, 
                              control=glmmTMBControl(optimizer=optim, optArgs=list(method="BFGS")))
```

The best model contains: 1. Only one explanatory variable:
*maximum depth (m)* 2. The random variable: *catchment* 3.
No zero inflation component

*Random variable* The presence of the random variable suggests
the intercept varies among catchments. The variance is 0.8186 while the
variance standard deviation is 0.9048; therefore, there is indeed an
apparently meaningful variability accross catchments in the baseline of
endemism proportion.

*Dispersion parameter* The dispersion parameter is 32.3, which
suggests low dispersion of the response variable around the predicted
mean values.

*Fixed effects* **Slope**: The slope of the fit
(3.5169) is significantly different from zero (p = 1.09e-06), indicating
a strong positive effect of maximum depth The conclusion is that lakes
with greater maximum depth are associated with higher endemism
proportion, even after controlling for differences between catchments.
The effect is large, positive, and statistically significant.

```
summary(PEnS_best.model)
```

```
##  Family: ordbeta  ( logit )
## Formula:          
## Endemism_proportion ~ scales::rescale(log1p(Maximum_depth_m)) +  
##     (1 | Catchment)
## Data: data
## 
##      AIC      BIC   logLik deviance df.resid 
##     12.1     26.4     -0.1      0.1       73 
## 
## Random effects:
## 
## Conditional model:
##  Groups    Name        Variance Std.Dev.
##  Catchment (Intercept) 0.8186   0.9048  
## Number of obs: 79, groups:  Catchment, 4
## 
## Dispersion parameter for ordbeta family (): 32.3 
## 
## Conditional model:
##                                         Estimate Std. Error z value Pr(>|z|)
## (Intercept)                              -5.4465     0.8138  -6.693 2.19e-11
## scales::rescale(log1p(Maximum_depth_m))   3.5169     0.7214   4.875 1.09e-06
##                                            
## (Intercept)                             ***
## scales::rescale(log1p(Maximum_depth_m)) ***
## ---
## Signif. codes:  0 '***' 0.001 '**' 0.01 '*' 0.05 '.' 0.1 ' ' 1
```

# Model diagnosticos,

For residulas diagnostics and formal assessement of dispersal and
zero inflation, we used DHARMa. Because the model contains only one
variable, there was no need to re-test for multicollinearity

```
library(DHARMa)

# Residual diagnostics:
PEnS_res <- simulateResiduals(fittedModel = PEnS_best.model, n = 10000)
plot(PEnS_res, rank = TRUE)
```

```
# Confirming that our model small residual variation (underdispersed):
testDispersion(PEnS_res)
```

```
## 
##  DHARMa nonparametric dispersion test via sd of residuals fitted vs.
##  simulated
## 
## data:  simulationOutput
## dispersion = 1.1217, p-value = 0.52
## alternative hypothesis: two.sided
```

```
# Checking that model’s handling of zeros is fine.
testZeroInflation(PEnS_res)
```

```
## 
##  DHARMa zero-inflation test via comparison to expected zeros with
##  simulation under H0 = fitted model
## 
## data:  simulationOutput
## ratioObsSim = 1.0022, p-value = 0.9882
## alternative hypothesis: two.sided
```

Making a preliminary plot to visualize the effect of maximum depth on
endemism proportion using *ggefects*.

```
library(ggeffects)
library(ggplot2)

PEnS_predicted_max_depth <- predict_response(PEnS_best.model, terms = "Maximum_depth_m [all]")

ggplot(PEnS_predicted_max_depth, aes(x = x, y = predicted)) +
  geom_line() +
  geom_ribbon(aes(ymin = conf.low, ymax = conf.high), alpha = 0.2)
```

## Running GLMM on Total Native Species Richness (TNSR)

As before, for variable combination generated in the previous step,
we fitted a GLMM with or without random variable (*Catchment*),
but we **didn’t account for zero inflation**.

Again, continuous explanatory variables were first log-transformed
and rescaled to vary in range between 0 and 1.

```
response_var <- "D1.Total_native_Richness"

TNSR_model_list_no_random <- list()
TNSR_model_list_no_random_notation <- list()


   #####################################################################
  ### TNSR MODELS WITHOUT THE RANDOM VARIABLE AND NO ZERO INFLATION ### 
######################################################################

for (i in seq_along(combinations_list)) {
  
  vars <- combinations_list[[i]]
  
  transformed_vars <- sapply(vars, function(var) {
      paste0("scales::rescale(log1p(", var, "))")
    }
  )
  
  formula_str <- paste0(response_var, "~", paste(transformed_vars, collapse = " + "))
  formula_obj <- as.formula(formula_str)
  
  TNSR_model_list_no_random[[i]] <- glmmTMB::glmmTMB(formula = formula_obj, 
                                                ziformula = ~0, 
                                                family = nbinom2(link = "logit"), 
                                                REML = F, 
                                                data = data)
  
  TNSR_model_list_no_random_notation[[i]] <- formula_str
}


   ###################################################################
  ### TNSR MODELS WITH THE RANDOM VARIABLE AND NO ZERO INFLATION ### 
####################################################################

TNSR_model_list_with_random <- list()
TNSR_model_list_with_random_notation <- list()

for (i in seq_along(combinations_list)) {
  
  vars <- combinations_list[[i]]
  
  transformed_vars <- sapply(vars, function(var) {
      paste0("scales::rescale(log1p(", var, "))")
    }
  )
  
  formula_str <- paste0(response_var, "~", paste(transformed_vars, collapse = " + "), " + (1|Catchment)")
  formula_obj <- as.formula(formula_str)
  
  # Initialize flags to track if warnings occur
  warning_occurred <- FALSE
  second_warning_occurred <- FALSE
  
  # Run the model and catch warnings
  TNSR_model_list_with_random[[i]] <- withCallingHandlers({
    fit <- glmmTMB(formula = formula_obj, 
                   ziformula = ~0, 
                   family = nbinom2, 
                   data = data, 
                   REML = F)
  }, warning = function(w) {
    warning_occurred <<- TRUE
    invokeRestart("muffleWarning")
  }
  )
  
  # If a warning occurred, update the model with different control parameters
  if (warning_occurred) {
    TNSR_model_list_with_random[[i]] <- withCallingHandlers({
      updated_fit <- update(TNSR_model_list_with_random[[i]],
                            control = glmmTMBControl(optimizer = optim,
                                                     optArgs = list(method = "BFGS")))
    }, warning = function(w) {
      second_warning_occurred <<- TRUE
      invokeRestart("muffleWarning")
    })
  }
 
  TNSR_model_list_with_random_notation[[i]] <- formula_str
}
```

# Summarizing the models for model selection

First, let’s summarize the results from the models for *without
random variable*.

```
TNSR_summary.models <- as.data.frame(matrix(data=NA, nrow=1, ncol=8))

names(TNSR_summary.models) <- c("formula", 
                                "AIC", 
                                "AICc", 
                                "BIC", 
                                "R2.mar.mumin", 
                                "R2.cond.mumin", 
                                "random_var",
                                "zi")

TNSR_summary.models.no.random <- list()
TNSR_summary.models.with.random <- list()


for (i in seq_along(TNSR_model_list_no_random)){
  
  res <- summary(TNSR_model_list_no_random[[i]])
  
  TNSR_summary.models[,1:8] <- c(
    TNSR_model_list_no_random_notation[[i]], 
    res$AICtab[1],
    AICcmodavg::AICc(TNSR_model_list_no_random[[i]]),
    res$AICtab[2],
    NA, # as.data.frame(MuMIn::r.squaredGLMM(PEnS_model_list_with_random_zi[[i]]))$R2m[1],
    NA, # as.data.frame(MuMIn::r.squaredGLMM(PEnS_model_list_with_random_zi[[i]]))$R2c[1],    
    "no",
    "no"
  )
  
  TNSR_summary.models.no.random[[i]] <- TNSR_summary.models
}
```

Then, the results from the models for *with random
variable*

```
for (i in seq_along(TNSR_model_list_with_random)){
  
  res <- summary(TNSR_model_list_with_random[[i]])
  
  TNSR_summary.models[,1:8] <- c(TNSR_model_list_with_random_notation[[i]], 
                                 res$AICtab[1],
                                 AICcmodavg::AICc(TNSR_model_list_with_random[[i]]),
                                 res$AICtab[2],
                                 as.data.frame(MuMIn::r.squaredGLMM(TNSR_model_list_with_random[[i]]))$R2m[1],
                                 as.data.frame(MuMIn::r.squaredGLMM(TNSR_model_list_with_random[[i]]))$R2c[1],
                                 "yes",
                                 "no"
  )
  
  TNSR_summary.models.with.random[[i]] <- TNSR_summary.models
}
```

## Comparing the models

Best model is selected based on AICc.

```
library(dplyr)

TNSR_data1 <- do.call(bind_rows, TNSR_summary.models.no.random)
TNSR_data2 <- do.call(bind_rows, TNSR_summary.models.with.random)


TNSR_summary.models <- bind_rows(TNSR_data1,TNSR_data2)

TNSR_summary.models$Model <- paste0("Model.", seq_len(nrow(TNSR_summary.models)))

TNSR_summary.models[, c(2:6)] <- lapply(TNSR_summary.models[, c(2:6)], as.numeric)

TNSR_summary.models$Delta_AICc <- TNSR_summary.models$AICc-(min(TNSR_summary.models$AICc, na.rm = T))

# Which is the best model based on AICc?
(TNSR_best_model <- TNSR_summary.models[order(TNSR_summary.models$AICc), ][1,])
```

```
##                                                                                                                                                                                    formula
## 156 D1.Total_native_Richness~scales::rescale(log1p(Surface_area_km2)) + scales::rescale(log1p(Maximum_depth_m)) + scales::rescale(log1p(avg_surface_temp_domischetal2015)) + (1|Catchment)
##          AIC     AICc      BIC R2.mar.mumin R2.cond.mumin random_var zi
## 156 448.6135 449.7802 462.8302    0.5843074     0.7817445        yes no
##         Model Delta_AICc
## 156 Model.156          0
```

```
# Saving the results of the model comparisons
write.csv(TNSR_summary.models, "TNSR_ranked_glmm.models.csv")
```

## Assessing the results from the best model

Once we have ranked the models and selected the best, we assessed the
results.

```
TNSR_summary.models <- read.csv("TNSR_ranked_glmm.models.csv")
TNSR_summary.models$X <- NULL

TNSR_best.model <- glmmTMB(formula =
                             as.formula(TNSR_summary.models[order(TNSR_summary.models$AICc), ][1,1]),
                           ziformula = ~ 0,
                           family = nbinom2, 
                           REML=F, 
                           data = data)
```

```
## Warning in finalizeTMB(TMBStruc, obj, fit, h, data.tmb.old): Model convergence
## problem; false convergence (8). See vignette('troubleshooting'),
## help('diagnose')
```

Because of model convergence problem, we will follow the
recommendations in glmmTMB vignette.

```
TNSR_best.model <- update(TNSR_best.model, 
                              control=glmmTMBControl(optimizer=optim, optArgs=list(method="BFGS")))
```

The best model contains: 1. Three explanatory variables: *Lake
area (km2)*, *maximum depth (m)* and *average surface
temperature (°C)* 2. The random variable: *catchment* 3. No
zero inflation component

*Random variable* The variance is 0.05652 while the variance
standard deviation is 0.2377. This suggests that the catchment-level
variation in total native species richness exists but is relatively
small.

*Dispersion parameter* The dispersion parameter (θ) is 6880. A
larger value of θ indicates that the variance is closer to the mean, and
the data are less overdispersed. In a negative binomial model, the
variance is modeled as: \[
\text{Var}(Y) = \mu + \frac{\mu^2}{\theta}
\] So, a larger θ means less overdispersion (i.e. variance is
closer to the mean).

*Fixed effects* All three predictors are statistically
significant.

```
summary(TNSR_best.model)
```

```
##  Family: nbinom2  ( log )
## Formula:          
## D1.Total_native_Richness ~ scales::rescale(log1p(Surface_area_km2)) +  
##     scales::rescale(log1p(Maximum_depth_m)) + scales::rescale(log1p(avg_surface_temp_domischetal2015)) +  
##     (1 | Catchment)
## Data: data
## 
##      AIC      BIC   logLik deviance df.resid 
##    448.6    462.8   -218.3    436.6       73 
## 
## Random effects:
## 
## Conditional model:
##  Groups    Name        Variance Std.Dev.
##  Catchment (Intercept) 0.05652  0.2377  
## Number of obs: 79, groups:  Catchment, 4
## 
## Dispersion parameter for nbinom2 family (): 6.88e+03 
## 
## Conditional model:
##                                                          Estimate Std. Error
## (Intercept)                                                1.5012     0.2590
## scales::rescale(log1p(Surface_area_km2))                   0.9801     0.2370
## scales::rescale(log1p(Maximum_depth_m))                    0.5902     0.2783
## scales::rescale(log1p(avg_surface_temp_domischetal2015))   0.5860     0.2380
##                                                          z value Pr(>|z|)    
## (Intercept)                                                5.797 6.77e-09 ***
## scales::rescale(log1p(Surface_area_km2))                   4.135 3.55e-05 ***
## scales::rescale(log1p(Maximum_depth_m))                    2.120   0.0340 *  
## scales::rescale(log1p(avg_surface_temp_domischetal2015))   2.463   0.0138 *  
## ---
## Signif. codes:  0 '***' 0.001 '**' 0.01 '*' 0.05 '.' 0.1 ' ' 1
```

## Diagnostics

```
library(DHARMa)

# Residual diagnostics:
TNSR_res <- simulateResiduals(fittedModel = TNSR_best.model, n = 10000)
plot(TNSR_res, rank = TRUE)
```

```
# Confirming that our model small residual variation (underdispersed):
testDispersion(TNSR_res)
```

```
## 
##  DHARMa nonparametric dispersion test via sd of residuals fitted vs.
##  simulated
## 
## data:  simulationOutput
## dispersion = 1.3469, p-value = 0.336
## alternative hypothesis: two.sided
```

```
# Checking that model’s handling of zeros is fine.
testZeroInflation(TNSR_res)
```

```
## 
##  DHARMa zero-inflation test via comparison to expected zeros with
##  simulation under H0 = fitted model
## 
## data:  simulationOutput
## ratioObsSim = 0, p-value = 1
## alternative hypothesis: two.sided
```

Preliminary plot to visualize the fixed effects.

```
library(ggeffects)
library(ggplot2)

TNSR_predicted_area <- predict_response(TNSR_best.model, terms = "Surface_area_km2 [all]")
TNSR_predicted_max_depth <- predict_response(TNSR_best.model, terms = "Maximum_depth_m [all]")
TNSR_predicted_temp <- predict_response(TNSR_best.model, terms = "avg_surface_temp_domischetal2015 [all]")

ggplot(TNSR_predicted_area, aes(x = x, y = predicted)) +
  geom_line() +
  geom_ribbon(aes(ymin = conf.low, ymax = conf.high), alpha = 0.2)
```

```
ggplot(TNSR_predicted_max_depth, aes(x = x, y = predicted)) +
  geom_line() +
  geom_ribbon(aes(ymin = conf.low, ymax = conf.high), alpha = 0.2)
```

```
ggplot(TNSR_predicted_temp, aes(x = x, y = predicted)) +
  geom_line() +
  geom_ribbon(aes(ymin = conf.low, ymax = conf.high), alpha = 0.2)
```

## Running GLMM on toal native salmonid species richness (NSSR)

We used the R package *glmmTMB*

```
citation("glmmTMB")
```

```
## To cite glmmTMB in publications use:
## 
##   Mollie E. Brooks, Kasper Kristensen, Koen J. van Benthem, Arni
##   Magnusson, Casper W. Berg, Anders Nielsen, Hans J. Skaug, Martin
##   Maechler and Benjamin M. Bolker (2017). glmmTMB Balances Speed and
##   Flexibility Among Packages for Zero-inflated Generalized Linear Mixed
##   Modeling. The R Journal, 9(2), 378-400. doi: 10.32614/RJ-2017-066.
## 
## A BibTeX entry for LaTeX users is
## 
##   @Article{,
##     author = {Mollie E. Brooks and Kasper Kristensen and Koen J. {van Benthem} and Arni Magnusson and Casper W. Berg and Anders Nielsen and Hans J. Skaug and Martin Maechler and Benjamin M. Bolker},
##     title = {{glmmTMB} Balances Speed and Flexibility Among Packages for Zero-inflated Generalized Linear Mixed Modeling},
##     year = {2017},
##     journal = {The R Journal},
##     doi = {10.32614/RJ-2017-066},
##     pages = {378--400},
##     volume = {9},
##     number = {2},
##   }
```

We fitted models with or without random variable
(*Catchment*), and with or without accounting for *zero
inflation*.

```
data$Catchment <- as.factor(data$Catchment)
random_var <- 'Catchment'
response_var <- "D1.Native.Salmonidae.richness"

library(glmmTMB)


   ##########################################################################
  ### NSSR MODELS WITHOUT THE RANDOM VARIABLE AND WITHOUT ZERO INFLATION ### 
###########################################################################

NSSR_model_list_no_random <- list()
NSSR_model_list_no_random_notation <- list()

for (i in seq_along(combinations_list)) {
  vars <- combinations_list[[i]]
  
  transformed_vars <- sapply(vars, function(var) {
      paste0("scales::rescale(log1p(", var, "))")
  }
  )
  
  formula_str <- paste0(response_var, "~", paste(transformed_vars, collapse = " + "))  
  formula_obj <- as.formula(formula_str)
  
  NSSR_model_list_no_random[[i]] <- glmmTMB(formula = formula_obj, 
                                       ziformula = ~0, 
                                       family = nbinom2, 
                                       data = data, 
                                       REML = F)
  NSSR_model_list_no_random_notation[[i]] <- formula_str
}


   #######################################################################
  ### NSSR MODELS WITHOUT THE RANDOM VARIABLE AND WITH ZERO INFLATION ### 
########################################################################


NSSR_model_list_no_random_zi <- list()
NSSR_model_list_no_random_notation_zi <- list()

for (i in seq_along(combinations_list)) {
  vars <- combinations_list[[i]]
  
  transformed_vars <- sapply(vars, function(var) {
      paste0("scales::rescale(log1p(", var, "))")
    }
  )
  
  formula_str <- paste0(response_var, "~", paste(transformed_vars, collapse = " + "))  
  formula_obj <- as.formula(formula_str)
  
  NSSR_model_list_no_random_zi[[i]] <- glmmTMB(formula = formula_obj, 
                                               ziformula = ~1, 
                                               family = nbinom2, 
                                               data = data, 
                                               REML = F)
  
  NSSR_model_list_no_random_notation_zi[[i]] <- formula_str
}


   ###################################################################
  ### NSSR MODELS WITH THE RANDOM VARIABLE AND NO ZERO INFLATION ### 
####################################################################

NSSR_model_list_with_random <- list()
NSSR_model_list_with_random_notation <- list()

for (i in seq_along(combinations_list)) {
  vars <- combinations_list[[i]]
  
  transformed_vars <- sapply(vars, function(var) {
      paste0("scales::rescale(log1p(", var, "))")
    }
  )
  
  formula_str <- paste0(response_var, "~", paste(transformed_vars, collapse = " + "), " + (1|Catchment)")  
  formula_obj <- as.formula(formula_str)
  
  # Initialize flags to track if warnings occur
  warning_occurred <- FALSE
  second_warning_occurred <- FALSE
  
  # Run the model and catch warnings
  NSSR_model_list_with_random[[i]] <- withCallingHandlers({
    fit <- glmmTMB(formula = formula_obj, 
                   ziformula = ~0, 
                   family = nbinom2, 
                   data = data, REML = F)
    fit
  }, warning = function(w) {
    warning_occurred <<- TRUE
    invokeRestart("muffleWarning")
  })
  
  # If a warning occurred, update the model with different control parameters
  if (warning_occurred) {
    NSSR_model_list_with_random[[i]] <- withCallingHandlers({
      updated_fit <- update(NSSR_model_list_with_random[[i]],
                            control = glmmTMBControl(optimizer = optim,
                                                     optArgs = list(method = "BFGS")))
      updated_fit
    }, warning = function(w) {
      second_warning_occurred <<- TRUE
      invokeRestart("muffleWarning")
    })
  }
  
  # If a warning also occurred during the first update, apply the second update
  if (second_warning_occurred) {
    NSSR_model_list_with_random[[i]] <- update(NSSR_model_list_with_random[[i]], ziformula = ~(1|Catchment))
  }
  
  NSSR_model_list_with_random_notation[[i]] <- formula_str
}

   ####################################################################
  ### NSSR MODELS WITH THE RANDOM VARIABLE AND WITH ZERO INFLATION ### 
#####################################################################

NSSR_model_list_with_random_zi <- list()
NSSR_model_list_with_random_notation_zi <- list()

for (i in seq_along(combinations_list)) {
  
  vars <- combinations_list[[i]]
  
  transformed_vars <- sapply(vars, function(var) {
      paste0("scales::rescale(log1p(", var, "))")
    }
  )
  
  formula_str <- paste0(response_var, "~", paste(transformed_vars, collapse = " + "), " + (1|Catchment)")  
  formula_obj <- as.formula(formula_str)
  
  # Initialize flags to track if warnings occur
  warning_occurred <- FALSE
  second_warning_occurred <- FALSE
  
  # Run the model and catch warnings
  NSSR_model_list_with_random_zi[[i]] <- withCallingHandlers({
    fit <- glmmTMB(formula = formula_obj, 
                   ziformula = ~1, 
                   family = nbinom2, 
                   data = data, 
                   REML = F)
    fit
  }, warning = function(w) {
    warning_occurred <<- TRUE
    invokeRestart("muffleWarning")
  })
  
  # If a warning occurred, update the model with different control parameters
  if (warning_occurred) {
    NSSR_model_list_with_random_zi[[i]] <- withCallingHandlers({
      updated_fit <- update(NSSR_model_list_with_random_zi[[i]],
                            control = glmmTMBControl(optimizer = optim,
                                                     optArgs = list(method = "BFGS")))
      updated_fit
    }, warning = function(w) {
      second_warning_occurred <<- TRUE
      invokeRestart("muffleWarning")
    })
  }
  
  # If a warning also occurred during the first update, apply the second update
  if (second_warning_occurred) {
    NSSR_model_list_with_random_zi[[i]] <- update(NSSR_model_list_with_random_zi[[i]], ziformula = ~(1|Catchment))
  }
  
  NSSR_model_list_with_random_notation_zi[[i]] <- formula_str
}
```

# Summarizing the models for model selection

Results from the models for *without random variable* and
*without zero inflation*

```
NSSR_summary.models <- as.data.frame(matrix(data=NA, nrow=1, ncol=8))

names(NSSR_summary.models) <- c("formula", 
                                "AIC", 
                                "AICc", 
                                "BIC", 
                                "R2.mar.mumin", 
                                "R2.cond.mumin", 
                                "random_var",
                                "zi")

NSSR_summary.models.no.random <- list()
NSSR_summary.models.no.random_zi <- list()
NSSR_summary.models.with.random <- list()
NSSR_summary.models.with.random_zi <- list()


for (i in seq_along(NSSR_model_list_no_random)){
  
  res <- summary(NSSR_model_list_no_random[[i]])
  
  NSSR_summary.models[,1:8] <- c(
    NSSR_model_list_no_random_notation[[i]], 
    res$AICtab[1],
    AICcmodavg::AICc(NSSR_model_list_no_random[[i]]),
    res$AICtab[2],
    as.data.frame(MuMIn::r.squaredGLMM(NSSR_model_list_no_random[[i]]))$R2m[1],
    NA,
    "no",
    "no"
  )
  
  NSSR_summary.models.no.random[[i]] <- NSSR_summary.models
}
```

Results from the models for *without random variable* and
*with zero inflation*

```
for (i in seq_along(NSSR_model_list_no_random_zi)){
  
  res <- summary(NSSR_model_list_no_random_zi[[i]])
  
  NSSR_summary.models[,1:8] <- c(
    NSSR_model_list_no_random_notation_zi[[i]], 
    res$AICtab[1],
    AICcmodavg::AICc(NSSR_model_list_no_random_zi[[i]]),
    res$AICtab[2],
    as.data.frame(MuMIn::r.squaredGLMM(NSSR_model_list_no_random_zi[[i]]))$R2m[1],
    NA,
    "no",
    "yes"
  )
  
  NSSR_summary.models.no.random_zi[[i]] <- NSSR_summary.models
}
```

```
## Warning in r.squaredGLMM.glmmTMB(NSSR_model_list_no_random_zi[[i]]): the
## effects of zero-inflation and dispersion model are ignored
```

```
## Warning: the null model is correct only if all variables used by the original
## model remain unchanged.
```

```
## Warning in r.squaredGLMM.glmmTMB(NSSR_model_list_no_random_zi[[i]]): the
## effects of zero-inflation and dispersion model are ignored
```

```
## Warning: the null model is correct only if all variables used by the original
## model remain unchanged.
```

```
## Warning in r.squaredGLMM.glmmTMB(NSSR_model_list_no_random_zi[[i]]): the
## effects of zero-inflation and dispersion model are ignored
```

```
## Warning: the null model is correct only if all variables used by the original
## model remain unchanged.
```

```
## Warning in r.squaredGLMM.glmmTMB(NSSR_model_list_no_random_zi[[i]]): the
## effects of zero-inflation and dispersion model are ignored
```

```
## Warning: the null model is correct only if all variables used by the original
## model remain unchanged.
```

```
## Warning in r.squaredGLMM.glmmTMB(NSSR_model_list_no_random_zi[[i]]): the
## effects of zero-inflation and dispersion model are ignored
```

```
## Warning: the null model is correct only if all variables used by the original
## model remain unchanged.
```

```
## Warning in r.squaredGLMM.glmmTMB(NSSR_model_list_no_random_zi[[i]]): the
## effects of zero-inflation and dispersion model are ignored
```

```
## Warning: the null model is correct only if all variables used by the original
## model remain unchanged.
```

```
## Warning in r.squaredGLMM.glmmTMB(NSSR_model_list_no_random_zi[[i]]): the
## effects of zero-inflation and dispersion model are ignored
```

```
## Warning: the null model is correct only if all variables used by the original
## model remain unchanged.
```

```
## Warning in r.squaredGLMM.glmmTMB(NSSR_model_list_no_random_zi[[i]]): the
## effects of zero-inflation and dispersion model are ignored
```

```
## Warning: the null model is correct only if all variables used by the original
## model remain unchanged.
```

```
## Warning in r.squaredGLMM.glmmTMB(NSSR_model_list_no_random_zi[[i]]): the
## effects of zero-inflation and dispersion model are ignored
```

```
## Warning: the null model is correct only if all variables used by the original
## model remain unchanged.
```

```
## Warning in r.squaredGLMM.glmmTMB(NSSR_model_list_no_random_zi[[i]]): the
## effects of zero-inflation and dispersion model are ignored
```

```
## Warning: the null model is correct only if all variables used by the original
## model remain unchanged.
```

```
## Warning in r.squaredGLMM.glmmTMB(NSSR_model_list_no_random_zi[[i]]): the
## effects of zero-inflation and dispersion model are ignored
```

```
## Warning: the null model is correct only if all variables used by the original
## model remain unchanged.
```

```
## Warning in r.squaredGLMM.glmmTMB(NSSR_model_list_no_random_zi[[i]]): the
## effects of zero-inflation and dispersion model are ignored
```

```
## Warning: the null model is correct only if all variables used by the original
## model remain unchanged.
```

```
## Warning in r.squaredGLMM.glmmTMB(NSSR_model_list_no_random_zi[[i]]): the
## effects of zero-inflation and dispersion model are ignored
```

```
## Warning: the null model is correct only if all variables used by the original
## model remain unchanged.
```

```
## Warning in r.squaredGLMM.glmmTMB(NSSR_model_list_no_random_zi[[i]]): the
## effects of zero-inflation and dispersion model are ignored
```

```
## Warning: the null model is correct only if all variables used by the original
## model remain unchanged.
```

```
## Warning in r.squaredGLMM.glmmTMB(NSSR_model_list_no_random_zi[[i]]): the
## effects of zero-inflation and dispersion model are ignored
```

```
## Warning: the null model is correct only if all variables used by the original
## model remain unchanged.
```

```
## Warning in r.squaredGLMM.glmmTMB(NSSR_model_list_no_random_zi[[i]]): the
## effects of zero-inflation and dispersion model are ignored
```

```
## Warning: the null model is correct only if all variables used by the original
## model remain unchanged.
```

```
## Warning in r.squaredGLMM.glmmTMB(NSSR_model_list_no_random_zi[[i]]): the
## effects of zero-inflation and dispersion model are ignored
```

```
## Warning: the null model is correct only if all variables used by the original
## model remain unchanged.
```

```
## Warning in r.squaredGLMM.glmmTMB(NSSR_model_list_no_random_zi[[i]]): the
## effects of zero-inflation and dispersion model are ignored
```

```
## Warning: the null model is correct only if all variables used by the original
## model remain unchanged.
```

```
## Warning in r.squaredGLMM.glmmTMB(NSSR_model_list_no_random_zi[[i]]): the
## effects of zero-inflation and dispersion model are ignored
```

```
## Warning: the null model is correct only if all variables used by the original
## model remain unchanged.
```

```
## Warning in r.squaredGLMM.glmmTMB(NSSR_model_list_no_random_zi[[i]]): the
## effects of zero-inflation and dispersion model are ignored
```

```
## Warning: the null model is correct only if all variables used by the original
## model remain unchanged.
```

```
## Warning in r.squaredGLMM.glmmTMB(NSSR_model_list_no_random_zi[[i]]): the
## effects of zero-inflation and dispersion model are ignored
```

```
## Warning: the null model is correct only if all variables used by the original
## model remain unchanged.
```

```
## Warning in r.squaredGLMM.glmmTMB(NSSR_model_list_no_random_zi[[i]]): the
## effects of zero-inflation and dispersion model are ignored
```

```
## Warning: the null model is correct only if all variables used by the original
## model remain unchanged.
```

```
## Warning in r.squaredGLMM.glmmTMB(NSSR_model_list_no_random_zi[[i]]): the
## effects of zero-inflation and dispersion model are ignored
```

```
## Warning: the null model is correct only if all variables used by the original
## model remain unchanged.
```

```
## Warning in r.squaredGLMM.glmmTMB(NSSR_model_list_no_random_zi[[i]]): the
## effects of zero-inflation and dispersion model are ignored
```

```
## Warning: the null model is correct only if all variables used by the original
## model remain unchanged.
```

```
## Warning in r.squaredGLMM.glmmTMB(NSSR_model_list_no_random_zi[[i]]): the
## effects of zero-inflation and dispersion model are ignored
```

```
## Warning: the null model is correct only if all variables used by the original
## model remain unchanged.
```

```
## Warning in r.squaredGLMM.glmmTMB(NSSR_model_list_no_random_zi[[i]]): the
## effects of zero-inflation and dispersion model are ignored
```

```
## Warning: the null model is correct only if all variables used by the original
## model remain unchanged.
```

```
## Warning in r.squaredGLMM.glmmTMB(NSSR_model_list_no_random_zi[[i]]): the
## effects of zero-inflation and dispersion model are ignored
```

```
## Warning: the null model is correct only if all variables used by the original
## model remain unchanged.
```

```
## Warning in r.squaredGLMM.glmmTMB(NSSR_model_list_no_random_zi[[i]]): the
## effects of zero-inflation and dispersion model are ignored
```

```
## Warning: the null model is correct only if all variables used by the original
## model remain unchanged.
```

```
## Warning in r.squaredGLMM.glmmTMB(NSSR_model_list_no_random_zi[[i]]): the
## effects of zero-inflation and dispersion model are ignored
```

```
## Warning: the null model is correct only if all variables used by the original
## model remain unchanged.
```

```
## Warning in r.squaredGLMM.glmmTMB(NSSR_model_list_no_random_zi[[i]]): the
## effects of zero-inflation and dispersion model are ignored
```

```
## Warning: the null model is correct only if all variables used by the original
## model remain unchanged.
```

```
## Warning in r.squaredGLMM.glmmTMB(NSSR_model_list_no_random_zi[[i]]): the
## effects of zero-inflation and dispersion model are ignored
```

```
## Warning: the null model is correct only if all variables used by the original
## model remain unchanged.
```

```
## Warning in r.squaredGLMM.glmmTMB(NSSR_model_list_no_random_zi[[i]]): the
## effects of zero-inflation and dispersion model are ignored
```

```
## Warning: the null model is correct only if all variables used by the original
## model remain unchanged.
```

```
## Warning in r.squaredGLMM.glmmTMB(NSSR_model_list_no_random_zi[[i]]): the
## effects of zero-inflation and dispersion model are ignored
```

```
## Warning: the null model is correct only if all variables used by the original
## model remain unchanged.
```

```
## Warning in r.squaredGLMM.glmmTMB(NSSR_model_list_no_random_zi[[i]]): the
## effects of zero-inflation and dispersion model are ignored
```

```
## Warning: the null model is correct only if all variables used by the original
## model remain unchanged.
```

```
## Warning in r.squaredGLMM.glmmTMB(NSSR_model_list_no_random_zi[[i]]): the
## effects of zero-inflation and dispersion model are ignored
```

```
## Warning: the null model is correct only if all variables used by the original
## model remain unchanged.
```

```
## Warning in r.squaredGLMM.glmmTMB(NSSR_model_list_no_random_zi[[i]]): the
## effects of zero-inflation and dispersion model are ignored
```

```
## Warning: the null model is correct only if all variables used by the original
## model remain unchanged.
```

```
## Warning in r.squaredGLMM.glmmTMB(NSSR_model_list_no_random_zi[[i]]): the
## effects of zero-inflation and dispersion model are ignored
```

```
## Warning: the null model is correct only if all variables used by the original
## model remain unchanged.
```

```
## Warning in r.squaredGLMM.glmmTMB(NSSR_model_list_no_random_zi[[i]]): the
## effects of zero-inflation and dispersion model are ignored
```

```
## Warning: the null model is correct only if all variables used by the original
## model remain unchanged.
```

```
## Warning in r.squaredGLMM.glmmTMB(NSSR_model_list_no_random_zi[[i]]): the
## effects of zero-inflation and dispersion model are ignored
```

```
## Warning: the null model is correct only if all variables used by the original
## model remain unchanged.
```

```
## Warning in r.squaredGLMM.glmmTMB(NSSR_model_list_no_random_zi[[i]]): the
## effects of zero-inflation and dispersion model are ignored
```

```
## Warning: the null model is correct only if all variables used by the original
## model remain unchanged.
```

```
## Warning in r.squaredGLMM.glmmTMB(NSSR_model_list_no_random_zi[[i]]): the
## effects of zero-inflation and dispersion model are ignored
```

```
## Warning: the null model is correct only if all variables used by the original
## model remain unchanged.
```

```
## Warning in r.squaredGLMM.glmmTMB(NSSR_model_list_no_random_zi[[i]]): the
## effects of zero-inflation and dispersion model are ignored
```

```
## Warning: the null model is correct only if all variables used by the original
## model remain unchanged.
```

```
## Warning in r.squaredGLMM.glmmTMB(NSSR_model_list_no_random_zi[[i]]): the
## effects of zero-inflation and dispersion model are ignored
```

```
## Warning: the null model is correct only if all variables used by the original
## model remain unchanged.
```

```
## Warning in r.squaredGLMM.glmmTMB(NSSR_model_list_no_random_zi[[i]]): the
## effects of zero-inflation and dispersion model are ignored
```

```
## Warning: the null model is correct only if all variables used by the original
## model remain unchanged.
```

```
## Warning in r.squaredGLMM.glmmTMB(NSSR_model_list_no_random_zi[[i]]): the
## effects of zero-inflation and dispersion model are ignored
```

```
## Warning: the null model is correct only if all variables used by the original
## model remain unchanged.
```

```
## Warning in r.squaredGLMM.glmmTMB(NSSR_model_list_no_random_zi[[i]]): the
## effects of zero-inflation and dispersion model are ignored
```

```
## Warning: the null model is correct only if all variables used by the original
## model remain unchanged.
```

```
## Warning in r.squaredGLMM.glmmTMB(NSSR_model_list_no_random_zi[[i]]): the
## effects of zero-inflation and dispersion model are ignored
```

```
## Warning: the null model is correct only if all variables used by the original
## model remain unchanged.
```

```
## Warning in r.squaredGLMM.glmmTMB(NSSR_model_list_no_random_zi[[i]]): the
## effects of zero-inflation and dispersion model are ignored
```

```
## Warning: the null model is correct only if all variables used by the original
## model remain unchanged.
```

```
## Warning in r.squaredGLMM.glmmTMB(NSSR_model_list_no_random_zi[[i]]): the
## effects of zero-inflation and dispersion model are ignored
```

```
## Warning: the null model is correct only if all variables used by the original
## model remain unchanged.
```

```
## Warning in r.squaredGLMM.glmmTMB(NSSR_model_list_no_random_zi[[i]]): the
## effects of zero-inflation and dispersion model are ignored
```

```
## Warning: the null model is correct only if all variables used by the original
## model remain unchanged.
```

```
## Warning in r.squaredGLMM.glmmTMB(NSSR_model_list_no_random_zi[[i]]): the
## effects of zero-inflation and dispersion model are ignored
```

```
## Warning: the null model is correct only if all variables used by the original
## model remain unchanged.
```

```
## Warning in r.squaredGLMM.glmmTMB(NSSR_model_list_no_random_zi[[i]]): the
## effects of zero-inflation and dispersion model are ignored
```

```
## Warning: the null model is correct only if all variables used by the original
## model remain unchanged.
```

```
## Warning in r.squaredGLMM.glmmTMB(NSSR_model_list_no_random_zi[[i]]): the
## effects of zero-inflation and dispersion model are ignored
```

```
## Warning: the null model is correct only if all variables used by the original
## model remain unchanged.
```

```
## Warning in r.squaredGLMM.glmmTMB(NSSR_model_list_no_random_zi[[i]]): the
## effects of zero-inflation and dispersion model are ignored
```

```
## Warning: the null model is correct only if all variables used by the original
## model remain unchanged.
```

```
## Warning in r.squaredGLMM.glmmTMB(NSSR_model_list_no_random_zi[[i]]): the
## effects of zero-inflation and dispersion model are ignored
```

```
## Warning: the null model is correct only if all variables used by the original
## model remain unchanged.
```

```
## Warning in r.squaredGLMM.glmmTMB(NSSR_model_list_no_random_zi[[i]]): the
## effects of zero-inflation and dispersion model are ignored
```

```
## Warning: the null model is correct only if all variables used by the original
## model remain unchanged.
```

```
## Warning in r.squaredGLMM.glmmTMB(NSSR_model_list_no_random_zi[[i]]): the
## effects of zero-inflation and dispersion model are ignored
```

```
## Warning: the null model is correct only if all variables used by the original
## model remain unchanged.
```

```
## Warning in r.squaredGLMM.glmmTMB(NSSR_model_list_no_random_zi[[i]]): the
## effects of zero-inflation and dispersion model are ignored
```

```
## Warning: the null model is correct only if all variables used by the original
## model remain unchanged.
```

```
## Warning in r.squaredGLMM.glmmTMB(NSSR_model_list_no_random_zi[[i]]): the
## effects of zero-inflation and dispersion model are ignored
```

```
## Warning: the null model is correct only if all variables used by the original
## model remain unchanged.
```

```
## Warning in r.squaredGLMM.glmmTMB(NSSR_model_list_no_random_zi[[i]]): the
## effects of zero-inflation and dispersion model are ignored
```

```
## Warning: the null model is correct only if all variables used by the original
## model remain unchanged.
```

```
## Warning in r.squaredGLMM.glmmTMB(NSSR_model_list_no_random_zi[[i]]): the
## effects of zero-inflation and dispersion model are ignored
```

```
## Warning: the null model is correct only if all variables used by the original
## model remain unchanged.
```

```
## Warning in r.squaredGLMM.glmmTMB(NSSR_model_list_no_random_zi[[i]]): the
## effects of zero-inflation and dispersion model are ignored
```

```
## Warning: the null model is correct only if all variables used by the original
## model remain unchanged.
```

```
## Warning in r.squaredGLMM.glmmTMB(NSSR_model_list_no_random_zi[[i]]): the
## effects of zero-inflation and dispersion model are ignored
```

```
## Warning: the null model is correct only if all variables used by the original
## model remain unchanged.
```

```
## Warning in r.squaredGLMM.glmmTMB(NSSR_model_list_no_random_zi[[i]]): the
## effects of zero-inflation and dispersion model are ignored
```

```
## Warning: the null model is correct only if all variables used by the original
## model remain unchanged.
```

```
## Warning in r.squaredGLMM.glmmTMB(NSSR_model_list_no_random_zi[[i]]): the
## effects of zero-inflation and dispersion model are ignored
```

```
## Warning: the null model is correct only if all variables used by the original
## model remain unchanged.
```

```
## Warning in r.squaredGLMM.glmmTMB(NSSR_model_list_no_random_zi[[i]]): the
## effects of zero-inflation and dispersion model are ignored
```

```
## Warning: the null model is correct only if all variables used by the original
## model remain unchanged.
```

```
## Warning in r.squaredGLMM.glmmTMB(NSSR_model_list_no_random_zi[[i]]): the
## effects of zero-inflation and dispersion model are ignored
```

```
## Warning: the null model is correct only if all variables used by the original
## model remain unchanged.
```

```
## Warning in r.squaredGLMM.glmmTMB(NSSR_model_list_no_random_zi[[i]]): the
## effects of zero-inflation and dispersion model are ignored
```

```
## Warning: the null model is correct only if all variables used by the original
## model remain unchanged.
```

```
## Warning in r.squaredGLMM.glmmTMB(NSSR_model_list_no_random_zi[[i]]): the
## effects of zero-inflation and dispersion model are ignored
```

```
## Warning: the null model is correct only if all variables used by the original
## model remain unchanged.
```

```
## Warning in r.squaredGLMM.glmmTMB(NSSR_model_list_no_random_zi[[i]]): the
## effects of zero-inflation and dispersion model are ignored
```

```
## Warning: the null model is correct only if all variables used by the original
## model remain unchanged.
```

```
## Warning in r.squaredGLMM.glmmTMB(NSSR_model_list_no_random_zi[[i]]): the
## effects of zero-inflation and dispersion model are ignored
```

```
## Warning: the null model is correct only if all variables used by the original
## model remain unchanged.
```

```
## Warning in r.squaredGLMM.glmmTMB(NSSR_model_list_no_random_zi[[i]]): the
## effects of zero-inflation and dispersion model are ignored
```

```
## Warning: the null model is correct only if all variables used by the original
## model remain unchanged.
```

```
## Warning in r.squaredGLMM.glmmTMB(NSSR_model_list_no_random_zi[[i]]): the
## effects of zero-inflation and dispersion model are ignored
```

```
## Warning: the null model is correct only if all variables used by the original
## model remain unchanged.
```

```
## Warning in r.squaredGLMM.glmmTMB(NSSR_model_list_no_random_zi[[i]]): the
## effects of zero-inflation and dispersion model are ignored
```

```
## Warning: the null model is correct only if all variables used by the original
## model remain unchanged.
```

```
## Warning in r.squaredGLMM.glmmTMB(NSSR_model_list_no_random_zi[[i]]): the
## effects of zero-inflation and dispersion model are ignored
```

```
## Warning: the null model is correct only if all variables used by the original
## model remain unchanged.
```

```
## Warning in r.squaredGLMM.glmmTMB(NSSR_model_list_no_random_zi[[i]]): the
## effects of zero-inflation and dispersion model are ignored
```

```
## Warning: the null model is correct only if all variables used by the original
## model remain unchanged.
```

```
## Warning in r.squaredGLMM.glmmTMB(NSSR_model_list_no_random_zi[[i]]): the
## effects of zero-inflation and dispersion model are ignored
```

```
## Warning: the null model is correct only if all variables used by the original
## model remain unchanged.
```

```
## Warning in r.squaredGLMM.glmmTMB(NSSR_model_list_no_random_zi[[i]]): the
## effects of zero-inflation and dispersion model are ignored
```

```
## Warning: the null model is correct only if all variables used by the original
## model remain unchanged.
```

```
## Warning in r.squaredGLMM.glmmTMB(NSSR_model_list_no_random_zi[[i]]): the
## effects of zero-inflation and dispersion model are ignored
```

```
## Warning: the null model is correct only if all variables used by the original
## model remain unchanged.
```

```
## Warning in r.squaredGLMM.glmmTMB(NSSR_model_list_no_random_zi[[i]]): the
## effects of zero-inflation and dispersion model are ignored
```

```
## Warning: the null model is correct only if all variables used by the original
## model remain unchanged.
```

```
## Warning in r.squaredGLMM.glmmTMB(NSSR_model_list_no_random_zi[[i]]): the
## effects of zero-inflation and dispersion model are ignored
```

```
## Warning: the null model is correct only if all variables used by the original
## model remain unchanged.
```

```
## Warning in r.squaredGLMM.glmmTMB(NSSR_model_list_no_random_zi[[i]]): the
## effects of zero-inflation and dispersion model are ignored
```

```
## Warning: the null model is correct only if all variables used by the original
## model remain unchanged.
```

```
## Warning in r.squaredGLMM.glmmTMB(NSSR_model_list_no_random_zi[[i]]): the
## effects of zero-inflation and dispersion model are ignored
```

```
## Warning: the null model is correct only if all variables used by the original
## model remain unchanged.
```

```
## Warning in r.squaredGLMM.glmmTMB(NSSR_model_list_no_random_zi[[i]]): the
## effects of zero-inflation and dispersion model are ignored
```

```
## Warning: the null model is correct only if all variables used by the original
## model remain unchanged.
```

```
## Warning in r.squaredGLMM.glmmTMB(NSSR_model_list_no_random_zi[[i]]): the
## effects of zero-inflation and dispersion model are ignored
```

```
## Warning: the null model is correct only if all variables used by the original
## model remain unchanged.
```

```
## Warning in r.squaredGLMM.glmmTMB(NSSR_model_list_no_random_zi[[i]]): the
## effects of zero-inflation and dispersion model are ignored
```

```
## Warning: the null model is correct only if all variables used by the original
## model remain unchanged.
```

```
## Warning in r.squaredGLMM.glmmTMB(NSSR_model_list_no_random_zi[[i]]): the
## effects of zero-inflation and dispersion model are ignored
```

```
## Warning: the null model is correct only if all variables used by the original
## model remain unchanged.
```

```
## Warning in r.squaredGLMM.glmmTMB(NSSR_model_list_no_random_zi[[i]]): the
## effects of zero-inflation and dispersion model are ignored
```

```
## Warning: the null model is correct only if all variables used by the original
## model remain unchanged.
```

```
## Warning in r.squaredGLMM.glmmTMB(NSSR_model_list_no_random_zi[[i]]): the
## effects of zero-inflation and dispersion model are ignored
```

```
## Warning: the null model is correct only if all variables used by the original
## model remain unchanged.
```

```
## Warning in r.squaredGLMM.glmmTMB(NSSR_model_list_no_random_zi[[i]]): the
## effects of zero-inflation and dispersion model are ignored
```

```
## Warning: the null model is correct only if all variables used by the original
## model remain unchanged.
```

```
## Warning in r.squaredGLMM.glmmTMB(NSSR_model_list_no_random_zi[[i]]): the
## effects of zero-inflation and dispersion model are ignored
```

```
## Warning: the null model is correct only if all variables used by the original
## model remain unchanged.
```

```
## Warning in r.squaredGLMM.glmmTMB(NSSR_model_list_no_random_zi[[i]]): the
## effects of zero-inflation and dispersion model are ignored
```

```
## Warning: the null model is correct only if all variables used by the original
## model remain unchanged.
```

```
## Warning in r.squaredGLMM.glmmTMB(NSSR_model_list_no_random_zi[[i]]): the
## effects of zero-inflation and dispersion model are ignored
```

```
## Warning: the null model is correct only if all variables used by the original
## model remain unchanged.
```

```
## Warning in r.squaredGLMM.glmmTMB(NSSR_model_list_no_random_zi[[i]]): the
## effects of zero-inflation and dispersion model are ignored
```

```
## Warning: the null model is correct only if all variables used by the original
## model remain unchanged.
```

```
## Warning in r.squaredGLMM.glmmTMB(NSSR_model_list_no_random_zi[[i]]): the
## effects of zero-inflation and dispersion model are ignored
```

```
## Warning: the null model is correct only if all variables used by the original
## model remain unchanged.
```

```
## Warning in r.squaredGLMM.glmmTMB(NSSR_model_list_no_random_zi[[i]]): the
## effects of zero-inflation and dispersion model are ignored
```

```
## Warning: the null model is correct only if all variables used by the original
## model remain unchanged.
```

```
## Warning in r.squaredGLMM.glmmTMB(NSSR_model_list_no_random_zi[[i]]): the
## effects of zero-inflation and dispersion model are ignored
```

```
## Warning: the null model is correct only if all variables used by the original
## model remain unchanged.
```

```
## Warning in r.squaredGLMM.glmmTMB(NSSR_model_list_no_random_zi[[i]]): the
## effects of zero-inflation and dispersion model are ignored
```

```
## Warning: the null model is correct only if all variables used by the original
## model remain unchanged.
```

```
## Warning in r.squaredGLMM.glmmTMB(NSSR_model_list_no_random_zi[[i]]): the
## effects of zero-inflation and dispersion model are ignored
```

```
## Warning: the null model is correct only if all variables used by the original
## model remain unchanged.
```

```
## Warning in r.squaredGLMM.glmmTMB(NSSR_model_list_no_random_zi[[i]]): the
## effects of zero-inflation and dispersion model are ignored
```

```
## Warning: the null model is correct only if all variables used by the original
## model remain unchanged.
```

```
## Warning in r.squaredGLMM.glmmTMB(NSSR_model_list_no_random_zi[[i]]): the
## effects of zero-inflation and dispersion model are ignored
```

```
## Warning: the null model is correct only if all variables used by the original
## model remain unchanged.
```

```
## Warning in r.squaredGLMM.glmmTMB(NSSR_model_list_no_random_zi[[i]]): the
## effects of zero-inflation and dispersion model are ignored
```

```
## Warning: the null model is correct only if all variables used by the original
## model remain unchanged.
```

```
## Warning in r.squaredGLMM.glmmTMB(NSSR_model_list_no_random_zi[[i]]): the
## effects of zero-inflation and dispersion model are ignored
```

```
## Warning: the null model is correct only if all variables used by the original
## model remain unchanged.
```

```
## Warning in r.squaredGLMM.glmmTMB(NSSR_model_list_no_random_zi[[i]]): the
## effects of zero-inflation and dispersion model are ignored
```

```
## Warning: the null model is correct only if all variables used by the original
## model remain unchanged.
```

```
## Warning in r.squaredGLMM.glmmTMB(NSSR_model_list_no_random_zi[[i]]): the
## effects of zero-inflation and dispersion model are ignored
```

```
## Warning: the null model is correct only if all variables used by the original
## model remain unchanged.
```

```
## Warning in r.squaredGLMM.glmmTMB(NSSR_model_list_no_random_zi[[i]]): the
## effects of zero-inflation and dispersion model are ignored
```

```
## Warning: the null model is correct only if all variables used by the original
## model remain unchanged.
```

```
## Warning in r.squaredGLMM.glmmTMB(NSSR_model_list_no_random_zi[[i]]): the
## effects of zero-inflation and dispersion model are ignored
```

```
## Warning: the null model is correct only if all variables used by the original
## model remain unchanged.
```

```
## Warning in r.squaredGLMM.glmmTMB(NSSR_model_list_no_random_zi[[i]]): the
## effects of zero-inflation and dispersion model are ignored
```

```
## Warning: the null model is correct only if all variables used by the original
## model remain unchanged.
```

```
## Warning in r.squaredGLMM.glmmTMB(NSSR_model_list_no_random_zi[[i]]): the
## effects of zero-inflation and dispersion model are ignored
```

```
## Warning: the null model is correct only if all variables used by the original
## model remain unchanged.
```

```
## Warning in r.squaredGLMM.glmmTMB(NSSR_model_list_no_random_zi[[i]]): the
## effects of zero-inflation and dispersion model are ignored
```

```
## Warning: the null model is correct only if all variables used by the original
## model remain unchanged.
```

```
## Warning in r.squaredGLMM.glmmTMB(NSSR_model_list_no_random_zi[[i]]): the
## effects of zero-inflation and dispersion model are ignored
```

```
## Warning: the null model is correct only if all variables used by the original
## model remain unchanged.
```

```
## Warning in r.squaredGLMM.glmmTMB(NSSR_model_list_no_random_zi[[i]]): the
## effects of zero-inflation and dispersion model are ignored
```

```
## Warning: the null model is correct only if all variables used by the original
## model remain unchanged.
```

```
## Warning in r.squaredGLMM.glmmTMB(NSSR_model_list_no_random_zi[[i]]): the
## effects of zero-inflation and dispersion model are ignored
```

```
## Warning: the null model is correct only if all variables used by the original
## model remain unchanged.
```

```
## Warning in r.squaredGLMM.glmmTMB(NSSR_model_list_no_random_zi[[i]]): the
## effects of zero-inflation and dispersion model are ignored
```

```
## Warning: the null model is correct only if all variables used by the original
## model remain unchanged.
```

```
## Warning in r.squaredGLMM.glmmTMB(NSSR_model_list_no_random_zi[[i]]): the
## effects of zero-inflation and dispersion model are ignored
```

```
## Warning: the null model is correct only if all variables used by the original
## model remain unchanged.
```

```
## Warning in r.squaredGLMM.glmmTMB(NSSR_model_list_no_random_zi[[i]]): the
## effects of zero-inflation and dispersion model are ignored
```

```
## Warning: the null model is correct only if all variables used by the original
## model remain unchanged.
```

```
## Warning in r.squaredGLMM.glmmTMB(NSSR_model_list_no_random_zi[[i]]): the
## effects of zero-inflation and dispersion model are ignored
```

```
## Warning: the null model is correct only if all variables used by the original
## model remain unchanged.
```

```
## Warning in r.squaredGLMM.glmmTMB(NSSR_model_list_no_random_zi[[i]]): the
## effects of zero-inflation and dispersion model are ignored
```

```
## Warning: the null model is correct only if all variables used by the original
## model remain unchanged.
```

```
## Warning in r.squaredGLMM.glmmTMB(NSSR_model_list_no_random_zi[[i]]): the
## effects of zero-inflation and dispersion model are ignored
```

```
## Warning: the null model is correct only if all variables used by the original
## model remain unchanged.
```

```
## Warning in r.squaredGLMM.glmmTMB(NSSR_model_list_no_random_zi[[i]]): the
## effects of zero-inflation and dispersion model are ignored
```

```
## Warning: the null model is correct only if all variables used by the original
## model remain unchanged.
```

```
## Warning in r.squaredGLMM.glmmTMB(NSSR_model_list_no_random_zi[[i]]): the
## effects of zero-inflation and dispersion model are ignored
```

```
## Warning: the null model is correct only if all variables used by the original
## model remain unchanged.
```

```
## Warning in r.squaredGLMM.glmmTMB(NSSR_model_list_no_random_zi[[i]]): the
## effects of zero-inflation and dispersion model are ignored
```

```
## Warning: the null model is correct only if all variables used by the original
## model remain unchanged.
```

```
## Warning in r.squaredGLMM.glmmTMB(NSSR_model_list_no_random_zi[[i]]): the
## effects of zero-inflation and dispersion model are ignored
```

```
## Warning: the null model is correct only if all variables used by the original
## model remain unchanged.
```

```
## Warning in r.squaredGLMM.glmmTMB(NSSR_model_list_no_random_zi[[i]]): the
## effects of zero-inflation and dispersion model are ignored
```

```
## Warning: the null model is correct only if all variables used by the original
## model remain unchanged.
```

```
## Warning in r.squaredGLMM.glmmTMB(NSSR_model_list_no_random_zi[[i]]): the
## effects of zero-inflation and dispersion model are ignored
```

```
## Warning: the null model is correct only if all variables used by the original
## model remain unchanged.
```

```
## Warning in r.squaredGLMM.glmmTMB(NSSR_model_list_no_random_zi[[i]]): the
## effects of zero-inflation and dispersion model are ignored
```

```
## Warning: the null model is correct only if all variables used by the original
## model remain unchanged.
```

```
## Warning in r.squaredGLMM.glmmTMB(NSSR_model_list_no_random_zi[[i]]): the
## effects of zero-inflation and dispersion model are ignored
```

```
## Warning: the null model is correct only if all variables used by the original
## model remain unchanged.
```

Results from the models for *with random variable* and
*without zero inflation*

```
for (i in seq_along(NSSR_model_list_with_random)){
  
  res <- summary(NSSR_model_list_with_random[[i]])
  
  NSSR_summary.models[,1:8] <- c(NSSR_model_list_with_random_notation[[i]], 
                                 res$AICtab[1],
                                 AICcmodavg::AICc(NSSR_model_list_with_random[[i]]),
                                 res$AICtab[2],
                                 as.data.frame(MuMIn::r.squaredGLMM(NSSR_model_list_with_random[[i]]))$R2m[1],
                                 as.data.frame(MuMIn::r.squaredGLMM(NSSR_model_list_with_random[[i]]))$R2c[1],
                                 "yes",
                                 "no"
  )
  
  NSSR_summary.models.with.random[[i]] <- NSSR_summary.models
}
```

Results from the models for *with random variable* and
*with zero inflation*

```
for (i in seq_along(NSSR_model_list_with_random_zi)){
  
  res <- summary(NSSR_model_list_with_random_zi[[i]])
  
  NSSR_summary.models[,1:8] <- c(NSSR_model_list_with_random_notation_zi[[i]], 
                                 res$AICtab[1],
                                 AICcmodavg::AICc(NSSR_model_list_with_random_zi[[i]]),
                                 res$AICtab[2],
                                 as.data.frame(MuMIn::r.squaredGLMM(NSSR_model_list_with_random_zi[[i]]))$R2m[1],
                                 as.data.frame(MuMIn::r.squaredGLMM(NSSR_model_list_with_random_zi[[i]]))$R2c[1],
                                 "yes",
                                 "yes"
  )
  
  NSSR_summary.models.with.random_zi[[i]] <- NSSR_summary.models
}
```

## Comparing the models

Best model is selected based on AICc.

```
library(dplyr)

NSSR_data1 <- do.call(bind_rows, NSSR_summary.models.no.random)
NSSR_data2 <- do.call(bind_rows, NSSR_summary.models.no.random_zi)
NSSR_data3 <- do.call(bind_rows, NSSR_summary.models.with.random)
NSSR_data4 <- do.call(bind_rows, NSSR_summary.models.with.random_zi)

NSSR_summary.models <- bind_rows(NSSR_data1,NSSR_data2,NSSR_data3,NSSR_data4)

NSSR_summary.models$Model <- paste0("Model.", seq_len(nrow(NSSR_summary.models)))

NSSR_summary.models[, c(2:6)] <- lapply(NSSR_summary.models[, c(2:6)], as.numeric)

NSSR_summary.models$Delta_AICc <- NSSR_summary.models$AICc-(min(NSSR_summary.models$AICc, na.rm = T))

# Which is the best model based on AICc?
(NSSR_best_model <- NSSR_summary.models[order(NSSR_summary.models$AICc), ][1,])
```

```
##                                                                                                                                  formula
## 270 D1.Native.Salmonidae.richness~scales::rescale(log1p(Maximum_depth_m)) + scales::rescale(log1p(distance_to_refugium)) + (1|Catchment)
##         AIC     AICc      BIC R2.mar.mumin R2.cond.mumin random_var zi
## 270 256.017 256.8389 267.8642    0.3215563     0.6963239        yes no
##         Model Delta_AICc
## 270 Model.270          0
```

```
# Saving the results of the model comparisons
write.csv(NSSR_summary.models, "NSSR_ranked_glmm.models.csv")
```

## Assessing the results from the best model

Once we have ranked the models and selected the best, we assessed the
results.

```
NSSR_summary.models <- read.csv("NSSR_ranked_glmm.models.csv")
NSSR_summary.models$X <- NULL

NSSR_best.model <- glmmTMB(formula =
                             as.formula(NSSR_summary.models[order(NSSR_summary.models$AICc), ][1,1]),
                           ziformula = ~ 0,
                           family = nbinom2, 
                           REML=F, 
                           data = data)
```

```
## Warning in finalizeTMB(TMBStruc, obj, fit, h, data.tmb.old): Model convergence
## problem; non-positive-definite Hessian matrix. See vignette('troubleshooting')
```

```
## Warning in finalizeTMB(TMBStruc, obj, fit, h, data.tmb.old): Model convergence
## problem; false convergence (8). See vignette('troubleshooting'),
## help('diagnose')
```

Addressing model convergence problem.

```
NSSR_best.model <- update(NSSR_best.model, 
                              control=glmmTMBControl(optimizer=optim, optArgs=list(method="BFGS")))
```

The best model contains: 1. Two explanatory variables: *maximum
depth (m)* and *distance to glacial refugium (m)* 2. The
random variable: *catchment* 3. No zero inflation component

*Random variable* The presence of the random variable suggests
the intercept varies among catchments. The variance is 0.8186 while the
variance standard deviation is 0.9048; therefore, there is indeed an
apparently meaningful variability accross catchments in the baseline of
endemism proportion.

*Dispersion parameter* The dispersion parameter is 3.41e+09.
As explained before,this large value suggests low dispersion.

```
summary(NSSR_best.model)
```

```
##  Family: nbinom2  ( log )
## Formula:          
## D1.Native.Salmonidae.richness ~ scales::rescale(log1p(Maximum_depth_m)) +  
##     scales::rescale(log1p(distance_to_refugium)) + (1 | Catchment)
## Data: data
## 
##      AIC      BIC   logLik deviance df.resid 
##    256.0    267.9   -123.0    246.0       74 
## 
## Random effects:
## 
## Conditional model:
##  Groups    Name        Variance Std.Dev.
##  Catchment (Intercept) 0.5239   0.7238  
## Number of obs: 79, groups:  Catchment, 4
## 
## Dispersion parameter for nbinom2 family (): 6.03e+04 
## 
## Conditional model:
##                                              Estimate Std. Error z value
## (Intercept)                                   -2.2137     0.6501  -3.405
## scales::rescale(log1p(Maximum_depth_m))        2.8030     0.3925   7.141
## scales::rescale(log1p(distance_to_refugium))   1.9779     0.8135   2.431
##                                              Pr(>|z|)    
## (Intercept)                                  0.000661 ***
## scales::rescale(log1p(Maximum_depth_m))      9.24e-13 ***
## scales::rescale(log1p(distance_to_refugium)) 0.015047 *  
## ---
## Signif. codes:  0 '***' 0.001 '**' 0.01 '*' 0.05 '.' 0.1 ' ' 1
```

# Model diagnostics,

For residulas diagnostics and formal assessement of dispersal and
zero inflation, we used DHARMa. Because the model contains only one
variable, there was no need to re-test for multicollinearity

```
library(DHARMa)

# Residual diagnostics:
NSSR_res <- simulateResiduals(fittedModel = NSSR_best.model, n = 10000)
plot(NSSR_res, rank = TRUE)
```

```
# Confirming that our model small residual variation (underdispersed):
testDispersion(NSSR_res)
```

```
## 
##  DHARMa nonparametric dispersion test via sd of residuals fitted vs.
##  simulated
## 
## data:  simulationOutput
## dispersion = 0.71392, p-value = 0.7146
## alternative hypothesis: two.sided
```

```
# Checking that model’s handling of zeros is fine.
testZeroInflation(NSSR_res)
```

```
## 
##  DHARMa zero-inflation test via comparison to expected zeros with
##  simulation under H0 = fitted model
## 
## data:  simulationOutput
## ratioObsSim = 0.80826, p-value = 0.7992
## alternative hypothesis: two.sided
```

Making preliminary plots to visualize the effect of maximum depth and
distance to refugium on salmonid richness.

```
library(ggeffects)
library(ggplot2)

NSSR_predicted_max_depth <- predict_response(NSSR_best.model, terms = "Maximum_depth_m [all]")
NSSR_predicted_ref <- predict_response(NSSR_best.model, terms = "distance_to_refugium [all]")

ggplot(NSSR_predicted_max_depth, aes(x = x, y = predicted)) +
  geom_line() +
  geom_ribbon(aes(ymin = conf.low, ymax = conf.high), alpha = 0.2)
```

```
ggplot(NSSR_predicted_ref, aes(x = x, y = predicted)) +
  geom_line() +
  geom_ribbon(aes(ymin = conf.low, ymax = conf.high), alpha = 0.2)
```

## Plots predicted values from GLMM

Below is the code used to generate the figures used in the final
pu§blication.

#Total native species richness - TNSR

```
# TNSR ~ surface area
TNSR_area_plot <- ggplot(TNSR_predicted_area, aes(x = x, y = predicted)) +
  geom_line(linewidth = 0.5, color = 'black') +  
  geom_ribbon(aes(ymin = conf.low, ymax = conf.high), alpha = 0.2) +
  labs(x = "Surface area (Km2)", 
       y = "Total native richness") +
  geom_point(data = data, aes(x = Surface_area_km2, y = D1.Total_native_Richness, 
                              size = (Surface_area_km2), color = Catchment), alpha = 0.5) +
  theme_classic() +
  scale_x_log10() +
  scale_size_continuous(
    name = "Surface area (Km2)"
  ) +
  theme(
    axis.title = element_text(size = 10, color = 'black'),  
    axis.text = element_text(size = 10, color = 'black')
  )  +
  scale_color_discrete(name = "Catchment") +
  guides(size = "none", color = "none")

# TNSR ~ maximum depth
TNSR_depth_plot <- ggplot(TNSR_predicted_max_depth, aes(x = x, y = predicted)) +
  geom_line(linewidth = 0.5, color = 'black') +  
  geom_ribbon(aes(ymin = conf.low, ymax = conf.high), alpha = 0.2) +
  labs(x = "Maximum depth (m)", 
       y = "Total native richness") +
  geom_point(data = data, aes(x = Maximum_depth_m, y = D1.Total_native_Richness,
                                  size = (Surface_area_km2), color = Catchment), alpha = 0.5) +
  theme_classic() +
  scale_x_log10() +
  # scale_y_log10() +
  scale_size_continuous(
    name = "Surface area (Km2)"
  ) +
  theme(
    axis.title = element_text(size = 10, color = 'black'),  
    axis.text = element_text(size = 10, color = 'black')
  )  +
  scale_color_discrete(name = "Catchment") +
  guides(size = "none", color = "none")

# TNSR ~ temperature
TNSR_temp_plot <- ggplot(TNSR_predicted_temp, aes(x = x, y = predicted)) +
  geom_line(linewidth = 0.5, color = 'black') +
  geom_ribbon(aes(ymin = conf.low, ymax = conf.high), alpha = 0.2) +
  labs(x = "Average surface temperature (°C)",
       y = "Total native richness") +
  geom_point(data = data, aes(x = avg_surface_temp_domischetal2015, y = D1.Total_native_Richness, size = (Surface_area_km2), color = Catchment), alpha = 0.5) +
  theme_classic() +
  scale_x_log10() +
  # scale_y_log10() +
  scale_size_continuous(
    name = "Surface area (Km2)"
  ) +
  theme(
    axis.title = element_text(size = 10, color = 'black'),
    axis.text = element_text(size = 10, color = 'black')
  )  +
  scale_color_discrete(name = "Catchment") +
  # guides(color = guide_legend(override.aes = list(size = 5))) 
  guides(size = "none", color = "none")

### Plotting the estimates (fixed effects)
# Define custom labels
custom_labels <- c(
  "scales::rescale(log1p(Maximum_depth_m))" = "Maximum Depth",
  "scales::rescale(log1p(avg_surface_temp_domischetal2015))" = "Average surface temperature",
  "scales::rescale(log1p(distance_to_refugium))" = "Distance to glacial refugium",
  "scales::rescale(log1p(Surface_area_km2))" = "Surface area"
)

# Customizing colors
color.total.rich <- '#0072B2'
color.salmonid.rich <- '#D55E00'
color.endemism.prop <- "#CC79A7"

library(sjPlot)

(
  TNSR_estimates <- plot_models(TNSR_best.model, 
                                     transform = NULL,
                                     vline.color = "dark grey",
                                     title = "Total species richness",
                                     axis.title = "Standardized coefficient",
                                     colors = color.total.rich,
                                     show.legend = F, 
                                     show.values = TRUE,
                                     axis.labels = custom_labels, 
                                     axis.lim = c(-1,2)
                                   ) +
  theme_bw() +
  theme(title = element_text(size = 10, hjust = 0.5),
        axis.title = element_text(size = 10, color = 'black'),
        axis.text = element_text(size = 10, color = 'black'))
)
```

#Proportion of endemic species - PEnS

```
PEnS_depth_plot <- ggplot(PEnS_predicted_max_depth, aes(x = x, y = predicted)) +
  geom_line(size = .5, color = 'black') +  
  geom_ribbon(aes(ymin = conf.low, ymax = conf.high), alpha = 0.2) +
  labs(x = "Maximum depth (m)", 
       y = "Endemism proportion") +
  geom_point(data = data, aes(x = Maximum_depth_m, y = Endemism_proportion, size = (Surface_area_km2), color = Catchment), alpha = 0.5) +
  theme_classic() +
  scale_x_log10() + 
  scale_size_continuous(
    name = "Surface area (Km2)"
  ) +
  theme(
    axis.title = element_text(size = 10, color = color.endemism.prop),  
    axis.text = element_text(size = 10, color = color.endemism.prop),
    axis.line = element_line(color = color.endemism.prop), 
    axis.ticks = element_line(color = color.endemism.prop),
    legend.title = element_text(size = 10)
  )  +
  scale_color_discrete(name = "Catchment") +
  guides(size = "none", color = "none")
```

```
## Warning: Using `size` aesthetic for lines was deprecated in ggplot2 3.4.0.
## ℹ Please use `linewidth` instead.
## This warning is displayed once every 8 hours.
## Call `lifecycle::last_lifecycle_warnings()` to see where this warning was
## generated.
```

```
### Plotting the estimates (fixed effects)
(
  PEnS_estimates <- plot_models(PEnS_best.model, 
                                        transform = NULL,
                                        vline.color = "dark grey",
                                        title = "Endemism proportion",
                                        axis.title = "Standardized coefficient",
                                        colors = color.endemism.prop,
                                        show.legend = F, 
                                        show.values = TRUE,
                                        axis.labels = custom_labels) +
  theme_bw() +
  theme(title = element_text(size = 10, hjust = 0.5),
        axis.title = element_text(size = 10, color = 'black'),
        axis.text = element_text(size = 10, color = 'black'))
  )
```

#Total native salmonid richness

```
NSSR_depth_plot <- ggplot(NSSR_predicted_max_depth, aes(x = x, y = predicted)) +
  geom_line(size = 0.5, color = 'black') +  
  geom_ribbon(aes(ymin = conf.low, ymax = conf.high), alpha = 0.2) +
  labs(x = "Maximum depth (m)", 
       y = "Salmonidae richness") +
  geom_point(data = data, aes(x = Maximum_depth_m, y = D1.Native.Salmonidae.richness, size = (Surface_area_km2), color = Catchment), alpha = 0.5) +
  theme_classic() +
  scale_x_log10() +
  # scale_y_log10() +
  scale_size_continuous(
    name = "Surface area (Km2)",
  ) +
  theme(
    axis.title = element_text(size = 10, color = color.salmonid.rich),  
    axis.text = element_text(size = 10, color = color.salmonid.rich),
    axis.line = element_line(color = color.salmonid.rich),
    axis.ticks = element_line(color = color.salmonid.rich)
  )  +
  scale_color_discrete(name = "Catchment") +
  guides(size = "none", color = "none")

NSSR_refugium_plot <- ggplot(NSSR_predicted_ref, aes(x = x, y = predicted)) +
  geom_line(size = 0.5, color = 'black') +  
  geom_ribbon(aes(ymin = conf.low, ymax = conf.high), alpha = 0.2) +
  labs(x = "Distance to refugium (Km)", 
       y = "Salmonidae richness") +
  geom_point(data = data, aes(x = distance_to_refugium, y = D1.Native.Salmonidae.richness, size = (Surface_area_km2), color = Catchment), alpha = 0.5) +
  theme_classic() +
  scale_x_log10() +
  # scale_y_log10() +
  scale_size_continuous(
    name = "Surface area (Km2)"
  ) +
  theme(
    axis.title = element_text(size = 10, color = color.salmonid.rich),  
    axis.text = element_text(size = 10, color = color.salmonid.rich),
    axis.line = element_line(color = color.salmonid.rich),
    axis.ticks = element_line(color = color.salmonid.rich)
  )  +
  scale_color_discrete(name = "Catchment") +
  # guides(color = guide_legend(override.aes = list(size = 5))) +
guides(size = "none", color = "none")

# Plotting the estimates

(NSSR_estimates <- plot_models(NSSR_best.model, 
                                        transform = NULL,
                                        vline.color = "darkgray",
                                        title = "Salmonidae richness",
                                        axis.title = "Standardized coefficient",
                                        colors = color.salmonid.rich,
                                        show.legend = F, 
                                        show.values = TRUE,
                                        axis.labels = custom_labels,
                               axis.lim = c(-1,4)) +
  theme_bw() +
  theme(title = element_text(size = 10, hjust = 0.5),
        axis.title = element_text(size = 10, color = 'black'),
        axis.text = element_text(size = 10, color = 'black'))
  )
```

With the code below, we created a plot with the legend that will be
recycled for the full figure.

```
# Function to get the legend
get_legend <- function(myggplot) {
  tmp <- ggplot_gtable(ggplot_build(myggplot))
  leg <- which(sapply(tmp$grobs, function(x) x$name) == "guide-box")
  legend <- tmp$grobs[[leg]]
  return(legend)
}

# Create the plot with the legend
plot_for_legend <-  ggplot(NSSR_predicted_ref, aes(x = x, y = predicted)) +
  geom_line(size = .5, color = 'black') +  
  geom_ribbon(aes(ymin = conf.low, ymax = conf.high), alpha = 0.2) +
  labs(x = "Distance to glacial refugium", 
       y = "Salmonidae richness") +
  geom_point(data = data, 
             aes(x = distance_to_refugium, 
                 y = D1.Native.Salmonidae.richness, 
                 size = Surface_area_km2, 
                 color = Catchment), 
             alpha = 0.5) +
  theme_classic() +
  scale_size_continuous(
    name = "Surface area (Km2)"
  ) +
  theme(
    axis.title = element_text(size = 10, color = color.endemism.prop),  
    axis.text = element_text(size = 10, color = color.endemism.prop),
    axis.line = element_line(color = color.endemism.prop), 
    axis.ticks = element_line(color = color.endemism.prop),
    legend.title = element_text(size = 10)
  ) +
  scale_color_discrete(name = "Catchment")

# Extract the legend
legend <- get_legend(plot_for_legend)
```

In the panel D of Figure 2, we also included the fit using rational
function, S=(c+zA)/(1+dA), which was the best fit from a multimodel test
for the entire dataset; fit of the log-log version of the power model
for the entire dataset and for individual catchments.

```
# These are the three parameters for the rational function:
c.rat <- 7.23115960146551
z.rat <- 1.44280352640841
d.rat <- 0.0429399110374847

# This is to create a transparent color pallete for the catchment fits
library(scales)
catchment_levels <- levels(factor(data$Catchment))
n_catchments <- length(catchment_levels)
catchment_colors <- alpha(hue_pal()(n_catchments), 0.35)  # 0.3 = 30% opacity
names(catchment_colors) <- catchment_levels


TNSR_area_plot_updated <- 
  TNSR_area_plot +
  
  # Rational function
  stat_function(fun = function(A) (c.rat + z.rat * A) / (1 + d.rat * A), 
                color = "blue", linetype = "dashed", size = 0.8) + 

  # Power model - full dataset
  geom_smooth(data = data, 
              method = "lm", 
              se = F, 
              aes(x = Surface_area_km2, 
                  y = D1.Total_native_Richness), 
              color = alpha("darkgrey", 0.7),
              # alpha = 0.75,
              lwd = 0.75, 
              show.legend = FALSE)  +
    
  # Power model - individual catchments
 geom_smooth(data = data, 
              method = "lm", 
              se = FALSE, 
              aes(x = Surface_area_km2, 
                  y = D1.Total_native_Richness, 
                  color = Catchment, 
                  fill = Catchment), 
              lwd = 1, 
              show.legend = FALSE) +

  scale_color_manual(values = catchment_colors)
```

```
## Scale for colour is already present.
## Adding another scale for colour, which will replace the existing scale.
```

Then, combine all figures in one:

```
predictions_ggeffects <- cowplot::plot_grid(TNSR_area_plot_updated,
                                            TNSR_depth_plot,
                                            TNSR_temp_plot,
                                            legend,
                                            PEnS_depth_plot, 
                                            NSSR_depth_plot,
                                            NSSR_refugium_plot,
                                            ncol = 4)
```

```
## `geom_smooth()` using formula = 'y ~ x'
## `geom_smooth()` using formula = 'y ~ x'
```

```
prediction_estimates <- cowplot::plot_grid(TNSR_estimates,
                                           PEnS_estimates,
                                           NSSR_estimates,
                                           ncol = 1)

predictons_comb <- cowplot::plot_grid(prediction_estimates, 
                                      predictions_ggeffects,
                                      rel_widths = c(1,2,1),
                                      col = 3)
```

```
## Warning in as_grob.default(plot): Cannot convert object of class numeric into a
## grob.
```

# Plotting and saving the figures

```
predictons_comb

pdf("Fig2_combined_plos_isars.pdf", height = 12, width = 15)
predictons_comb
dev.off()
system("open Fig2_combined_plos_isars.pdf")
```
