# Supplementary material for: Recent and Rapid Assembly of an Island Species–Area Relationship Threatened by Human Disturbance
Source: Ecol Lett. 2025 Oct 5;28(10):e70222. doi: 10.1111/ele.70222 (PMC12498083; doi:10.1111/ele.70222)
Supplement: Supplementary file 5 — Data S5: ele70222‐sup‐0005‐Supinfo5.html. [file ELE-28-0-s004.html]

ISAR Multimodel Analysis – Peri-Alpine Lakes


# ISAR Multimodel Analysis – Peri-Alpine Lakes

#### 2025-04-25

## Multimodel analysis

This script tests for the best mathematical function to describe the
ISAR shape for the fish community of the peri-Alpine lakes

The package (R functions) were written by Rampal Etienne and it’s
available to download:

```
packages <- c("remotes", "DDD", "knitr", "isars", "ggplot2")

# Install missing CRAN packages
cran_pkgs <- setdiff(packages, rownames(installed.packages()))
cran_pkgs <- setdiff(cran_pkgs, "isars")

if (length(cran_pkgs)) install.packages(cran_pkgs)

# Install isars from GitHub if missing
if (!"isars" %in% rownames(installed.packages())) {
  if (!"remotes" %in% rownames(installed.packages())) {
    install.packages("remotes")
  }
  remotes::install_github("rsetienne/isars")
}

# Load packages
lapply(packages, library, character.only = TRUE)
```

```
## [[1]]
## [1] "remotes"   "stats"     "graphics"  "grDevices" "utils"     "datasets" 
## [7] "methods"   "base"     
## 
## [[2]]
## [1] "DDD"       "remotes"   "stats"     "graphics"  "grDevices" "utils"    
## [7] "datasets"  "methods"   "base"     
## 
## [[3]]
##  [1] "knitr"     "DDD"       "remotes"   "stats"     "graphics"  "grDevices"
##  [7] "utils"     "datasets"  "methods"   "base"     
## 
## [[4]]
##  [1] "isars"     "knitr"     "DDD"       "remotes"   "stats"     "graphics" 
##  [7] "grDevices" "utils"     "datasets"  "methods"   "base"     
## 
## [[5]]
##  [1] "ggplot2"   "isars"     "knitr"     "DDD"       "remotes"   "stats"    
##  [7] "graphics"  "grDevices" "utils"     "datasets"  "methods"   "base"
```

The raw richness data is already attached to the package.

```
isar_data <- read.csv(system.file("extdata", 
                                  "lake_area_richness.csv", 
                                  package = "isars")
                      )
```

Richness data are as follows:

1. **D1.OC**: Total Native Species Richness (TNSR). That’s
   what we consider to be the original community (OC);
2. **D2.OC\_Int**: Total Species Richness. That’s the OC
   adding non-native species that have been introduced: TNSR + introduced
   species;
3. **D3.OC\_Ext**: Total Native **Extant**
   Species Richness. That’s the OC removing extinct native species;
4. **D6.CC**: Total Species Richness of the Contemporary
   community(CC). That’s OC + Introduced species - Extinct species.

**Surface\_area\_Km2** represents the surface area of each
lake in the dataset.

```
head(isar_data)
```

```
##         Lake Catchment     Long      Lat Surface_area_km2 D1.OC D2.OC_Inr
## 1   Achensee    Danube 11.70907 47.49736             6.80    13        16
## 2      Aeger     Rhine  8.59000 47.14000             7.30    20        22
## 3     Almsee    Danube 13.95816 47.76032             0.85     6         6
## 4 Altausseer    Danube 13.76972 47.63661             2.10     7         8
## 5     Annecy     Rhone  6.13000 45.90000            27.60    10        13
## 6   Attersee    Danube 13.59231 47.94829            46.20    16        20
##   D3.OC_Ext D6.CC
## 1        13    16
## 2        20    22
## 3         6     6
## 4         7     8
## 5        10    13
## 6        14    18
```

For each **richness** dataset described above, we tested
for the best mathematical function to describe the ISAR using 24
candidate models listed in Supporting information (Tables S1-S4).

## Best model to describe TNSR

Here you will run only on the original community (TNSR) To run for
the other three datasets, change `type_of_richness`.

```
richness_metrics <- names(isar_data)[6:9]
```

First, let’s identify our variables:

```
type_of_richness <- richness_metrics[1]
area <- isar_data[, "Surface_area_km2"]
obs_richness <- isar_data[, type_of_richness]
```

Initial parameters to start the analysis for the runs are available
here:

```
isar_initial_pars <- read.csv(system.file("extdata", 
                                          "isar_initial_pars.csv", 
                                          package = "isars")
                              )

isar_initial_pars21 <- c(c = 8.63, T1 = 4.1, z1 = 1.68, z2 = 5.08)
isar_initial_pars22 <- c(c = 8.63, T1 = 4.1, T2 = 21.14, 
                         z1 = 1.68, z2 = 5.08, z3 = 5.08)
isar_initial_pars23 <- c(c = 8.63, T1 = 4.1, z2 = 5.08)
isar_initial_pars24 <- c(c = 8.65, T1 = 4.1, T2 = 21.14, 
                         z2 = 5.08, z3 = 5.08)
isar_initial_pars25 <- c(c = 8.63, T1 = 0.1, z1 = 5.08)

  isar_initial_pars[17, "c"] <- isar_initial_pars[16, "c"]
  isar_initial_pars[17, "z"] <- isar_initial_pars[16, "z"]
  isar_initial_pars[17, "f"] <- 0.1
```

For this analysis, we removed the Chapman model:

```
isar_initial_pars <- isar_initial_pars[-3, ]

isar_model_names <- list("f_asymp", 
                         "f_betap", 
                         # "f_chapman",
                         "f_loga", 
                         "f_epm1", 
                         "f_epm2", 
                         "f_gompertz",
                         "f_koba", 
                         "f_linear",
                         "f_heleg", 
                         "f_monod", 
                         "f_mmf",
                         "f_negexpo", 
                         "f_p1", 
                         "f_p2", 
                         "f_power",
                         "f_powerR", 
                         "f_ratio", 
                         "f_weibull3", 
                         "f_weibull4",
                         "f_ContOne",
                         "f_ContTwo", 
                         "f_ContOne", # f_ZslopeOne
                         "f_ContTwo", # f_ZslopeTwo
                         "f_ContOne2") # f_RightZslopeOne

isar_models <- lapply(isar_model_names, function(name) get(name, envir = asNamespace("isars")))
```

With the code below, we fitted the models using maximum likelihood,
where the likelihood is the product of the Poisson probability of the
observed richness values with its mean value given by the ISAR for each
area. Note that for here we are using only 1 random initial parameter
set. For the original analysis, we set this number to 10.

```
num.init <- 1 # change to 10
seed.number <- 42
sd <- 0.1

out <- list()

for (i in 1:24) {
  if (i <= 19) {
    NAs <- which(is.na(isar_initial_pars[i, ]))
    if (length(NAs) > 0) {
      initparsopt <- isar_initial_pars[i, -NAs]
    }
    else {
      initparsopt <- isar_initial_pars[i, ]
    }
    idparsopt <- 1:length(initparsopt)
    parsfix <- NULL
    idparsfix <- NULL
  }
  else {
    if (i == 20) {
      initparsopt <- isar_initial_pars21
      parsfix <- NULL
      idparsopt <- 1:length(initparsopt)
      idparsfix <- NULL
    }
    if (i == 21) {
      initparsopt <- isar_initial_pars22
      parsfix <- NULL
      idparsopt <- 1:length(initparsopt)
      idparsfix <- NULL
    }
    if (i == 22) {
      initparsopt <- isar_initial_pars23
      parsfix <- c(z1 = 0)
      idparsopt <- c(1, 2, 4)
      idparsfix <- 3
    }
    if (i == 23) {
      initparsopt <- isar_initial_pars24
      parsfix <- c(z1 = 0)
      idparsopt <- c(1:3, 5:6)
      idparsfix <- 4
    }
    if (i == 24) {
      initparsopt <- isar_initial_pars25
      parsfix <- NULL
      idparsopt <- 1:length(initparsopt)
      idparsfix <- NULL
    }
  }
  
  names_pars <- names(initparsopt)
  initparsopt <- as.numeric(initparsopt)
  names(initparsopt) <- names_pars
  names_pars <- names(parsfix)
  parsfix <- as.numeric(parsfix)
  names(parsfix) <- names_pars
  
  out[[i]] <- list(initpars = rep(0, length(idparsopt) + 
                                    length(idparsfix)), model = NULL, fit = NULL)
  
  out[[i]]$initpars[idparsopt] <- initparsopt
  
  if (length(idparsfix) > 0) 
    out[[i]]$initpars[idparsfix] <- parsfix
  out[[i]]$model <- isar_models[[i]]
  
tmp <- capture.output({
  out[[i]]$fit <- isar_ML(f_isar = isar_models[[i]], 
                          initparsopt = initparsopt, 
                          parsfix = parsfix, 
                          idparsopt = idparsopt,
                          idparsfix = idparsfix, 
                          area = area, 
                          obs_richness = obs_richness,
                          trial_settings = c(num.init, seed.number, sd), 
                          verbose = TRUE)
})

}
```

Then, calculate AIC weights.

```
fit_results <- as.data.frame(matrix(NA, nrow = length(isar_model_names), 
                                    ncol = 10))

names(fit_results) <- c("model", "c", "d or T1", "f or T2", 
                        "z or z1", "z2", "z3", "ML", "AICc", "AICcweight")

for (i in 1:24) {
  vec <- out[[i]]$fit
  lvec <- length(vec)
  fit_results[i, 1] <- isar_model_names[[i]]
  fit_results[i, 8:9] <- vec[(length(vec) - 1):length(vec)]
  if (!is.na(vec["c"])) 
    fit_results[i, "c"] <- vec["c"]
  if (!is.na(vec["d"])) 
    fit_results[i, "d or T1"] <- vec["d"]
  if (!is.na(vec["f"])) 
    fit_results[i, "f or T2"] <- vec["f"]
  if (!is.na(vec["z"])) 
    fit_results[i, "z or z1"] <- vec["z"]
  if (!is.na(vec["z1"])) 
    fit_results[i, "z or z1"] <- vec["z1"]
  if (!is.na(vec["z2"])) 
    fit_results[i, "z2"] <- vec["z2"]
  if (!is.na(vec["z3"])) 
    fit_results[i, "z3"] <- vec["z3"]
  if (!is.na(vec["T1"])) 
    fit_results[i, "d or T1"] <- vec["T1"]
  if (!is.na(vec["T2"])) 
    fit_results[i, "f or T2"] <- vec["T2"]
}


ICweights <- get("ICweights", envir = asNamespace("isars"))
fit_results[, 10] <- ICweights(IC = fit_results[, 9])
fit_results[22, 1] <- "f_ZslopeOne"
fit_results[23, 1] <- "f_ZslopeTwo"
fit_results[24, 1] <- "f_RightZslopeOne"

# write.csv(fit_results, 
#           paste0("/Volumes/Saci/Naturalis/sar_project/isar_etienne_package/",
#                  type_of_richness, "_", "isar_results.csv"), row.names = FALSE)

fit_results
```

```
##               model            c      d or T1      f or T2    z or z1       z2
## 1           f_asymp 2.263078e+01 3.032667e+01           NA 0.96411106       NA
## 2           f_betap 2.685189e+11 4.485900e+01 1.068345e+03 0.32543274       NA
## 3            f_loga 8.721193e+00           NA           NA 3.43602144       NA
## 4            f_epm1 8.395620e+00 3.301760e-02           NA 0.29028773       NA
## 5            f_epm2 8.629227e+00 2.659058e-02           NA 0.24339500       NA
## 6        f_gompertz 4.276291e+00 2.926245e+01           NA 0.05917370       NA
## 7            f_koba 3.787676e+00           NA           NA 0.12577093       NA
## 8          f_linear 1.209750e+01           NA           NA 0.07275887       NA
## 9           f_heleg 9.438430e+00           NA 1.348179e-01 0.32994615       NA
## 10          f_monod 2.018593e+00 2.287847e+01           NA         NA       NA
## 11            f_mmf 7.417416e+00 7.000877e+01           NA 0.32994606       NA
## 12        f_negexpo           NA 2.016429e+01           NA 0.46260573       NA
## 13             f_p1 8.274466e+00 6.584090e-04           NA 0.27768512       NA
## 14             f_p2 8.679402e+00 1.080575e-13           NA 0.24103296       NA
## 15          f_power 8.679404e+00           NA           NA 0.24103287       NA
## 16         f_powerR 8.679402e+00           NA 1.829263e-13 0.24103296       NA
## 17          f_ratio 7.231158e+00 4.293990e-02           NA 1.44280325       NA
## 18       f_weibull3 2.039068e-01 4.484842e+01           NA 0.32538199       NA
## 19       f_weibull4 7.490527e-06 3.265143e+01 1.161746e-01 2.33969368       NA
## 20        f_ContOne 8.787466e+00 2.433613e+00           NA 2.45683220 5.827207
## 21        f_ContTwo 8.787621e+00 2.433613e+00 2.608225e+01 2.45670618 5.827288
## 22      f_ZslopeOne 8.607140e+00 9.921151e-01           NA 0.00000000 5.233563
## 23      f_ZslopeTwo 8.607143e+00 9.921155e-01 3.814042e+01 0.00000000 5.233561
## 24 f_RightZslopeOne 8.721194e+00 1.759251e+01           NA 3.43601717       NA
##          z3        ML     AICc   AICcweight
## 1        NA -244.7307 495.7813 9.953772e-02
## 2        NA -245.4899 499.5202 1.534926e-02
## 3        NA -253.1274 510.4128 6.619124e-05
## 4        NA -246.1025 498.5249 2.524730e-02
## 5        NA -246.7351 499.7902 1.341108e-02
## 6        NA -246.2647 498.8493 2.146709e-02
## 7        NA -250.8540 505.8658 6.429362e-04
## 8        NA -305.9669 616.0918 7.462821e-28
## 9        NA -245.6857 497.6913 3.830324e-02
## 10       NA -281.7230 567.6040 2.522855e-17
## 11       NA -245.6857 497.6913 3.830324e-02
## 12       NA -307.6144 619.3867 1.436853e-28
## 13       NA -244.5947 495.5094 1.140315e-01
## 14       NA -246.9298 500.1795 1.103902e-02
## 15       NA -246.9298 498.0174 3.254061e-02
## 16       NA -246.9298 500.1795 1.103902e-02
## 17       NA -243.3593 493.0386 3.922521e-01
## 18       NA -245.4895 497.2990 4.660485e-02
## 19       NA -244.4405 497.4215 4.383496e-02
## 20       NA -245.8074 500.1554 1.117295e-02
## 21 4.821489 -245.8074 504.7815 1.105649e-03
## 22       NA -245.0001 496.3203 7.602374e-02
## 23 6.082666 -245.0001 500.8222 8.005163e-03
## 24       NA -253.1274 512.5749 2.245460e-05
```
